# Genome-wide identification and analysis of non-specific Lipid Transfer Proteins in hexaploid wheat

**DOI:** 10.1038/s41598-018-35375-7

**Published:** 2018-11-20

**Authors:** Allan Kouidri, Ryan Whitford, Radoslaw Suchecki, Elena Kalashyan, Ute Baumann

**Affiliations:** 1 University of Adelaide, School of Agriculture, Food & Wine, Waite Campus, Urrbrae, South Australia 5064 Australia; 2 Commonwealth Scientific and Industrial Research Organization, Agriculture and Food, Waite Campus, Urrbrae, South Australia 5064 Australia

## Abstract

Non-specific Lipid Transfer Proteins (nsLTPs) are involved in numerous biological processes. To date, only a fraction of wheat (*Triticum aestivum* L.) nsLTPs (TaLTPs) have been identified, and even fewer have been functionally analysed. In this study, the identification, classification, phylogenetic reconstruction, chromosome distribution, functional annotation and expression profiles of *TaLTPs* were analysed. 461 putative *TaLTPs* were identified from the wheat genome and classified into five types (1, 2, C, D and G). Phylogenetic analysis of the TaLTPs along with nsLTPs from *Arabidopsis thaliana* and rice, showed that all five types were shared across species, however, some type 2 TaLTPs formed wheat-specific clades. Gene duplication analysis indicated that tandem duplications contributed to the expansion of this gene family in wheat. Analysis of RNA sequencing data showed that *TaLTPs* were expressed in most tissues and stages of wheat development. Further, we refined the expression profile of anther-enriched expressed genes, and identified potential *cis-*elements regulating their expression specificity. This analysis provides a valuable resource towards elucidating the function of TaLTP family members during wheat development, aids our understanding of the evolution and expansion of the TaLTP gene family and, additionally, provides new information for developing wheat male-sterile lines with application to hybrid breeding.

## Introduction

Plant non-specific Lipid Transfer Proteins (nsLTPs) are small and soluble proteins with the ability to transfer various lipid molecules between membranes *in vitro*. nsLTPs are characterized by an eight cysteine motif (8 CM) backbone with the general form C-Xn-C-Xn-CC-Xn-CXC-Xn-C-Xn-C^1^. The cysteine residues are linked by four disulphide bonds stabilizing a tertiary structure composed of four or five alpha helices, with a hydrophobic cavity where the lipid binding takes place^2^. In general, nsLTPs possess an N-terminal secretory signal peptide targeting the protein to the secretory pathway. In addition, some LTPs also carry a C-terminal signal sequence whereby a glycosylphosphatidylinositol-anchor (GPI-anchor) is post-translationally attached to the protein; The GPI-anchor tethers the peptide to the extracellular side of the plasma membrane^3^. nsLTPs have been reported to participate in various biological processes, such as plant signalling, plant defence against biotic and abiotic stresses, cuticular wax and cutin synthesis, seed maturation and sexual reproduction^4^.

Plant *nsLTPs* consist of a large multigene family found in all land plants and are abundantly expressed in most tissues. Initially, nsLTPs were divided into two major groups based on molecular weight of the mature protein: nsLTP1 (9 kDa) and nsLTP2 (7 kDa)^5^. These two groups differ in the disulphide bond linkages, nsLTP1 at Cys1-Cys6 and Cys5-Cys8 and nsLTP2 at Cys1-Cys5 and Cys6-Cys8. Recently, a new classification according to intron position, presence of GPI-anchor pro-peptide domain and amino acid sequence identity was proposed by Edstam *et al*.^6^. The system classified nsLTPs into 10 types, including five major types (Type 1, 2, C, D and G) and five minor types containing fewer members (E, F, H, J and K). nsLTPs have been reported by genome-wide analysis for several members of the *Poaceae*, including maize (*Zea mays*) (63 nsLTPs), rice (*Oriza sativa*) (77 nsLTPs) and sorghum (*Sorghum bicolor*) (58 nsLTPs)^7^. Previously, in wheat 156 nsLTPs were identified based on EST datasets^8^.

Recent studies have revealed the importance of nsLTPs for pollen development. In *A*. *thaliana*, RNA interference knock-down for *AtLTPg*.3 and *AtLTPg*.4 displayed deformed or sterile pollen grains^9^. Of the type C *nsLTPs*, *AtLTPc*.*1*, *AtLTPc*.*2* and *AtLTPc*.*3* have an anther specific expression restricted to the tapetal cell layer^10^. AtLTPc.3 was shown to be secreted into the anther locule whereby it ultimately becomes a constituent of the microspore surface. Double RNAi silencing of *AtLTPc*.*1* and *AtLTPc*.*3* affected intine morphology, however, pollen grains showed no reduction in fertility. Similarly to *A*. *thaliana nsLTP* genes, the maize (*Zea mays*) *Ms44* also encodes a type C LTP specifically expressed in tapetal cells with its silencing having no effect on fertility^11^. However, a mutation impairing the cleavage of the *Ms44* signal peptide and therefore blocking its secretion, results in a dominant male sterility phenotype. In contrast, silencing of the rice *OsC6*, an anther-specific LTP, resulted in reduced pollen fertility^12^. Different to what has been observed in rice, wheat *TaMs1* is a *nsLTP* type G, which shows expression specifically in anthers containing pre-meiosis to meiotic microspores^13,14^. Detailed examination of anthers derived from several deletion mutants (*ms1a*, *ms1b*, *ms1c*) and ethyl methanesulfonate (EMS)-derived mutants (*ms1d*, *ms1e*, *ms1f* and *ms1h*) revealed male sterility is a consequence of disrupted orbicule and pollen exine structure. The determination that wheat *TaMs1* is a single locus nuclear-encoded gene necessary for wheat male fertility represented a significant advance towards developing a hybrid wheat production system similar to the maize Seed Production Technology (SPT)^13,15^. In previous studies, only a small portion of nsLTPs from wheat were identified. Considering a wheat genome reference sequence is now approaching completion, an opportunity exists to initiate a genome-wide analysis of the *nsLTP* gene family for this species.

In this study we identified 461 putative nsLTPs in the bread wheat genome (cv. Chinese Spring). We conducted a comprehensive study on the phylogeny, genomic structure, chromosomal location and expression profiles of the *nsLTP* gene family in wheat. Our analysis provides new insights into the *TaLTP* gene family which will support future functional research of nsLTPs. We identified anther-enriched nsLTPs of likely involvement in pollen development. When combined with new gene-editing technologies, this opens opportunities for exploring new loci for inducing male sterility that has application to hybrid breeding.

## Results

### Identification and classification of wheat nsLTPs

A total of 461 putative wheat *nsLTPs* were identified in cv. Chinese Spring (Supplementary Table S1). Predicted *nsLTPs* were classified into five types according to Edstam *et al*.^6^ (Type 1, 2, C, D and G)*;* type 2 contained most members with 59.44% of wheat *nsLTPs*, followed by type G (18.66%), type D (12.36%), type 1 (8.46%) and type C (1.08%). The proportion of wheat *nsLTPs* types varies greatly from that reported in genome-wide analyses performed in *A*. *thaliana* (*AtLTPs*), rice (*OsLTPs*), maize (*ZmLTPs*) and sorghum (*SbLTPs*) (Table 1), mainly due to a higher proportion of type 2 *nsLTPs* in wheat.

**Table 1:** A summary of nsLTP genes in wheat, maize, sorghum, rice and *A*. *thaliana*.

	Wheat	Maize^a^	Sorghum^a^	Rice^a^	*A*. *thaliana*^a^
Type 1	39	8	9	18	13
Type 2	274	9	7	13	13
Type C	5	2	2	2	3
Type D	57	16	13	14	12
Type E	0	0	0	0	2
Type G	86	26	24	27	29
Single	0	2	3	3	7
Total nsLTPs	461	63	58	77	79

^a^nsLTPs members retrieved from Wei and Zhong (2014)^7^.

**Figure 1:**
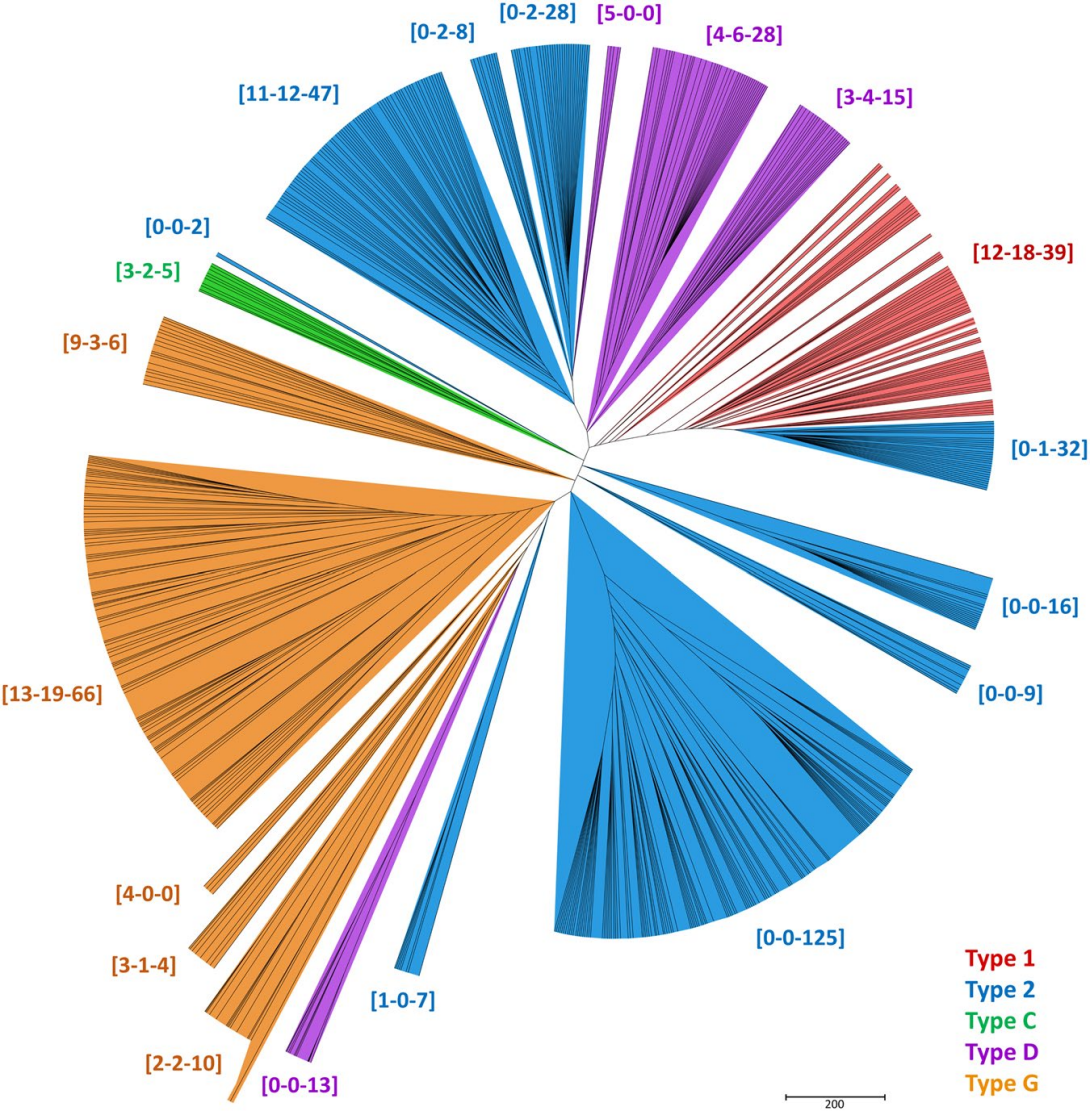
An unrooted phylogenetic tree of nsLTPs from *A*. *thaliana*, rice and bread wheat. The phylogenetic tree was built from an alignment of the predicted mature proteins. Brackets indicate the number of sequences from *A*. *thaliana*, rice and wheat, respectively. A detailed phylogenetic tree is provided in Supplementary Fig. S1.

The evolutionary relationship of nsLTPs between wheat, rice and *A*. *thaliana* was determined based on phylogenetic analysis (Figs 1 and S1). The tree organisation was in agreement and coherent with the organisation of the five types. For type 1 and type C, all sequences belonging to the same type were grouped and constitute monophyletic groups (i.e. clades). However, type 2, type D and Type G sequences were divided into several groups with five, three and two clades, respectively. In addition, the distribution of nsLTP members within clades was not always quantitatively homogeneous between species, with some clades containing sequences only from wheat or *A*. *thaliana*. These species-specific clades, such as Type 2 [0-0-9], [0-0-16] and [0-0-125] and type D [5-0-0] and [0-0-13], contained nsLTPs with proline-rich sequences at the N-terminal of the 8 CM.

### Gene and protein structures of the TaLTPs

Potential protein post-translational modifications of the 461 identified TaLTPs were investigated, including predictions of signal peptide domains and pre-GPI anchor transmembrane domains^16,17^. Following cleavage of the predicted signal peptide and pre-GPI anchor domain, 418 unique mature proteins remained; among these, 386 TaLTPs possessed a unique 8 CM.

nsLTPs are characterized by the highly conserved 8 CM and analysis of the 8 CM consensus within wheat sub-types identified a variable number of inter-cysteine amino acid residues (Table 2). Type 2 TaLTPs contained the most variable spacing across the 8 CM, a likely consequence of the large number of members relative to the other sub-types. All Type C TaLTPs possessed a spacing of 12 residues between the Cys_6_ and Cys_7_ of the 8 CM, while a spacing of 12 residues was not present in any other TaLTP class.

**Table 2:** Diversity of eight cysteine motifs of TaLTPs.

Type	№	Spacing pattern									
1	39	C	X_9_	C	X_13–15,19_	CC	X_19–20,21_	CXC	X_20–24_	C	X_7,13,14,15_
2	274	C	X_3,5,7,8–10,17_	C	X_11,12–19,21,27_	CC	X_8–13,19_	CXC	X_8,10,15,18,19,21–27,36_	C	X_3–9,11,13,15,16,17_
C	5	C	X_9_	C	X_14,19_	CC	X_9_	CXC	X_12_	C	X_6_
D	57	C	X_9,10,12–13–14_	C	X_14,16–18_	CC	X_9–12_	CXC	X_11,17,23,24,27_	C	X_4,7,9,10,11,20_
G	86	C	X_6,9,10,12_	C	X_8,13–18,20_	CC	X_12,14,19_	CXC	X_21–26,29_	C	X_5,6,8–10,13,20_

“X” represents any amino number, and the number following “X” indicates the number of amino acid residues.

Underlined numbers indicate spacings specific for a type.

When analysing the 8 CM spacing across all TaLTPs, we also identified conservation in the amino acids within these spaces, reflecting higher identity within, but not across sub-types. This was depicted using WebLogo3 tool^18^ (Supplementary Fig. S2). For the CXC motif, hydrophobic residues at the X position were observed for most type 2 (86.1%) and type G (87.0%) and type C (100%) proteins, whereas the presence of a hydrophilic residue was predominantly observed in type 1 TaLTPs (69.2%).

To better understand TaLTP protein characteristics, we analysed the isoelectric point (pI) and molecular weight (MW) for all putative TaLTPs. Their MW ranged from 6.73 kDa to 21.73 kDa. Type G nsLTPs have previously been reported to possess the highest MW due to the presence of supernumerary amino acid residues C-terminal to the 8 CM. In contrast, to which has been reported in other species (*Zea mays*, *Marchantia polymorpa*, *Physcomitrella patens*, *Selaginella moellendorffi*, *Adiantum capillus-veneris*)^6,7^, five type D TaLTPs had a higher MW than type G TaLTPs. Furthermore, type 2 nsLTPs previously reported to be 7 kDa proteins, averaged 10.11 kDa in wheat^5^.

Exon-intron structure was also used to classify wheat *nsLTPs*, based on criteria proposed by Edstam *et al*.^9^. Accordingly, type 2 *TaLTPs* were lacking introns, with the exception of 11 genes containing an intron downstream of the 8 CM containing exon and classified as type 2 proteins based on peptide sequence identity (*TaLTP2*.*71*, *TaLTP2*.*82*, *TaLTP2*.*115*, *TaLTP2*.*127*, *TaLTP132*, *TaLTP2*.*135*, *TaLTP2*.*173*, *TaLTP2*.*217*, *TaLTP2*.*218*, *TaLTP2*.*220* and *TaLTP2*.*238*) (Supplementary Fig. S3). All type 1, type C and type D genes contained one intron positioned respectively at nucleotide 5, 1 and 4 after the last cysteine of the 8 CM. In contrast, Type G *TaLTPs* contained up to four introns.

### Chromosomal localization and duplication of TaLTPs gene family members

**Figure 2:**
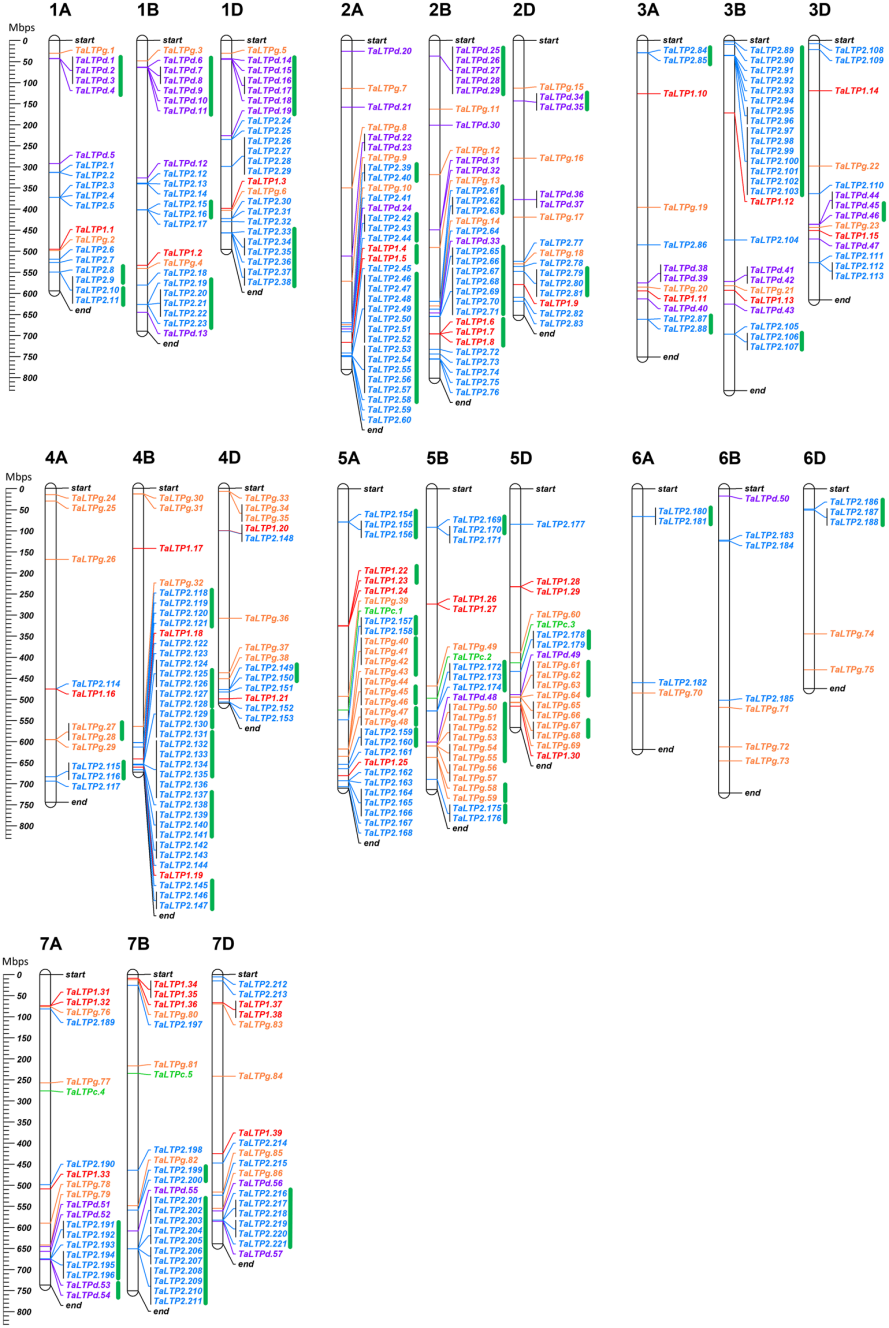
Chromosomal location of *TaLTPs*. The scale represents megabases (Mbps). Chromosome names are indicated above each vertical bar. Green bars indicate clusters of tandemly duplicated genes.

Of the 461 *TaLTPs*, physical location for 408 *TaLTPs* was identified within the Chinese Spring reference sequence (IWGSC RefSeq v1.0)^19^ (Fig. 2). *TaLTPs* were unevenly distributed across the 21 wheat pseudo-molecules. Chromosome 4B contained the highest number of *TaLTPs* (36), while fewest (4) were identified on chromosome 6A. In addition, *TaLTPs* distribution varied across the A (129 *nsLTPs*; 1 *TaLTP*/38.3 Mbps), B (169 *nsLTPs*, 1 *TaLTP*/30.7 Mbps) and D (110 *nsLTPs*; 1 *TaLTP*/35.9 Mbps) sub-genomes.

This uneven density of *TaLTPs* between sub-genomes is a likely consequence of ancestral translocation and duplication events. One such example is the presence of two significant clusters of tandem repeat type 2 *TaLTPs* found on both chromosome 3BS and 4BL relative to their homeologues. We identified 54 tandem duplication clusters involving a total of 200 *TaLTPs*. This suggests that some *TaLTPs* have undergone more than one round of duplication. Among the 200 duplicated genes, 144 belong to type 2 *TaLTPs*. This may explain why type 2 is found to be over-represented in the wheat genome relative to other species.

To analyse the evolutionary processes of type 2 *TaLTPs*, we performed non-synonymous (Ka) and synonymous (Ks) substitution ratio analysis of the three largest *TaLTP* duplication clusters (Chr2A, TaLTP2.46-TaLTP.58; Chr3B, TaLTP2.91-TaLTP2.103 and Chr7B, TaLTP2.201-TaLTP2.211) (Supplemental Table S2; Fig. 2). It was found that most duplicated pairs had a ratio Ka/Ks lower than one, implying that the genes evolved under influence of purifying selection with limited functional divergence after duplication. The Ka/Ks of TaLTP2.54/TaLTP2.49 gene pair was 1.0805, indicating a neutral selection pressure in evolution. Furthermore, divergence time of the duplicated genes were estimated by based on the number of non-synonymous substitution (Ks). Out of the 31 duplicated pairs analysed the Ks values were null for 38.7% of them, indicating recent duplication events. For the remaining duplicated pairs, their corresponding duplication age were estimated to vary from 0.73 million years ago (MYA) to 53.9 MYA.

### Expression analysis of *TaLTPs*

**Figure 3:**
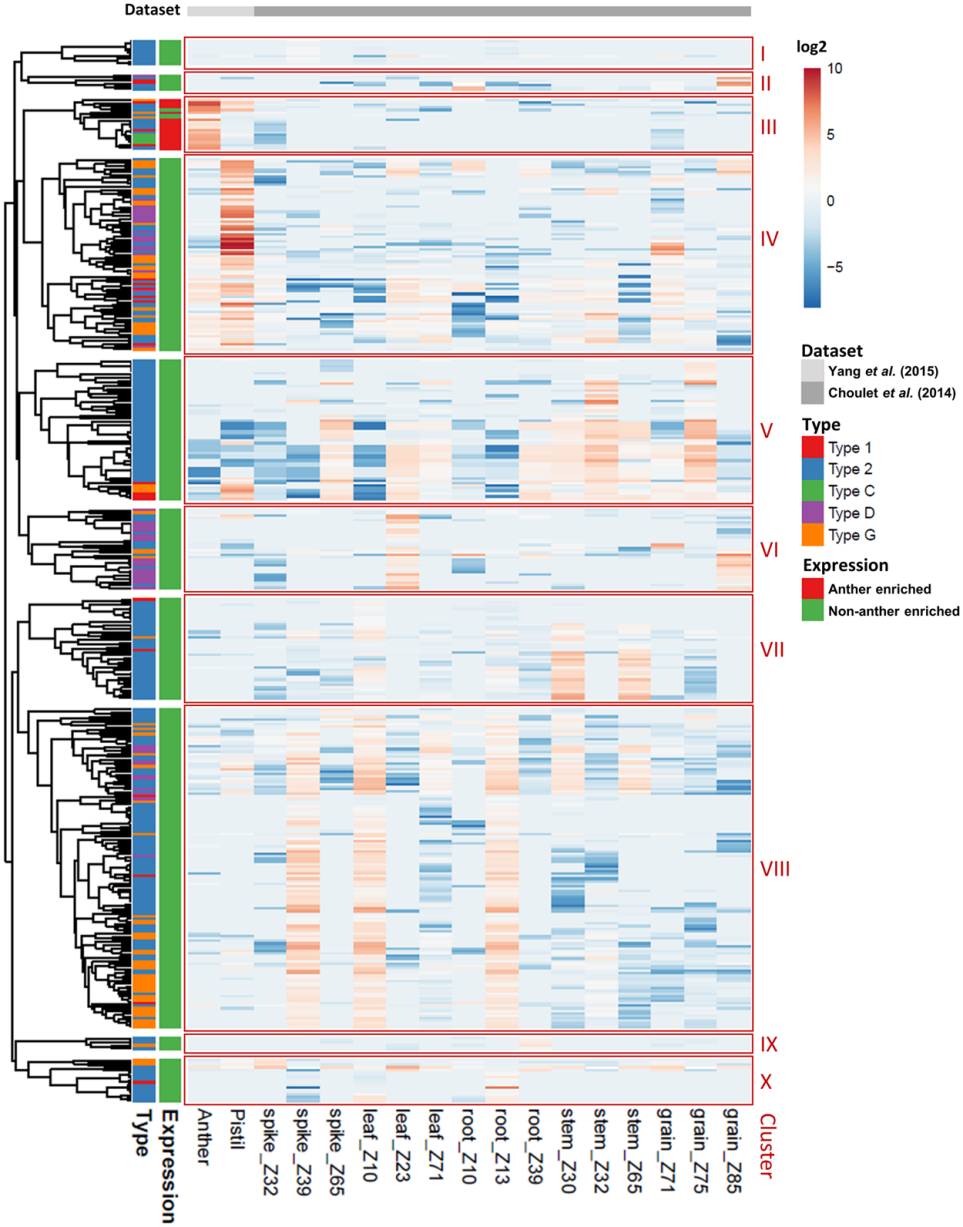
Heat map representation and hierarchical clustering of transcript level of *TaLTPs* across different tissues. Data were retrieved from Choulet *et al*. (2014) and Yang *et al*. (2015)^70,71^. Genes were clustered by an average-linkage method (Metsalu, 2005). The transcript abundance in fragments per kilobase of exon per million fragments mapped (FPKM) of each genes is available as Supplementary Table S3.

The expression profiles of all 461 predicted *TaLTPs* were first analysed using public RNA-seq datasets from seven different tissues including leaves, roots, grains, stems, spikes, pistils and anthers (Fig. 3, Supplementary Table S3). Their expression patterns varied greatly across tissues and developmental stages. No expression was detected for 30 *TaLTPs*, indicating that they are either pseudogenes or expressed in tissues or under specific environmental conditions for which RNA-seq data was not available.

Based on expression profiles, *TaLTPs* were divided into ten clusters (I to X) by hierarchical ordering (i.e. Cluster III contains anther-enriched genes while Cluster IV mostly contains genes with highly expressed in pistils). Type 1, type 2 and type G *TaLTPs* were present in most clusters, reflecting ubiquitous expression across tissues and developmental stages. In contrast, type C *TaLTPs*, only present in Cluster III, are found preferentially expressed in anthers. Comparing the proportion of *TaLTPs* per type and the proportion of TaLTP types per cluster found no significant distribution differences within Cluster VIII. In contrast, Cluster V (32 members) contained genes with expression enriched in leaf (Z23) and grain (Z85), and accounted for 56.2% of type D *TaLTPs* while overall, type D *TaLTPs* comprise only 12.4% of total *TaLTPs*.

In order to identify genes potentially involved in pollen development, we focussed on *TaLTPs* genes preferentially expressed in anthers. In total, 17 *TaLTPs* showed an anther-enriched expression profile based on the RNA-seq data. Among these, only *TaMs1*, a type G *nsLTP*, has been reported as anther-specific, and demonstrated to be involved in pollen exine development^13,14^. Two of the loci containing putative anther-expressed genes were found to possess only two *nsLTPs* homeologues from the three sub-genomes (TaLTPc4/TaLTPc.5 and TaLTPg.19/TaLTPg.22).

**Figure 4:**
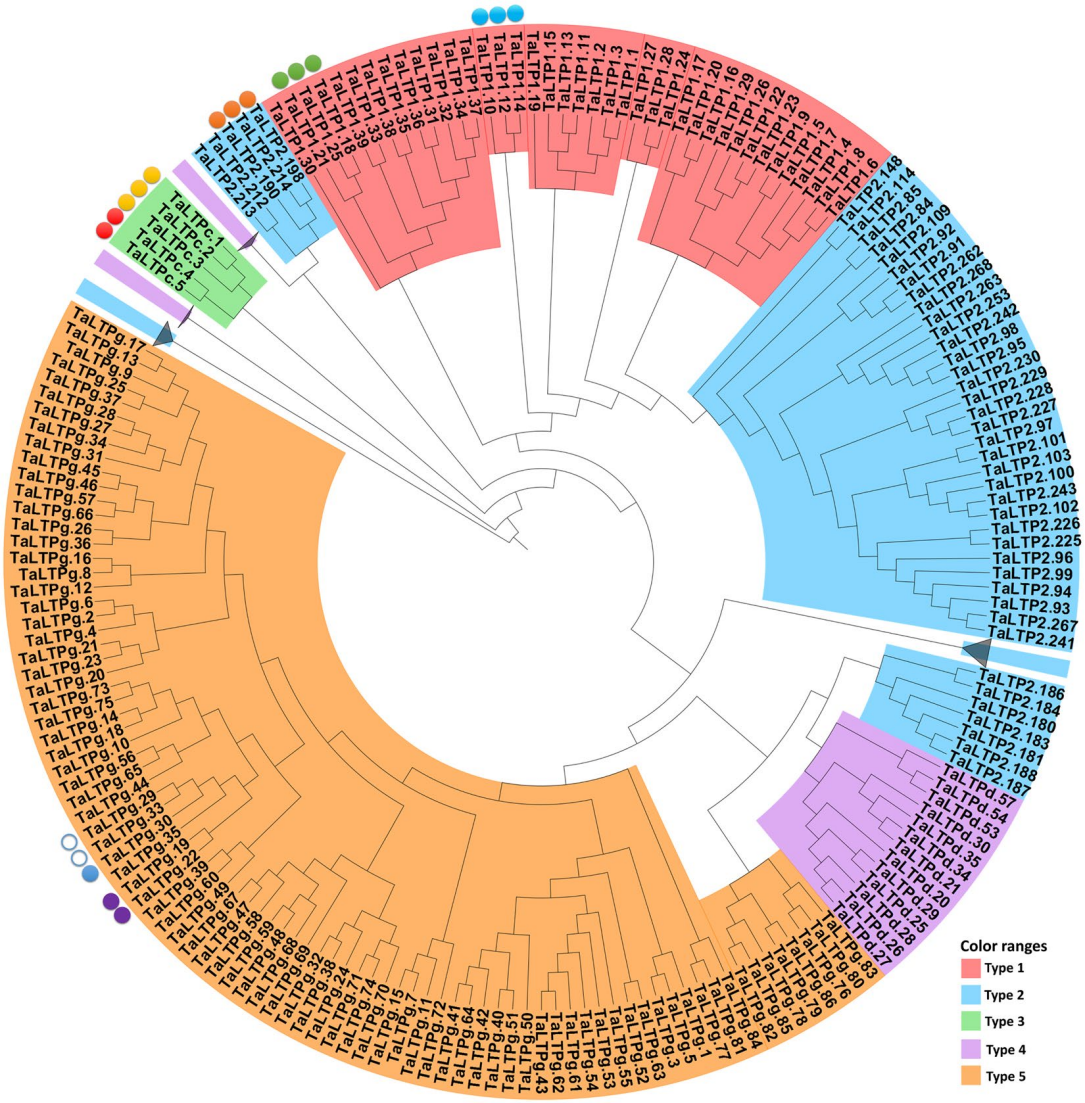
Unrooted phylogenetic tree of *TaLTPs* highlighting potential anther-enriched genes. Filled circles represent anther-enriched genes identified by RNA-seq^70,71^, empty circles represent homeologous genes with no expression detected; different colors were used for different homeoloci. For more visibility, subtrees including sequences of the same type are grouped and represented by a grey triangle. The fully extended tree is available as Supplementary Fig. S4. The phylogenetic tree was built from an alignment of the predicted mature proteins.

The evolutionary relationship of all identified anther-expressed genes was demonstrated using phylogenetic analysis (Fig. 4). The selected *TaLTPs* were present within different clades, suggesting that these genes are derived from different ancestors. For the purpose of validating their anther-enriched expression profile, and to obtain more precise information on their expression timing during male gametogenesis, we conducted qRT-PCR across eight different wheat tissues including leaves, shoot, roots, glumes, lemmas, paleas, ovaries and anthers.

**Figure 5:**
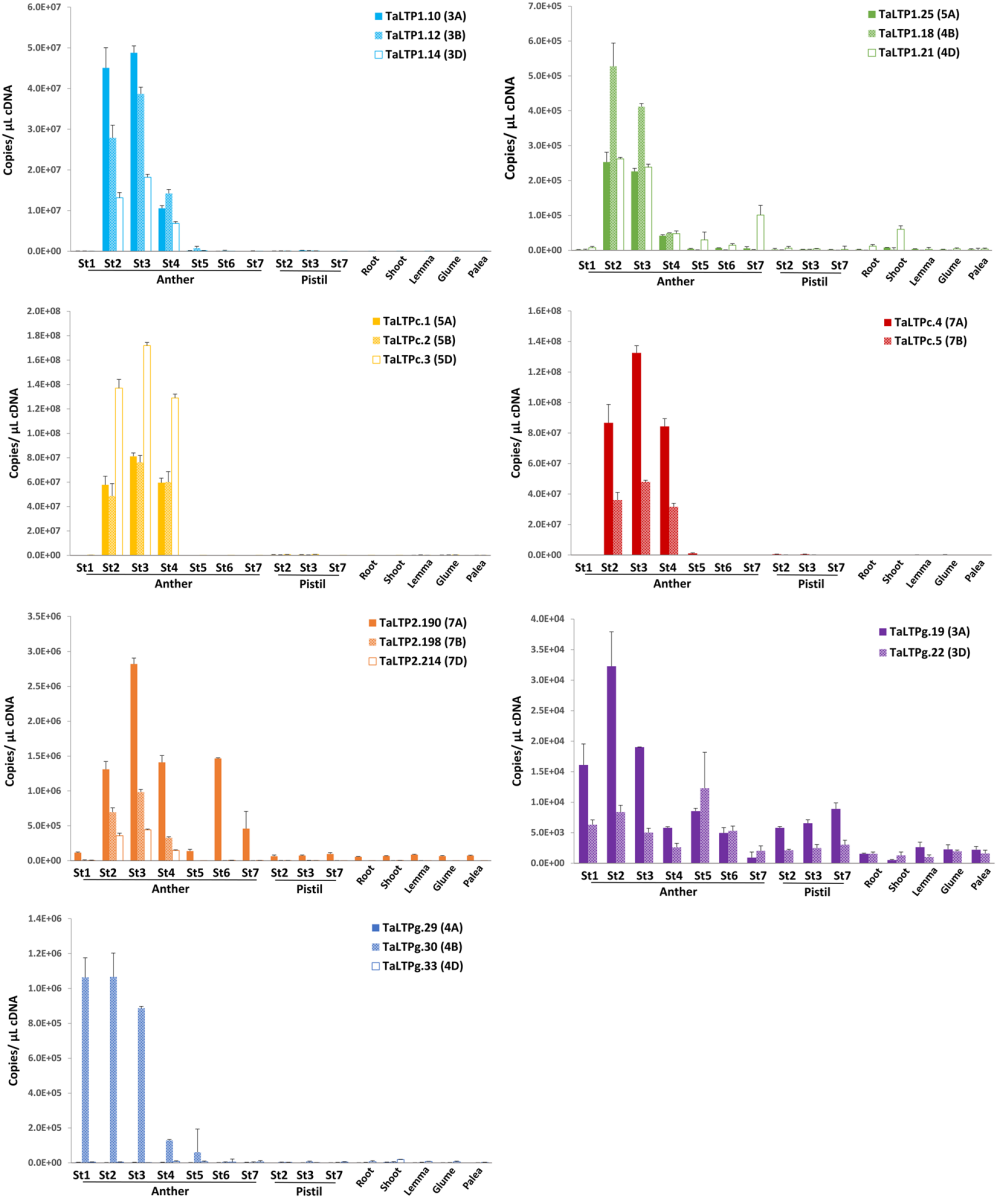
Expression levels of anther-enriched expressed TaLTP candidates. Each graph represents the expression profile for one homeologue group. St1, archesporial cells; St2, pre-meiotic pollen mother cells; St3, meiotic microspores; St4, early uninucleate; St5, late uninucleate; St6, binucleate; St7, mature pollen. Error bars reflect standard error of three independent tissue replicates (n = 3).

qRT-PCR results confirmed that the expression profiles for all selected genes were in agreement with the RNA-seq data (Fig. 5). These anther-enriched genes were highly up-regulated in anthers containing meiotic microspores, with the exception of TaLTPg.30 (*TaMs1*) which exhibited expression in anthers deemed to contain microspores at pre-meiosis. In addition, TaLTPg.30 was the only gene to show expression on only one sub-genome, whereas all other anther-enriched LTPs showed expression across two or all three sub-genomes.

### Promoter analysis of anther-specific *TaLTPs*

**Figure 6:**
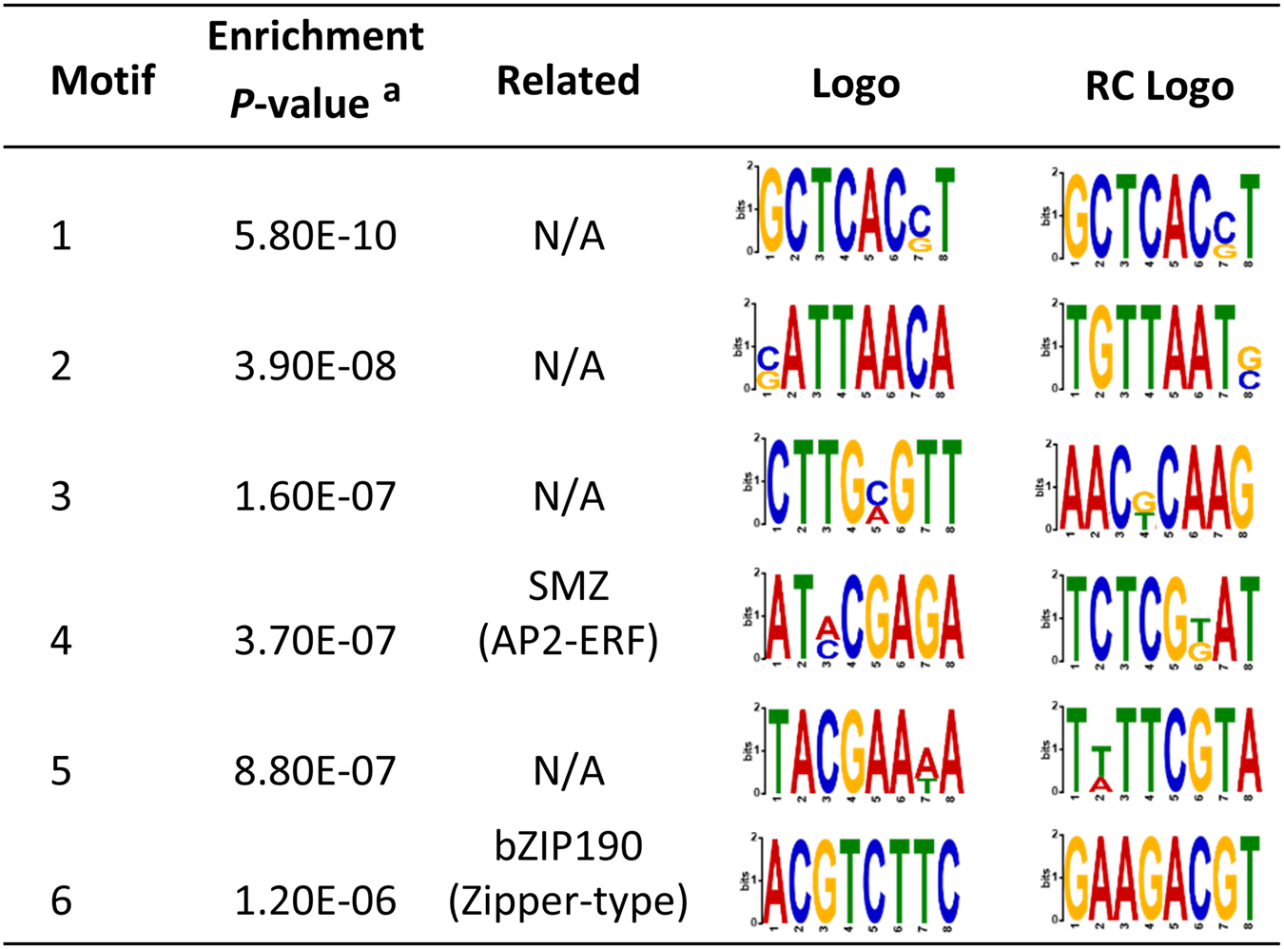
List of motifs enriched in anther-specific nsLTPs promoter regions. The enrichment *P*-value of the motif that are enriched in 17 anther-specific gene promoter regions compared with promoter sequences of the remaining 444 identified TaLTPs. Related, annotation of the motif; Logo, 5′−3′sequence; RC Logo, reverse-complement sequence; AP2-ERF, APETALA2-ethylene response factor; SMZ, SCHLAFMÜTZE.

To evaluate the presence of *cis*-elements within *TaLTP* promoter regions involved in anther-specific expression, we searched for over-represented motifs in anther-specific vs non-anther-specific sequences using MEME suite^20^. First, we focussed on nine boxes deemed to be associated with anther-enriched expression (Supplementary Table S4). Among these boxes, only the element POLLEN1LELAT52 (AGAAA) was identified to be enriched in anther-specific promoter regions (*P-*value = 6.72e^−3^)^21^. Secondly, we searched DNA motifs without *a priori* which were enriched in the anther-specific *TaLTPs* promoters relative to the remaining promoter sequence. A total of 6 motifs were identified to be significantly enriched in the anther-specific promoters (Fig. 6). Among these, two motifs were retrieved to be transcription factor binding sites (TFBSs); the TCTCGTAT motif (4), a putative binding site of APETALA2-ethylene response factor (AP2-ERF)^22^ and the ACGT core motif (6), a potential bZIP binding site^23^.

## Discussion

In the current study, a total of 461 *nsLTPs* were identified in the wheat genome (cv. Chinese Spring), including 39 type 1, 274 type 2, five type C, 57 type D and 86 type G. In comparison to *A*. *thaliana*, rice and maize, we found the wheat genome to contain over 5 times the number of *nsLTPs* (Table 1). The expansion of wheat *nsLTPs* could be due to the following reasons: (i) bread wheat is an allohexaploid species that originated from hybridization events involving three different diploid progenitors (AABBDD)^24^ (ii) small-scale gene duplication, including segmental and tandem duplication, may have played a significant role in *nsLTPs* gene family evolution in wheat. Genome duplication is generally accepted to be a primary source of genetic novelty through subfunctionalization and neofunctionalization and has been central to the evolution of angiosperms, leading to species divergence^25^. Duplication events of *nsLTPs* have been reported in various species, such as *A*. *thaliana*, turnip, rice, maize and cotton^7,8,26,27^. In this study, phylogenetic relationships between wheat, rice and *A*. *thaliana* nsLTPs showed an expansion of type 2 genes in wheat relative to rice and *A*. *thaliana* (Fig. 1). This is reflected by the presence of type 2 clades containing only wheat sequences, mostly present in tandem repeats on the wheat pseudomolecules (Fig. 2, Supplemental Table S2). We identified 54 tandem duplication clusters accounting for 200 *TaLTPs*, 72% of which were classified as type 2. Genic duplication and functional redundancy allows these gene sequences to accumulate mutations, increasing divergence and, over time, leading to expansion and evolution of the gene family^28^. (iii) Natural selection would favour duplication of genes that results in adaptive expansion of gene families, it is suggested that retention and expansion of resistance genes in bread wheat might be the results of selection during domestication^29^. Similar expansion was reported for the resistance *NBS-LRR* gene family in wheat^30^.

Phylogenetic relationships between *A*. *thaliana*, rice and wheat nsLTPs showed that some clades contained only wheat sequences (Fig. 1). These wheat specific clades were enriched in TaLTP sequences with high proline content at the N-terminus of the 8 CM. Here, we identify 142 proline rich TaLTPs (Supplemental Table S7). It is worth noting that nsLTPs from rice and Arabidopsis listed by Wei and Zhong (2014)^7^ did not incorporate such hybrid proline-rich proteins (HyPRPs) in their analysis. This could explain the presence of some wheat-specific *nsLTP* clades in our analysis. HyPRPs are suggested to be putative cell wall proteins^31,32^ that are typically responsive to multiple biotic and abiotic stress factors^33–35^. The hydrophobic 8 CM domain coupled to the hydrophilic proline-rich domain within the same polypeptide is indicative of a role for such proteins to function at the interface between the hydrophobic plasma membrane and the hydrophilic cell wall, and are reported to be involved in plant cell elongation^32^.

The observed expansion of *nsLTP* gene family in wheat relative to rice and the recent divergence time of the analysed *TaLTP* duplication clusters, which averaged 5.28 MYA (Supplemental Table S2), is in accordance with the divergence time of rice from *Triticeae* estimated of 50 MYA^36^. With the near complete genome assemblies of some *Triticeae* subgroups including, *Hordeum vulgare*, *Triticum urartu*, *Aegilops tauschii* and *T*. *turgidum ssp dicoccoides*^37–40^, new opportunities arise for a more thorough evolutionary analysis of the *nsLTP* gene family and further investigation of the duplication events that underpin the gene family expansion in wheat.

nsLTPs belong to a large family of pathogenesis-related proteins (PRPs), and are reported to play a role in defence against bacterial and fungal pathogens^41^. Some wheat nsLTPs have been shown to have an anti-fungal activity toward wheat and non-wheat fungal pathogens *in vitro* (TaLTP2.99, TaLTP2.241, TaLTP2.267, TaLTP2.227, TaLTP2.228, TaLTP2.229, TaLTP2.230, TaLTP2.242, TaLTP2.253, TaLTP2.263, TaLTP2.268, TaLTP2.109, TaLTP2.91, TaLTP2.92, TaLTP2.262, TaLTP1.22, TaLTP1.23)^42^. Surprisingly, no correlation was observed between their ability to inhibit pathogenic growth and lipid binding activity. However, it has been suggested that their toxicity could be derived from an alteration of the fungal membrane permeability^42^. Additionally, the wheat TaLTP1.22 (100% similarity with the tandem duplicate TaLTP1.23) was reported to be associated with resistance against *Fusarium graminearum*, and its transcript level was at least 50-fold more abundant in plants carrying the resistant allele Qfhs.ifa-5A^43^. Interestingly, these previously reported *TaLTP* genes involved in abiotic stress resistance from type 1 and type 2, were all retrieved within the same phylogenetic clades (Supplemental Fig. S4). In addition, these *TaLTPs* demonstrated a similar expression profile based on RNA-seq analysis, and grouped within the same cluster (V), showing high expression in stems (Z30, Z32 and Z65), leaves (Z23 and Z71) and grains (Z65). Therefore, we speculate that these TaLTPs may also possess antifungal activities. In rice, similar inhibition tests using LTP110 demonstrated a critical role for the residues Tyr17 and Arg46 and Pro72 in antifungal activity^44^. As expected, these residues were identified as highly conserved in type 1 TaLTPs (Tyr14 and Arg50 and Pro78) (Supplementary Fig. S2).

In diverse crops, the use of male sterility has been exploited for production of hybrid varieties that capture the benefits of hybrid vigour. It is reported that wheat hybrid vigour offers a yield gain of over 10% and improved yield stability^45^. One major limitation towards developing a commercially viable hybrid seed production platform in wheat was the lack of identification of a single locus genic male-sterile mutant and its associated wild-type restorer sequence^15,46^. This limitation has been overcome, only recently, by the identification the dominant fertility gene *TaMs1*, and the dominant sterility gene *Ms2*^13,14,47,48^. In addition, the recent development of novel gene-editing technologies opens opportunities to generate loss-of-function mutants in a single transgenic event in wheat^49^. *nsLTPs* represent potential candidates towards developing new male sterile mutants as studies have shown that defects in certain anther-expressed *nsLTPs* result in male sterility^10,11,13,50^. In order to identify *TaLTPs* responsible for ensuring male fertility, we analysed the expression profiles of selected *TaLTPs* using RNA-seq data and showed that most *TaLTPs* were expressed across a range of tissues and developmental stages. Interestingly, some members exhibited tissue-specific expression, suggesting important roles in physiological processes during wheat development. This included, 16 *TaLTP* genes (7 loci) which were preferentially or specifically expressed in anthers. These include, three type 2 (single locus), six type 1 (two loci), five type C (two loci) and 2 type G (two loci) genes. Orthologues for these wheat anther-expressed genes have been analysed in rice, maize and sorghum (Supplementary Table S5). Among the identified putative orthologs, only the maize type C *nsLTP (Ms44*) has been reported to be involved in male fertility^11^. In addition, no orthologues for *TaLTP2*.*190*, *TaLTP2*.*198* and *TaLTP2*.*214* could be retrieved for the studied species, suggesting a gain of function in wheat for male reproductive organ development. qRT-PCR analysis confirmed the expression profiles of these anther-expressed *TaLTPs* (Fig. 5). Among the anther-expressed *TaLTPs*, only *TaMs1* was identified as a single homeologue expressed gene.

To understand the promoter specificity of the identified anther-specific *TaLTPs* and to also identify putative *cis-*elements controlling their spatial and temporal expression, we identified enriched DNA motifs on promoter regions for the anther-specific *TaLTPs* relative to non-anther enriched *TaLTPs* (Supplementary Table S4, and Fig. 6). A total of seven motifs were identified including three previously reported elements. (i) The first is POLLENLELAT52 (AGAAA), an enhancer element of LAT52 deemed to be essential for high level of expression in pollen (Supplementary Table S4, and Fig. 6)^51^. (ii) The second is an AP2/ERF binding site element identified to regulate *SMZ*, involved in regulation of floral development in *A*. *thaliana*^22^. (iii) The third is a binding site element of bZIP190 (ACGT core), reported to be preferentially expressed in flowers of *Antirrhinum majus*^23^. An analysis by truncated transcriptional reporter fusions of the promoter would help to confirm functionality of these identified *cis-*elements in anther-enriched expression.

In conclusion, this is the first comprehensive and systematic analysis of nsLTPs in wheat. The structure, classification, evolution and expression profiles of 461 putative *TaLTPs* were analysed. The results of this study revealed the ubiquitous expression of *TaLTPs* during growth and development. The expansion of *TaLTPs* in the wheat genome was attributed to duplications during evolution. Our expression analysis may provide a solid basis for future studies of TaLTP function during wheat development. The identification of anther-expressed *TaLTPs* opens opportunities for the development of new male-sterile wheat lines for hybrid seed production.

## Materials and Methods

### Sequence retrieval and structural analysis

All nsLTP sequences of *Arabidopsis thaliana*, rice (*Oriza sativa*), sorghum (*Sorghum bicolor*) and maize (*Zea mays*) previously identified by Wei and Zhong (2014) were retrieved from phytozome^7,52^. To identify wheat putative nsLTPs we first used rice protein sequences (LOC_Os10g36070.1, LOC_Os07g18750.1, LOC_Os05g47700.1, LOC_Os05g40010.1, LOC_Os03g07100.1, LOC_Os01g68580.1, LOC_Os01g59870.1, LOC_Os01g12020.1) as queries to search against the Wheat IWGSC RefSeq v1.0^19^ using tBLASTn with an e-value of ≤10. Secondly, we examined hit sequences (+/*−*1000 bps) in all six DNA frames for presence of the 8 CM, and all hits lacking the essential Cys residues were excluded. Then, selected genomic sequences were used for gene prediction using TriAnnot and FGENESH programs with defaults parameters^53,54^. Subsequently, all predicted genes lacking the 8 CM were removed from further analysis. The remaining candidates were submitted to SUPERFAMILY 2 Beta tool, sequences annotated as proteinase/alpha-amylase inhibitor and seed storage family, 2S albumin were discarded (http://beta.supfam.org/). Additionally, putative nsLTPs lacking NSSs (examined with SignalP 4 server^55^) were also excluded. Presence of a C-terminal GPI-anchor signal was predicted using three prediction tools PredGPI, big PI-plant predictor and GPI-SOM^17,56,57^. Splice junctions were predicted using Splign Transcript to Genomic Alignment tool^58^. The nsLTP nomenclature was based on guidelines from Edstam *et al*.^6^.

### Phylogenetic analysis and gene duplications

*nsLTPs* sequences ID of rice (*Oriza sativa*), Sorghum (*Sorghum bicolor*), and Maize (*Zea Mays*) were retrieved from Wei and Zhong^7^. nsLTPs amino acid sequences were downloaded from ensembl plant^59^. All nsLTPs 8 CM sequences of the mature proteins were aligned using Clustal Omega^60^. After manually refinement of the multiple sequence alignment, the phylogenetic tree was built from alignment of the predicted mature proteins by Unweighted Pair Group Method with Arithmetic mean method using the Jalview program^61^. Trees were visualized using the iTOL web tool V4.0.3^62^.

### Gene structure analysis

In order to identify the precise splice-junction site of predicted *TaLTPs*, coding sequences (CDS) were aligned to genomic sequence using Splign alignment tool^58^. Schematic representation of *TaLTPs* gene structures was generated using the GSGS 2.0 tool^63^.

### Chromosomal mapping and duplication

*TaLTPs* were mapped onto the 21 wheat chromosomes according to their physical position (bp), from the short arm telomere to the long arm telomere based on IWGSC RefSeq V1.0^19^. MapChart was used to draw their location onto the physical map of each chromosome^64^. To detect gene duplications, the CDS sequences of *TaLTPs* in wheat were blasted against each other and selected with the following cut-offs: 80% of coverage with the similarity of the aligned regions above 80%. Tandemly duplicated *TaLTPs* were defined as two or more adjacent homologous genes located on a single chromosome.The estimation of the evolution rates of the duplicated *TaLTPs* was calculated using KaKs_calculator 2.0^65^. The divergence times (T) of *TaLTP* duplicated pairs were calculated as T = Ks/2r × 10^−6^ Mya, with a divergence rate (r) of 6.5 × 10^−9^ ^66^.

### Gene expression profiles based on RNA-seq

Previous wheat cv. Chinese Spring transcriptome examinations were performed by RNA-seq to investigate gene expression pattern of genes during vegetative and reproductive development. The transcript abundance of each gene was estimated by fragments per kilobase of exon per million fragments mapped (FPKM). Expression data of *TaLTPs* in spikes, leaves, roots, stems and grains, each sampled at three developmental stages, were retrieved from RNA-seq data downloaded from the European Nucleotide Archive database with the study number PRJEB5314 (http://www.ebi.ac.uk/ena/data/view/PRJEB5314). *TaLTPs* expressions data in pistils and anthers were downloaded from NCBI-SRA database with accession number SRP038912.

Heatmaps were generated from log2-transformed FPKM using the ClustVis web tool^67^.

### Quantitative RT-PCR analysis

Total RNA was isolated using ISOLATE II *RNA* Mini Kit (Bioline, Sydney, Australia) from wheat of cv. Chris tissues including roots, shoot apical meristem (SAM) and glume, lemma, palea, ovaries, and anthers containing microspores from pre-meiosis to maturity. Microspores were cytologically examined for stage of development. The remaining two anthers from the same floret were isolated and snap frozen in liquid nitrogen. All total RNA samples were treated with DNase I (Qiagen). 0.6 μg of RNA was used to synthesise the oligo (dT)-primed first strand cDNA using the superscript IV reverse transcriptase (Thermo Fisher, Adelaide, Australia). Quantitative real-time PCR was perform according to Burton *et al*.^68^ using the primer combinations shown in Supplemental Table S6. Amplification products from qRT-PCR on each tissue sample, three technical replicates and three biological replicates were used to estimate the transcript abundance of genes of interest relative to TaActin, TaGAPdH and Ta13-3-3 reference transcripts.

### Promoter analysis

For motif enrichment analysis using know *cis*-element deemed to be involved in pollen enriched expression (Supplementary Table S4) we used the AME web tool implemented in MEME suite^20^ with the following parameter: Ranksum enrichment test with a *P-*value threshold of 0.05.

For *de novo* motif discovery and enrichment, we used the DREME web tool implemented in MEME suite (version 4.12.0)^20^ with an e-value threshold of 0.05. MEME was also used to generate sequence logos for each discovered motif. Jaspar database was searched to find related transcription factor binding sites to the motifs identified using a relative profile score threshold of 80%^69^.

## Electronic supplementary material

Supplementary information

## Data Availability

The datasets generated during and/or analysed during the current study are available from the corresponding author on reasonable request.
