# Supplementary material for: Genome-wide identification and analysis of non-specific Lipid Transfer Proteins in hexaploid wheat
Source: Sci Rep. 2018 Nov 20;8:17087. doi: 10.1038/s41598-018-35375-7 (PMC6244205; doi:10.1038/s41598-018-35375-7)
Supplement: Supplementary file 1 — Supplementary information [file 41598_2018_35375_MOESM1_ESM.pdf]

Genome-wide identification and analysis of non-specific Lipid Transfer Proteins in hexaploid wheat

Allan Kouidri[1], Ryan Whitford[1], Radoslaw Suchecki[1,2], Elena Kalashyan[1], Ute Baumann[1]*

[1] University of Adelaide, School of Agriculture, Food & Wine, Waite Campus, Urrbrae, South Australia 5064, Australia.

[2] Current address Commonwealth Scientific and Industrial Research Organization, Agriculture and Food, Waite Campus, Urrbrae, South Australia 5064, Australia.

* Correspondence: ute.baumann@adelaide.edu.au

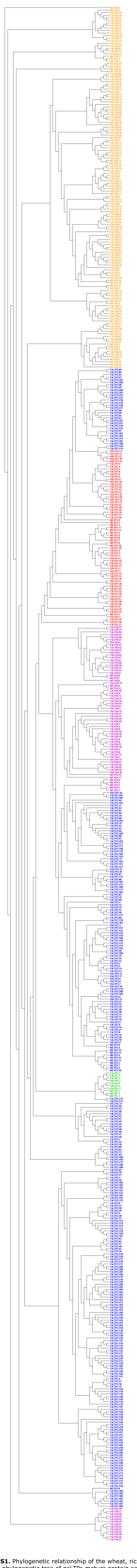

Supplementary Figure S1. Phylogenetic relationship of the wheat, rice and Arabidopsis nsLTP proteins. Unrooted phylogenetic tree of nsLTPs mature protein sequences from Wheat (TaLTP), Rice (OsLTP) and Arabidopsis (AtLTP). Red, Type 1; Blue, Type 2; Green, Type C; Purple, Type D; Type G, Orange.

All TaLTPs

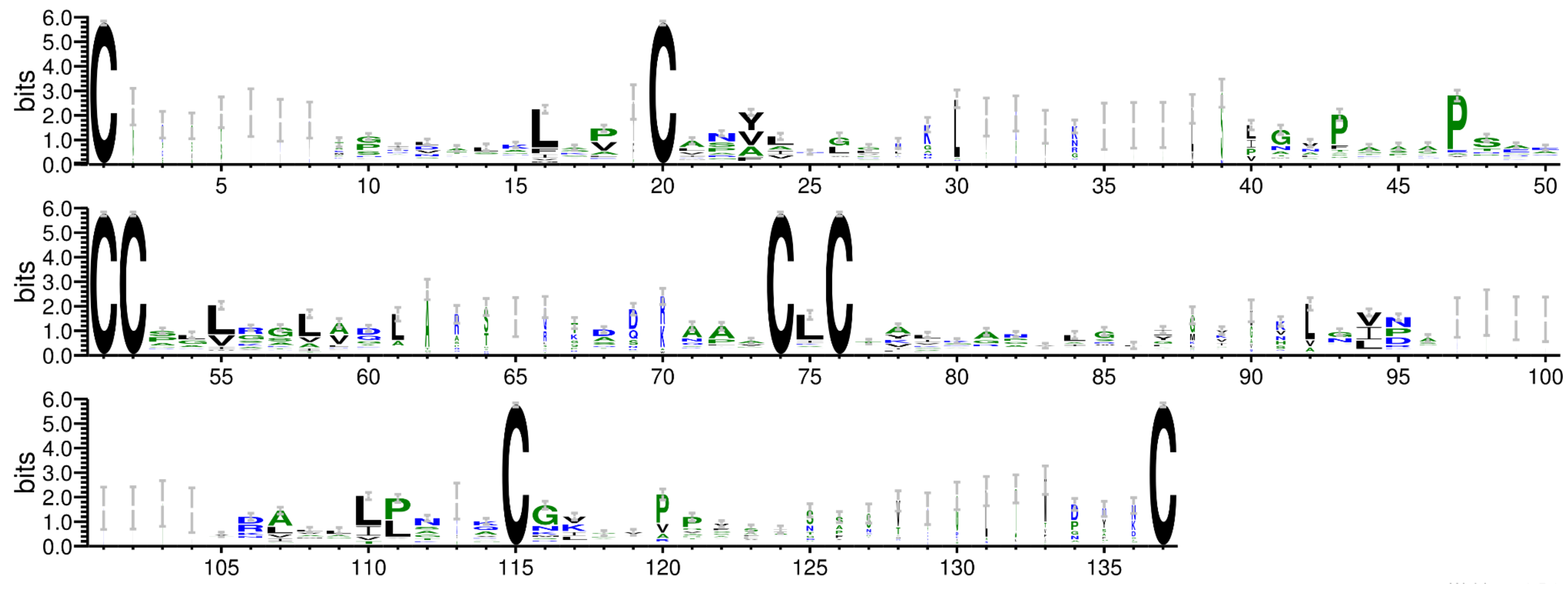

Type 1

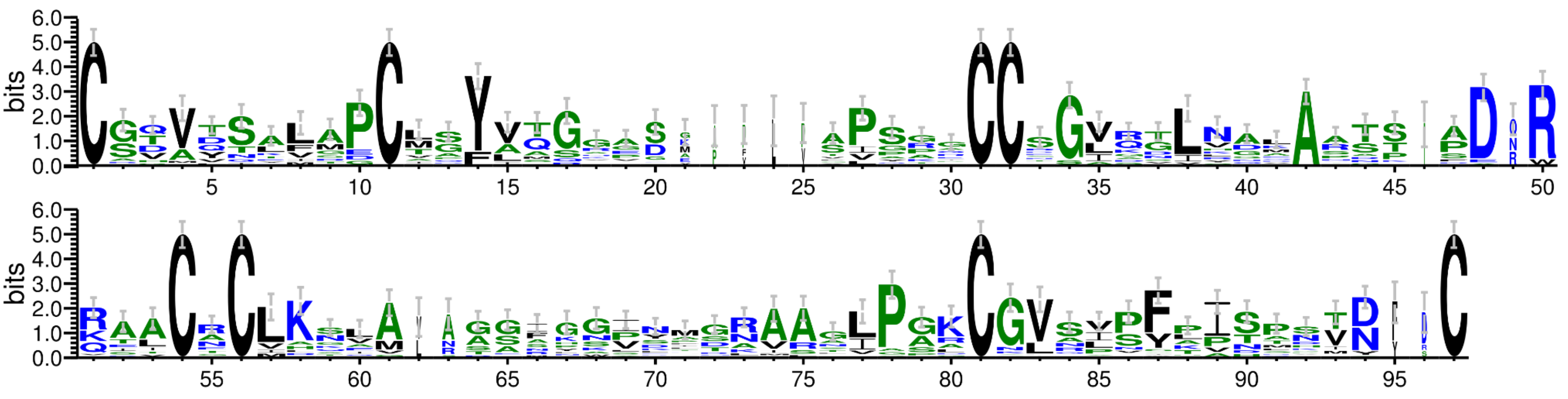

Type 2

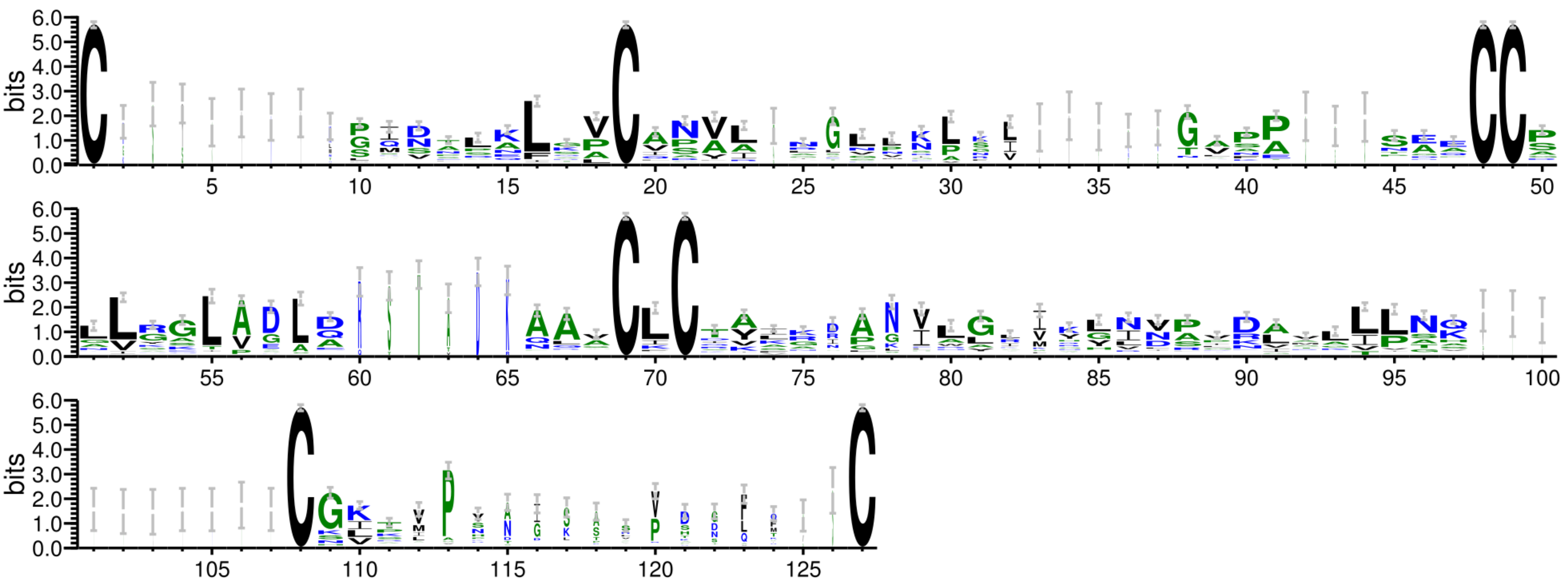

Type C

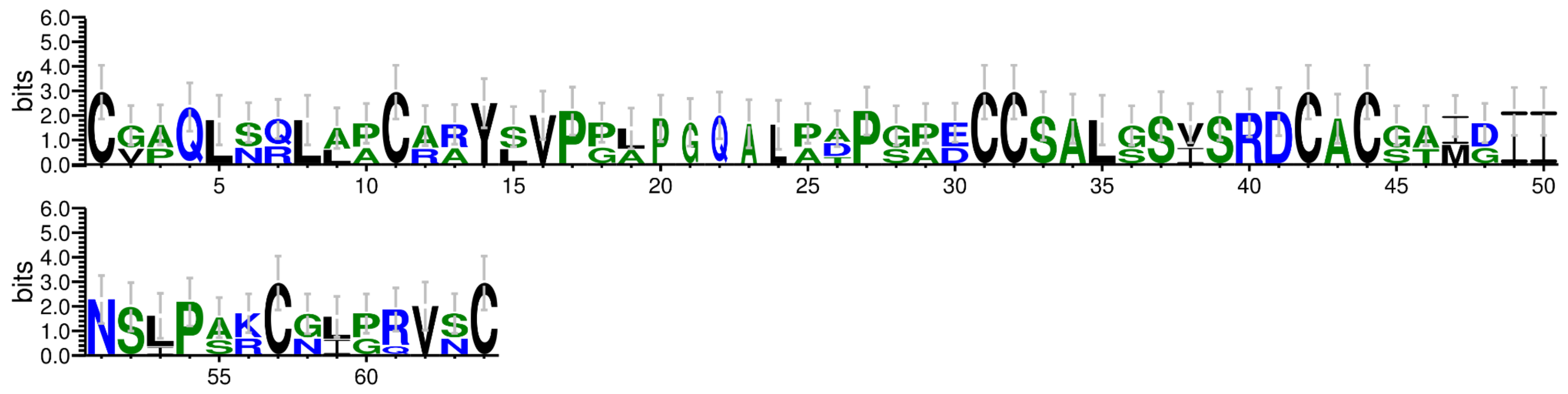

Type D

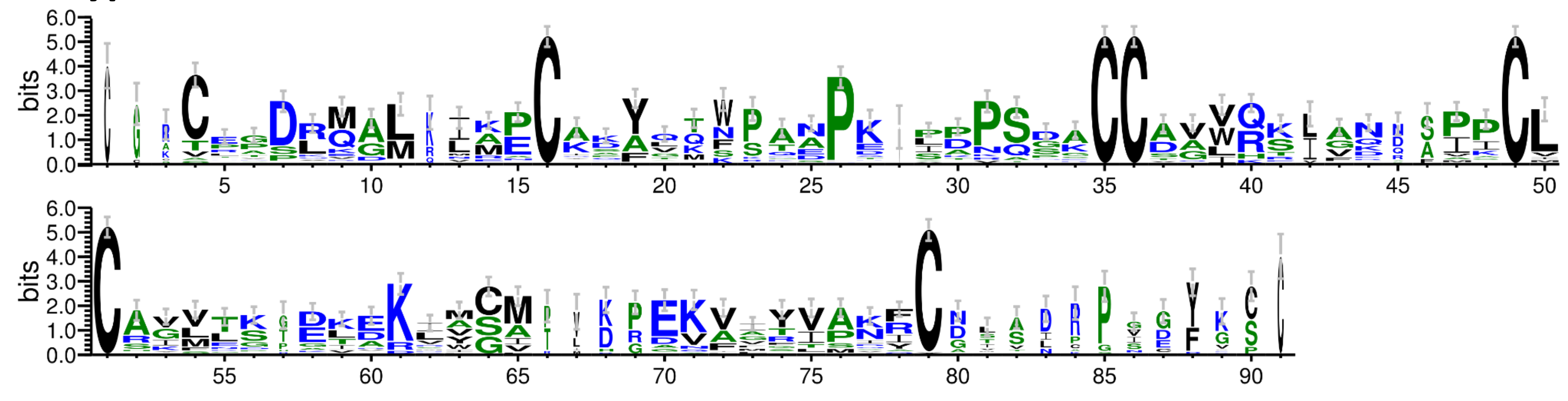

Type G

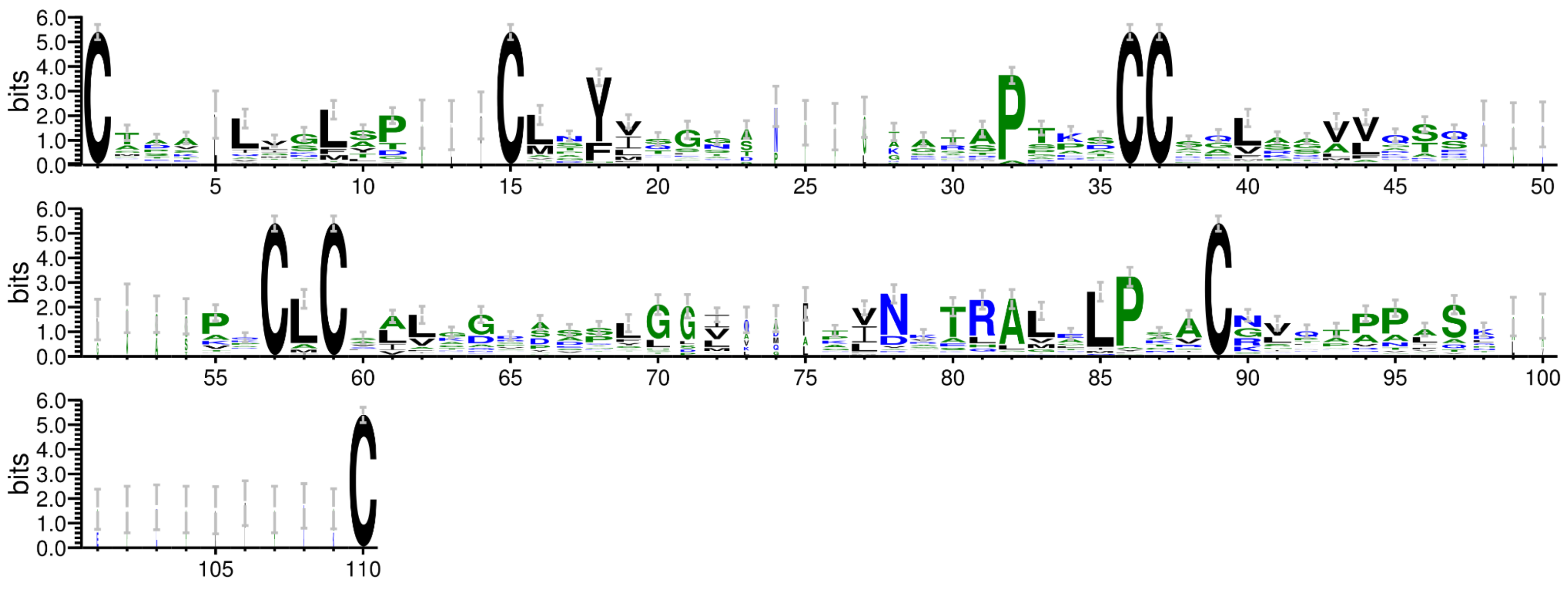

Supplementary Figure S2. Conserved domain analysis of the nsLTPs using the WebLogo3 program. The height of the letter designating the amino acid residue at each position represents the degree of conservation. The numbers on the x-axis represent the sequence positions in the corresponding conserved domains. The y-axis represents the information content measured in bits.

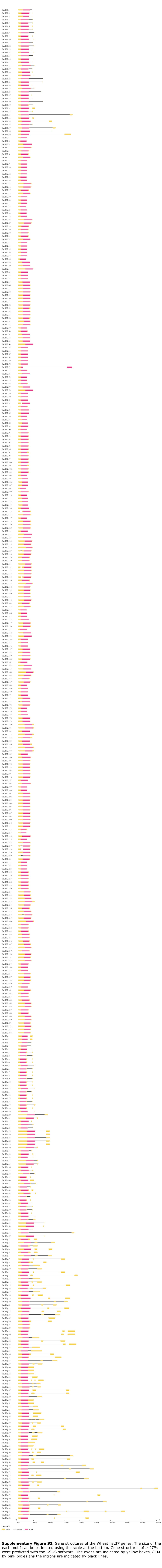

Supplementary Figure S3. Gene structures of the Wheat nsLTP genes. The size of the each motif can be estimated using the scale at the bottom. Gene structures of *nsLTPs* were predicted with the GSDS software. The exons are indicated by yellow boxes, 8CM by pink boxes ans the introns are indicated by black lines.

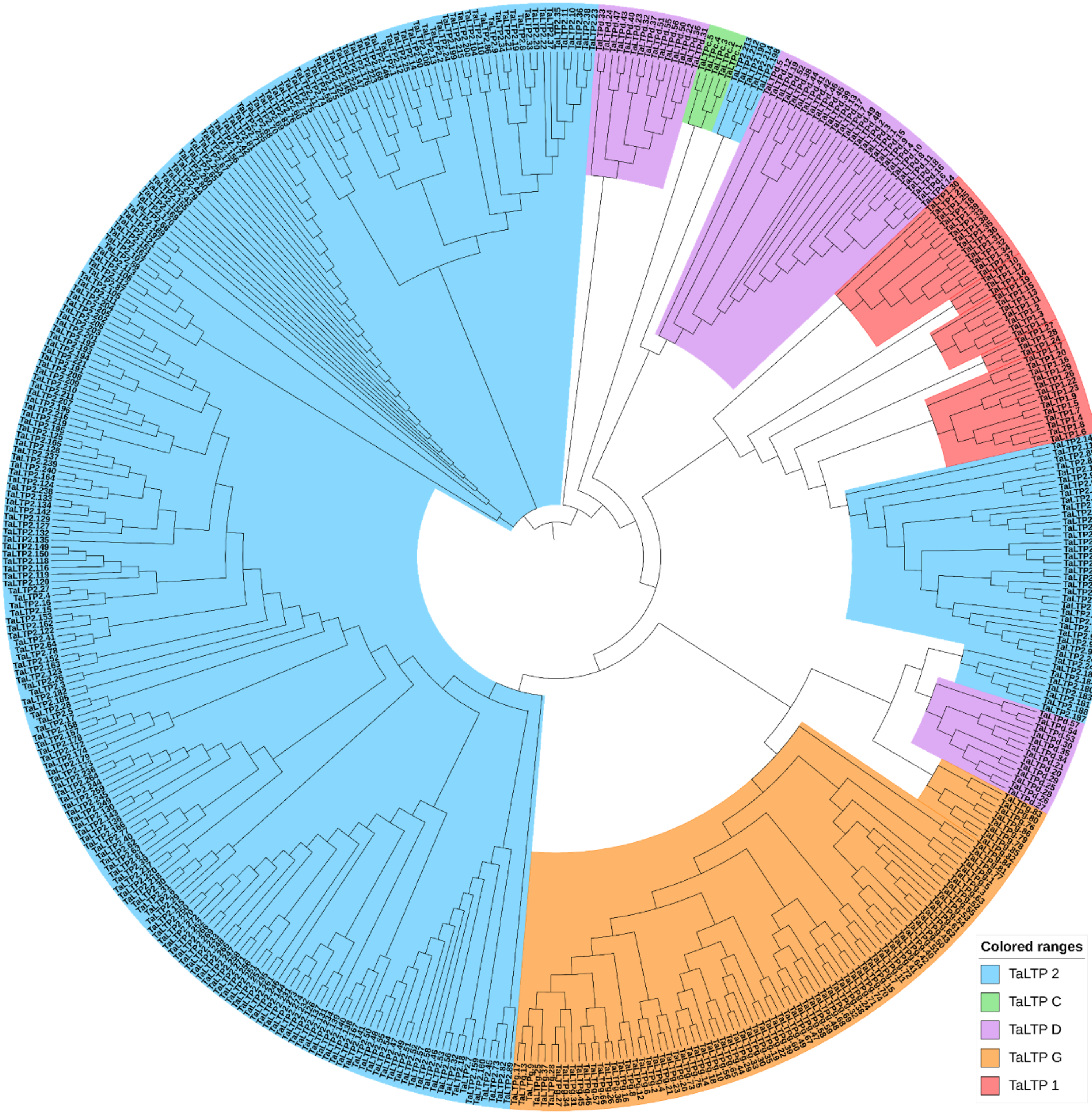

Supplementary Figure S4. Unrooted phylogenetic tree of the wheat nsLTPs gene family. The evolutionary distances were computed using the Poisson correction method (Zuckerkandl and Pauling, 1965). The analysis involved 461 amino acid sequences of the predicted mature proteins. All positions containing gaps and missing data were eliminated. There were a total of 24 positions in the final dataset. Evolutionary analyses were conducted in MEGA7 (Kumar *et al.,* 2017).

Supplementary Table S1. Physical positions of Non-specific Lipid Transfer Proteins in wheat Ref 1.0 assembly, and their protein features.

		Position				Signal peptide	Mature protein		
**Name**	**Chromosome**	**Start**	**End**	**Strand**	**8CM pattern[1]**	**AA[2]**	**AA**	**Mw[3]**	**pI[4]**
TaLTP1.1	chr1A	495284357	495284829	1	M-38-C-9-C-13-CC-19-C-1-C-23-C-13-C-3	37	92	9.31	10.96
TaLTP1.2	chr1B	533406182	533406642	1	M-35-C-9-C-13-CC-19-C-1-C-23-C-13-C-3	34	92	9.41	11.51
TaLTP1.3	chr1D	398044523	398048444	1	M-32-C-9-C-13-CC-19-C-1-C-23-C-13-C-3	31	92	9.31	10.96
TaLTP1.4	chr2A	716544323	716544799	-1	M-26-C-9-C-14-CC-19-C-1-C-20-C-13-C-4	20	96	9.43	9.99
TaLTP1.5	chr2A	716549208	716549949	-1	M-26-C-9-C-14-CC-19-C-1-C-20-C-13-C-4	25	91	8.98	10.64
TaLTP1.6	chr2B	695928027	695928501	-1	M-26-C-9-C-14-CC-19-C-1-C-22-C-13-C-4	20	98	9.73	9.99
TaLTP1.7	chr2B	695992511	695992985	-1	M-26-C-9-C-14-CC-19-C-1-C-22-C-13-C-4	25	93	9.23	10.43
TaLTP1.8	chr2B	696080662	696081190	-1	M-26-C-9-C-14-CC-19-C-1-C-22-C-13-C-4	20	98	9.68	10.28
TaLTP1.9	chr2D	579060097	579065485	-1	M-26-C-9-C-14-CC-19-C-1-C-20-C-13-C-4	25	91	8.90	10.38
TaLTP1.10	chr3A	126035274	126035793	1	M-29-C-9-C-15-CC-19-C-1-C-23-C-13-C-7	28	98	9.86	4.36
TaLTP1.11	chr3A	594349103	594349584	-1	M-29-C-9-C-13-CC-19-C-1-C-23-C-13-C-4	29	92	9.56	9.87
TaLTP1.12	chr3B	171701364	171701884	1	M-29-C-9-C-15-CC-19-C-1-C-22-C-13-C-7	28	97	9.80	4.36
TaLTP1.13	chr3B	592867387	592867840	-1	M-29-C-9-C-13-CC-19-C-1-C-23-C-13-C-4	29	92	9.56	9.87
TaLTP1.14	chr3D	119404001	119404521	1	M-29-C-9-C-15-CC-19-C-1-C-22-C-13-C-7	28	97	9.84	4.36
TaLTP1.15	chr3D	451234522	451235003	-1	M-30-C-9-C-13-CC-19-C-1-C-23-C-13-C-4	29	93	9.63	9.87
TaLTP1.16	chr4A	476145519	476146036	-1	M-33-C-9-C-14-CC-19-C-1-C-22-C-13-C-4	31	94	9.32	10.45
TaLTP1.17	chr4B	142541428	142541912	1	M-33-C-9-C-14-CC-19-C-1-C-22-C-13-C-4	31	94	9.39	10.44
TaLTP1.18	chr4B	642168978	642169391	1	M-43-C-9-C-19-CC-20-C-1-C-21-C-13-C-2	32	106	10.67	6.52
TaLTP1.19	chr4B	661641797	661642258	1	M-31-C-9-C-13-CC-19-C-1-C-23-C-7-C-4	30	87	9.06	11.43
TaLTP1.20	chr4D	100319858	100320221	1	M-33-C-9-C-14-CC-19-C-1-C-22-C-13-C-20	31	110	11.22	9.01
TaLTP1.21	chr4D	499312458	499312937	1	M-40-C-9-C-19-CC-20-C-1-C-21-C-13-C-27	31	129	13.50	6.64
TaLTP1.22	chr5A	324403095	324403903	-1	M-31-C-9-C-14-CC-19-C-1-C-22-C-13-C-4	30	93	9.48	10.45
TaLTP1.23	chr5A	324479786	324480589	-1	M-31-C-9-C-14-CC-19-C-1-C-22-C-13-C-4	30	93	9.48	10.45
TaLTP1.24	chr5A	325184136	325184588	-1	M-27-C-9-C-13-CC-19-C-1-C-22-C-13-C-3	27	90	9.67	9.23
TaLTP1.25	chr5A	680105615	680106025	1	M-42-C-9-C-19-CC-20-C-1-C-21-C-13-C-2	31	106	10.83	8.27
TaLTP1.26	chr5B	273147041	273147794	-1	M-31-C-9-C-14-CC-19-C-1-C-22-C-13-C-4	30	93	9.48	10.75

TaLTP1.27	chr5B	273458906	273459346	-1	M-27-C-9-C-13-CC-19-C-1-C-22-C-13-C-3	27	90	9.61	9.4
TaLTP1.28	chr5D	231788218	231788670	1	M-27-C-9-C-13-CC-19-C-1-C-22-C-13-C-3	27	90	9.48	7.1
TaLTP1.29	chr5D	232011275	232012084	1	M-30-C-9-C-14-CC-19-C-1-C-22-C-13-C-4	30	92	9.47	10.29
TaLTP1.30	chr5D	515641574	515642061	-1	M-33-C-9-C-15-CC-19-C-1-C-21-C-13-C-4	24	101	10.16	9.94
TaLTP1.31	chr7A	73746568	73747115	1	M-29-C-9-C-14-CC-19-C-1-C-24-C-14-C-32	25	127	13.24	4.32
TaLTP1.32	chr7A	74132167	74132646	1	M-24-C-9-C-14-CC-19-C-1-C-22-C-14-C-7	20	100	10.84	9.68
TaLTP1.33	chr7A	508650558	508650920	1	M-32-C-9-C-14-CC-19-C-1-C-21-C-13-C-34	29	124	12.94	4.07
TaLTP1.34	chr7B	8917078	8917597	-1	M-33-C-9-C-14-CC-19-C-1-C-22-C-14-C-22	29	115	12.60	9.22
TaLTP1.35	chr7B	8927933	8929029	-1	M-36-C-9-C-19-CC-21-C-1-C-24-C-15-C-33	32	136	14.52	9.39
TaLTP1.36	chr7B	8988470	8988937	-1	M-34-C-9-C-14-CC-19-C-1-C-22-C-14-C-4	30	97	9.86	3.91
TaLTP1.37	chr7D	66817216	66818437	-1	M-33-C-9-C-14-CC-19-C-1-C-22-C-14-C-35	29	128	13.90	9.69
TaLTP1.38	chr7D	66832500	66833605	-1	M-32-C-9-C-19-CC-21-C-1-C-24-C-15-C-4	25	110	12.10	5.8
TaLTP1.39	chr7D	424643201	424644915	-1	M-32-C-9-C-14-CC-19-C-1-C-21-C-13-C-71	29	161	16.86	4.49
TaLTP2.1	chr1A	313006783	313007070	-1	M-26-C-7-C-13-CC-8-C-1-C-23-C-6-C-2	26	70	7.14	9.6
TaLTP2.2	chr1A	313161875	313162150	-1	M-24-C-7-C-13-CC-8-C-1-C-23-C-6-C-0	25	67	7.06	11.17
TaLTP2.3	chr1A	372241197	372241652	-1	M-56-C-9-C-27-CC-13-C-1-C-25-C-9-C-2	33	119	11.59	8.91
TaLTP2.4	chr1A	372287159	372287593	-1	M-57-C-9-C-19-CC-13-C-1-C-24-C-3-C-9	21	124	12.68	8
TaLTP2.5	chr1A	372467568	372467930	-1	M-35-C-9-C-18-CC-13-C-1-C-24-C-3-C-8	20	101	10.58	7.77
TaLTP2.6	chr1A	518682557	518682901	-1	M-27-C-9-C-18-CC-13-C-1-C-24-C-3-C-10	22	93	9.83	8.13
TaLTP2.7	chr1A	526923822	526924223	1	M-49-C-10-C-15-CC-11-C-1-C-24-C-9-C-5	32	102	11.09	8.51
TaLTP2.8	chr1A	549637965	549638255	1	M-29-C-7-C-13-CC-8-C-1-C-23-C-6-C-0	29	68	7.09	9.78
TaLTP2.9	chr1A	549666495	549666785	1	M-29-C-7-C-13-CC-8-C-1-C-23-C-6-C-0	29	68	7.07	10.12
TaLTP2.10	chr1A	549670654	549670950	1	M-30-C-7-C-13-CC-9-C-1-C-23-C-6-C-0	30	69	7.19	10.58
TaLTP2.11	chr1A	549695442	549695735	1	M-29-C-7-C-13-CC-9-C-1-C-23-C-6-C-0	29	69	7.19	10.58
TaLTP2.12	chr1B	338430229	338430516	-1	M-26-C-7-C-13-CC-8-C-1-C-23-C-6-C-2	26	70	7.10	9.23
TaLTP2.13	chr1B	339364373	339364648	-1	M-24-C-7-C-13-CC-8-C-1-C-23-C-6-C-0	25	67	6.98	10.71
TaLTP2.14	chr1B	340367218	340367505	1	M-26-C-7-C-13-CC-8-C-1-C-23-C-6-C-2	26	70	7.11	8.3
TaLTP2.15	chr1B	401763150	401763584	-1	M-57-C-9-C-19-CC-13-C-1-C-24-C-3-C-9	21	124	12.71	9.03
TaLTP2.16	chr1B	401991935	401992366	-1	M-56-C-9-C-19-CC-13-C-1-C-24-C-3-C-9	21	123	12.61	9.03
TaLTP2.17	chr1B	402050747	402051103	-1	M-33-C-9-C-18-CC-13-C-1-C-24-C-3-C-8	20	99	10.41	7.77

TaLTP2.18	chr1B	580543508	580543909	1	M-49-C-10-C-15-CC-11-C-1-C-24-C-9-C-5	32	102	10.66	6.28
TaLTP2.19	chr1B	626506996	626507286	-1	M-29-C-7-C-13-CC-8-C-1-C-23-C-6-C-0	29	68	7.11	9.53
TaLTP2.20	chr1B	626595085	626595375	1	M-29-C-7-C-13-CC-8-C-1-C-23-C-6-C-0	29	68	7.11	9.78
TaLTP2.21	chr1B	626601013	626601303	1	M-29-C-7-C-13-CC-8-C-1-C-23-C-6-C-0	29	68	7.08	10.38
TaLTP2.22	chr1B	626610333	626610596	1	M-19-C-7-C-13-CC-9-C-1-C-23-C-6-C-0	19	69	7.27	10.86
TaLTP2.23	chr1B	626649118	626649411	1	M-29-C-7-C-13-CC-9-C-1-C-23-C-6-C-0	29	69	7.26	10.86
TaLTP2.24	chr1D	234528369	234528644	1	M-24-C-7-C-13-CC-8-C-1-C-23-C-6-C-0	25	67	6.99	11.2
TaLTP2.25	chr1D	234656387	234656674	1	M-26-C-7-C-13-CC-8-C-1-C-23-C-6-C-2	26	70	7.12	8.3
TaLTP2.26	chr1D	298573883	298574347	-1	M-59-C-9-C-27-CC-13-C-1-C-25-C-9-C-2	34	121	11.82	8.14
TaLTP2.27	chr1D	298603863	298604303	-1	M-59-C-9-C-19-CC-13-C-1-C-24-C-3-C-9	21	126	12.76	8
TaLTP2.28	chr1D	298619764	298620126	-1	M-35-C-9-C-18-CC-13-C-1-C-24-C-3-C-8	20	101	10.58	7.77
TaLTP2.29	chr1D	298635624	298635962	1	M-26-C-9-C-18-CC-13-C-1-C-24-C-3-C-9	22	91	9.73	7.14
TaLTP2.30	chr1D	422736634	422736978	-1	M-27-C-9-C-18-CC-13-C-1-C-24-C-3-C-10	22	93	9.97	8.15
TaLTP2.31	chr1D	422819751	422820095	-1	M-27-C-9-C-18-CC-13-C-1-C-24-C-3-C-10	22	93	9.94	8.14
TaLTP2.32	chr1D	429506893	429507294	1	M-49-C-10-C-15-CC-11-C-1-C-24-C-9-C-5	32	102	10.69	5.34
TaLTP2.33	chr1D	456132463	456132753	1	M-29-C-7-C-13-CC-8-C-1-C-23-C-6-C-0	29	68	7.12	9.78
TaLTP2.34	chr1D	456199792	456200082	1	M-29-C-7-C-13-CC-8-C-1-C-23-C-6-C-0	29	68	7.08	10.38
TaLTP2.35	chr1D	456203134	456203430	1	M-30-C-7-C-13-CC-9-C-1-C-23-C-6-C-0	30	69	7.27	10.86
TaLTP2.36	chr1D	456251729	456252025	1	M-30-C-7-C-13-CC-9-C-1-C-23-C-6-C-0	30	69	7.28	10.86
TaLTP2.37	chr1D	457033363	457033659	1	M-30-C-7-C-13-CC-9-C-1-C-23-C-6-C-0	30	69	7.26	10.86
TaLTP2.38	chr1D	457044170	457044433	1	M-19-C-7-C-13-CC-9-C-1-C-23-C-6-C-0	19	69	7.26	10.86
TaLTP2.39	chr2A	669567532	669567912	-1	M-42-C-9-C-18-CC-13-C-1-C-25-C-9-C-0	24	103	10.60	9.14
TaLTP2.40	chr2A	669619546	669619941	-1	M-47-C-9-C-18-CC-13-C-1-C-25-C-9-C-0	24	108	10.99	9.59
TaLTP2.41	chr2A	678831247	678831741	-1	M-78-C-9-C-19-CC-13-C-1-C-24-C-9-C-2	23	142	14.17	8.92
TaLTP2.42	chr2A	691116103	691116420	1	M-27-C-9-C-16-CC-9-C-1-C-26-C-7-C-1	28	78	8.20	9.78
TaLTP2.43	chr2A	691140275	691140592	1	M-27-C-9-C-16-CC-9-C-1-C-26-C-7-C-1	28	78	8.31	9.23
TaLTP2.44	chr2A	691169089	691169406	1	M-27-C-9-C-16-CC-9-C-1-C-26-C-7-C-1	28	78	8.33	8.32
TaLTP2.45	chr2A	741373075	741373470	1	M-46-C-9-C-18-CC-13-C-1-C-25-C-9-C-1	24	108	10.68	4.73
TaLTP2.46	chr2A	747697212	747697613	-1	M-49-C-10-C-15-CC-11-C-1-C-24-C-9-C-5	32	102	11.06	9.6
TaLTP2.47	chr2A	747723957	747724358	-1	M-49-C-10-C-15-CC-11-C-1-C-24-C-9-C-5	32	102	11.00	9.81

TaLTP2.48	chr2A	747727094	747727495	-1	M-49-C-10-C-15-CC-11-C-1-C-24-C-9-C-5	32	102	11.00	9.81
TaLTP2.49	chr2A	747730232	747730633	-1	M-49-C-10-C-15-CC-11-C-1-C-24-C-9-C-5	32	102	10.98	9.81
TaLTP2.50	chr2A	747733420	747733821	-1	M-49-C-10-C-15-CC-11-C-1-C-24-C-9-C-5	32	102	11.00	9.81
TaLTP2.51	chr2A	747739744	747740145	-1	M-49-C-10-C-15-CC-11-C-1-C-24-C-9-C-5	32	102	10.98	9.81
TaLTP2.52	chr2A	747742933	747743334	-1	M-49-C-10-C-15-CC-11-C-1-C-24-C-9-C-5	32	102	11.01	10.14
TaLTP2.53	chr2A	747773829	747774230	-1	M-49-C-10-C-15-CC-11-C-1-C-8-C-15-C-15	32	102	11.18	7.01
TaLTP2.54	chr2A	747777016	747777417	-1	M-49-C-10-C-15-CC-11-C-1-C-24-C-9-C-5	32	102	10.98	9.81
TaLTP2.55	chr2A	747780203	747780604	-1	M-49-C-10-C-15-CC-11-C-1-C-24-C-9-C-5	32	102	11.00	9.81
TaLTP2.56	chr2A	747809997	747810398	-1	M-49-C-10-C-15-CC-11-C-1-C-8-C-15-C-15	32	102	11.19	7.01
TaLTP2.57	chr2A	747819511	747819912	-1	M-60-C-3-C-11-CC-11-C-1-C-24-C-9-C-5	32	102	10.96	9.74
TaLTP2.58	chr2A	747874783	747875184	-1	M-49-C-10-C-15-CC-11-C-1-C-24-C-9-C-5	32	102	11.12	9.69
TaLTP2.59	chr2A	749626244	749626534	-1	M-26-C-7-C-13-CC-8-C-1-C-23-C-6-C-3	25	72	8.03	5.62
TaLTP2.60	chr2A	749855016	749855327	-1	M-26-C-9-C-15-CC-10-C-1-C-23-C-7-C-3	24	80	8.87	8.24
TaLTP2.61	chr2B	618873204	618873581	-1	M-41-C-9-C-18-CC-13-C-1-C-25-C-9-C-0	18	108	11.11	9.39
TaLTP2.62	chr2B	618968168	618968563	-1	M-47-C-9-C-18-CC-13-C-1-C-25-C-9-C-0	24	108	11.14	9.59
TaLTP2.63	chr2B	619044574	619044969	-1	M-47-C-9-C-18-CC-13-C-1-C-25-C-9-C-0	24	108	11.15	9.89
TaLTP2.64	chr2B	637444754	637445248	-1	M-78-C-9-C-19-CC-13-C-1-C-24-C-9-C-2	23	142	14.21	8.92
TaLTP2.65	chr2B	654545867	654546184	-1	M-27-C-9-C-16-CC-9-C-1-C-26-C-7-C-1	28	78	8.31	9.6
TaLTP2.66	chr2B	654588167	654588484	-1	M-27-C-9-C-16-CC-9-C-1-C-26-C-7-C-1	28	78	8.53	6.77
TaLTP2.67	chr2B	654648916	654649233	-1	M-27-C-9-C-16-CC-9-C-1-C-26-C-7-C-1	28	78	8.24	9.52
TaLTP2.68	chr2B	654652893	654653210	-1	M-27-C-9-C-16-CC-9-C-1-C-26-C-7-C-1	28	78	8.29	9.78
TaLTP2.69	chr2B	654681164	654681481	-1	M-27-C-9-C-16-CC-9-C-1-C-26-C-7-C-1	28	78	8.32	9.78
TaLTP2.70	chr2B	654702996	654703313	-1	M-27-C-9-C-16-CC-9-C-1-C-26-C-7-C-1	28	78	8.29	9.78
TaLTP2.71	chr2B	654730472	654732231	-1	M-27-C-9-C-16-CC-9-C-1-C-26-C-7-C-1	28	78	8.38	9.78
TaLTP2.72	chr2B	732839527	732839826	-1	M-26-C-7-C-13-CC-8-C-1-C-23-C-6-C-6	24	76	8.30	9.63
TaLTP2.73	chr2B	743695125	743695514	1	M-44-C-9-C-18-CC-13-C-1-C-25-C-9-C-1	26	104	10.41	4.94
TaLTP2.74	chr2B	755158846	755159136	-1	M-26-C-7-C-13-CC-8-C-1-C-23-C-6-C-3	25	72	7.95	8.29
TaLTP2.75	chr2B	755404195	755404482	-1	M-26-C-7-C-13-CC-8-C-1-C-23-C-5-C-3	25	71	8.15	5.73
TaLTP2.76	chr2B	756156764	756157078	-1	M-26-C-9-C-15-CC-10-C-1-C-23-C-7-C-4	22	83	9.22	6.09
TaLTP2.77	chr2D	524002804	524003199	-1	M-47-C-9-C-18-CC-13-C-1-C-25-C-9-C-0	24	108	11.08	9.59

TaLTP2.78	chr2D	535751756	535752250	-1	M-78-C-9-C-19-CC-13-C-1-C-24-C-9-C-2	23	142	14.23	9.16
TaLTP2.79	chr2D	547774709	547775026	-1	M-27-C-9-C-16-CC-9-C-1-C-26-C-7-C-1	28	78	8.25	9.31
TaLTP2.80	chr2D	547790937	547791254	-1	M-27-C-9-C-16-CC-9-C-1-C-26-C-7-C-1	28	78	8.25	9.52
TaLTP2.81	chr2D	547806114	547806431	-1	M-27-C-9-C-16-CC-9-C-1-C-26-C-7-C-1	28	78	8.32	9.78
TaLTP2.82	chr2D	609425079	609425471	1	M-31-C-9-C-18-CC-13-C-1-C-25-C-9-C-1	24	93	9.17	4.06
TaLTP2.83	chr2D	618868170	618868481	-1	M-26-C-9-C-15-CC-10-C-1-C-23-C-7-C-3	24	80	8.89	8.39
TaLTP2.84	chr3A	29048596	29048943	1	M-27-C-9-C-14-CC-19-C-1-C-19-C-13-C-4	26	90	8.94	10.67
TaLTP2.85	chr3A	29351629	29351976	-1	M-27-C-9-C-14-CC-19-C-1-C-19-C-13-C-4	26	90	8.95	10.67
TaLTP2.86	chr3A	484634127	484634414	1	M-28-C-7-C-13-CC-8-C-1-C-23-C-6-C-0	22	74	8.25	11.59
TaLTP2.87	chr3A	661655290	661655586	1	M-37-C-3-C-12-CC-9-C-1-C-21-C-6-C-0	25	74	7.71	9.51
TaLTP2.88	chr3A	661787323	661787640	1	M-44-C-3-C-12-CC-9-C-1-C-21-C-6-C-0	32	74	7.73	9.58
TaLTP2.89	chr3B	3092002	3092337	-1	M-26-C-9-C-18-CC-13-C-1-C-24-C-9-C-2	25	87	9.03	4.64
TaLTP2.90	chr3B	9427199	9427477	-1	M-25-C-7-C-13-CC-8-C-1-C-23-C-6-C-0	25	68	7.10	10.55
TaLTP2.91	chr3B	35037705	35038052	1	M-27-C-9-C-14-CC-19-C-1-C-19-C-13-C-4	26	90	8.77	10.52
TaLTP2.92	chr3B	35119171	35119518	1	M-27-C-9-C-14-CC-19-C-1-C-19-C-13-C-4	26	90	8.77	10.52
TaLTP2.93	chr3B	35212403	35212750	1	M-27-C-9-C-14-CC-19-C-1-C-19-C-13-C-4	26	90	8.66	10.75
TaLTP2.94	chr3B	35288587	35288934	1	M-27-C-9-C-14-CC-19-C-1-C-19-C-13-C-4	26	90	8.66	10.75
TaLTP2.95	chr3B	35697822	35698169	1	M-27-C-9-C-14-CC-19-C-1-C-19-C-13-C-4	26	90	8.73	10.75
TaLTP2.96	chr3B	35701446	35701793	1	M-27-C-9-C-14-CC-19-C-1-C-19-C-13-C-4	26	90	8.63	10.45
TaLTP2.97	chr3B	35754310	35754657	1	M-27-C-9-C-14-CC-19-C-1-C-10-C-8-C-18	26	90	8.72	10.46
TaLTP2.98	chr3B	35769759	35770106	1	M-27-C-9-C-14-CC-19-C-1-C-19-C-13-C-4	26	90	8.73	10.75
TaLTP2.99	chr3B	35773382	35773729	1	M-27-C-9-C-14-CC-19-C-1-C-19-C-13-C-4	26	90	8.66	10.75
TaLTP2.100	chr3B	35813194	35813541	1	M-27-C-9-C-14-CC-19-C-1-C-19-C-13-C-4	26	90	8.70	10.75
TaLTP2.101	chr3B	35823275	35823622	1	M-27-C-9-C-14-CC-19-C-1-C-10-C-8-C-18	26	90	8.70	10.46
TaLTP2.102	chr3B	35837851	35838198	1	M-27-C-9-C-14-CC-19-C-1-C-19-C-13-C-4	26	90	8.66	10.75
TaLTP2.103	chr3B	35839140	35839487	1	M-27-C-9-C-14-CC-19-C-1-C-19-C-13-C-4	26	90	8.72	10.75
TaLTP2.104	chr3B	473691569	473691859	1	M-29-C-7-C-13-CC-8-C-1-C-23-C-6-C-0	23	74	8.21	10.86
TaLTP2.105	chr3B	696672997	696673308	1	M-38-C-3-C-12-CC-9-C-1-C-23-C-8-C-0	22	82	8.61	11.5
TaLTP2.106	chr3B	696778128	696778445	1	M-44-C-3-C-12-CC-9-C-1-C-21-C-6-C-0	32	74	7.77	6.28
TaLTP2.107	chr3B	696788089	696788403	-1	M-43-C-3-C-12-CC-9-C-1-C-21-C-6-C-0	31	74	7.68	9.58

TaLTP2.108	chr3D	7430150	7430404	-1	M-17-C-7-C-13-CC-8-C-1-C-23-C-6-C-0	17	68	7.10	10.55
TaLTP2.109	chr3D	21328598	21328945	-1	M-27-C-9-C-14-CC-19-C-1-C-19-C-13-C-4	26	90	8.73	10.75
TaLTP2.110	chr3D	363593703	363593990	1	M-28-C-7-C-13-CC-8-C-1-C-23-C-6-C-0	22	74	8.26	11.24
TaLTP2.111	chr3D	526995531	526995842	1	M-38-C-3-C-12-CC-9-C-1-C-23-C-8-C-0	22	82	8.58	10.87
TaLTP2.112	chr3D	527126755	527127075	1	M-45-C-3-C-12-CC-9-C-1-C-21-C-6-C-0	33	74	7.67	8.11
TaLTP2.113	chr3D	527135956	527136279	1	M-46-C-3-C-12-CC-9-C-1-C-21-C-6-C-0	34	74	7.67	9.52
TaLTP2.114	chr4A	476142148	476142510	-1	M-30-C-9-C-13-CC-19-C-1-C-22-C-13-C-4	29	92	9.11	11.41
TaLTP2.115	chr4A	684448840	684449261	-1	M-28-C-9-C-19-CC-13-C-1-C-24-C-3-C-9	26	90	9.30	6.17
TaLTP2.116	chr4A	684471199	684471624	1	M-54-C-9-C-19-CC-13-C-1-C-24-C-3-C-9	23	119	12.33	6.37
TaLTP2.117	chr4A	694676879	694677172	1	M-26-C-7-C-13-CC-8-C-1-C-23-C-6-C-4	25	73	7.87	8.31
TaLTP2.118	chr4B	603025686	603026111	-1	M-54-C-9-C-19-CC-13-C-1-C-24-C-3-C-9	23	119	12.29	6.7
TaLTP2.119	chr4B	603144552	603144977	-1	M-54-C-9-C-19-CC-13-C-1-C-24-C-3-C-9	23	119	12.37	6.37
TaLTP2.120	chr4B	603148473	603148898	1	M-54-C-9-C-19-CC-13-C-1-C-24-C-3-C-9	23	119	12.31	5.89
TaLTP2.121	chr4B	613905713	613906024	1	M-27-C-9-C-17-CC-9-C-1-C-24-C-7-C-0	28	76	8.13	4.34
TaLTP2.122	chr4B	654440796	654441242	1	M-61-C-9-C-21-CC-13-C-1-C-24-C-9-C-1	25	124	12.11	8.92
TaLTP2.123	chr4B	654453330	654453749	-1	M-53-C-9-C-19-CC-13-C-1-C-24-C-9-C-2	23	117	11.35	6.9
TaLTP2.124	chr4B	654560940	654561404	-1	M-69-C-9-C-18-CC-13-C-1-C-24-C-3-C-8	23	132	13.11	4.93
TaLTP2.125	chr4B	654592596	654593027	-1	M-58-C-9-C-18-CC-13-C-1-C-24-C-3-C-8	23	121	12.04	4.92
TaLTP2.126	chr4B	654606592	654607062	-1	M-81-C-5-C-17-CC-13-C-1-C-19-C-3-C-8	26	131	13.47	4.44
TaLTP2.127	chr4B	654699101	654699538	-1	M-50-C-9-C-18-CC-13-C-1-C-24-C-3-C-8	23	113	11.61	4.92
TaLTP2.128	chr4B	654719678	654720109	-1	M-58-C-9-C-18-CC-13-C-1-C-24-C-3-C-8	23	121	12.08	4.92
TaLTP2.129	chr4B	654728109	654731861	-1	M-43-C-9-C-18-CC-13-C-1-C-24-C-3-C-8	24	123	11.04	9
TaLTP2.130	chr4B	654731472	654731861	-1	M-43-C-9-C-18-CC-13-C-1-C-25-C-3-C-8	24	106	10.98	9
TaLTP2.131	chr4B	654734070	654734513	-1	M-72-C-5-C-17-CC-13-C-1-C-19-C-3-C-8	26	122	12.57	4.44
TaLTP2.132	chr4B	654751596	654752033	-1	M-50-C-9-C-18-CC-13-C-1-C-24-C-3-C-8	23	113	11.41	4.86
TaLTP2.133	chr4B	654761835	654762272	-1	M-60-C-9-C-18-CC-13-C-1-C-24-C-3-C-8	23	123	12.37	4.93
TaLTP2.134	chr4B	654774041	654774478	-1	M-60-C-9-C-18-CC-13-C-1-C-24-C-3-C-8	23	123	12.38	4.93
TaLTP2.135	chr4B	654793453	654793885	-1	M-47-C-9-C-18-CC-13-C-1-C-24-C-3-C-8	23	110	11.27	7.85
TaLTP2.136	chr4B	654806594	654806983	-1	M-43-C-9-C-18-CC-13-C-1-C-25-C-3-C-8	24	106	11.00	7.76
TaLTP2.137	chr4B	654810532	654811005	-1	M-82-C-5-C-17-CC-13-C-1-C-19-C-3-C-8	26	132	13.47	4.11

TaLTP2.138	chr4B	654925077	654925481	-1	M-58-C-5-C-18-CC-13-C-1-C-19-C-3-C-8	27	108	11.04	4.92
TaLTP2.139	chr4B	654965422	654965826	-1	M-58-C-5-C-18-CC-13-C-1-C-19-C-3-C-8	27	108	11.10	4.63
TaLTP2.140	chr4B	655000545	655000949	-1	M-58-C-5-C-18-CC-13-C-1-C-19-C-3-C-8	27	108	11.07	4.93
TaLTP2.141	chr4B	655036289	655036693	-1	M-58-C-5-C-18-CC-13-C-1-C-19-C-3-C-8	27	108	11.11	4.63
TaLTP2.142	chr4B	655091831	655092268	-1	M-60-C-9-C-18-CC-13-C-1-C-24-C-3-C-8	19	127	12.73	6.94
TaLTP2.143	chr4B	655111018	655111407	-1	M-43-C-9-C-18-CC-13-C-1-C-25-C-3-C-8	24	106	11.05	8.62
TaLTP2.144	chr4B	656326666	656327079	1	M-62-C-5-C-17-CC-13-C-1-C-19-C-3-C-8	26	112	11.68	4.23
TaLTP2.145	chr4B	667648816	667649091	1	M-24-C-7-C-13-CC-8-C-1-C-23-C-6-C-0	25	67	6.96	9.52
TaLTP2.146	chr4B	667855279	667855569	-1	M-29-C-7-C-13-CC-8-C-1-C-23-C-6-C-0	30	67	6.98	9.4
TaLTP2.147	chr4B	667859023	667859307	-1	M-27-C-7-C-13-CC-8-C-1-C-23-C-6-C-0	28	67	7.09	7.07
TaLTP2.148	chr4D	100336137	100336499	1	M-30-C-9-C-13-CC-19-C-1-C-22-C-13-C-4	29	92	9.10	11.41
TaLTP2.149	chr4D	477543091	477543516	-1	M-54-C-9-C-19-CC-13-C-1-C-24-C-3-C-9	21	121	12.37	6.7
TaLTP2.150	chr4D	477559039	477559464	1	M-54-C-9-C-19-CC-13-C-1-C-24-C-3-C-9	23	119	12.16	6.37
TaLTP2.151	chr4D	483179528	483179824	-1	M-22-C-9-C-17-CC-9-C-1-C-24-C-7-C-0	23	76	8.13	4.34
TaLTP2.152	chr4D	507124730	507125155	1	M-56-C-9-C-19-CC-13-C-1-C-24-C-9-C-1	23	119	11.38	8
TaLTP2.153	chr4D	507163625	507164068	-1	M-60-C-9-C-21-CC-13-C-1-C-24-C-9-C-1	26	122	11.89	9.37
TaLTP2.154	chr5A	78368993	78369310	1	M-27-C-9-C-16-CC-9-C-1-C-26-C-7-C-1	28	78	8.30	6.78
TaLTP2.155	chr5A	78474456	78474773	1	M-27-C-9-C-16-CC-9-C-1-C-26-C-7-C-1	22	84	8.90	6.07
TaLTP2.156	chr5A	78480627	78480944	1	M-27-C-9-C-16-CC-9-C-1-C-26-C-7-C-1	28	78	8.29	9.87
TaLTP2.157	chr5A	547658439	547658837	1	M-46-C-9-C-18-CC-13-C-1-C-25-C-9-C-2	23	110	11.53	4.95
TaLTP2.158	chr5A	547711278	547711676	1	M-46-C-9-C-18-CC-13-C-1-C-25-C-9-C-2	23	110	11.51	6.76
TaLTP2.159	chr5A	653281499	653281903	-1	M-42-C-10-C-21-CC-11-C-1-C-25-C-9-C-6	30	105	11.09	5.01
TaLTP2.160	chr5A	653284131	653284535	-1	M-42-C-10-C-21-CC-11-C-1-C-25-C-9-C-6	30	105	11.09	5.01
TaLTP2.161	chr5A	663375905	663376207	-1	M-24-C-9-C-17-CC-9-C-1-C-24-C-7-C-0	25	76	8.13	4.34
TaLTP2.162	chr5A	691976837	691977286	1	M-62-C-9-C-21-CC-13-C-1-C-24-C-9-C-1	26	124	12.01	9.19
TaLTP2.163	chr5A	692057989	692058417	-1	M-56-C-9-C-19-CC-13-C-1-C-24-C-9-C-2	23	120	11.44	8
TaLTP2.164	chr5A	692163190	692163696	-1	M-83-C-9-C-18-CC-13-C-1-C-24-C-3-C-8	23	146	14.40	4.86
TaLTP2.165	chr5A	692179351	692179782	-1	M-58-C-9-C-18-CC-13-C-1-C-24-C-3-C-8	23	121	12.08	4.92
TaLTP2.166	chr5A	692236726	692237106	-1	M-40-C-9-C-18-CC-13-C-1-C-25-C-3-C-8	24	103	10.52	7.96
TaLTP2.167	chr5A	692261839	692262243	-1	M-58-C-5-C-18-CC-13-C-1-C-19-C-3-C-8	24	111	11.34	4.63

TaLTP2.168	chr5A	706270655	706270945	1	M-29-C-7-C-13-CC-8-C-1-C-23-C-6-C-0	30	67	7.01	9.4
TaLTP2.169	chr5B	90988334	90988651	1	M-27-C-9-C-16-CC-9-C-1-C-26-C-7-C-1	28	78	8.28	6.77
TaLTP2.170	chr5B	91108881	91109198	1	M-27-C-9-C-16-CC-9-C-1-C-26-C-7-C-1	28	78	8.40	5.66
TaLTP2.171	chr5B	91112112	91112429	1	M-27-C-9-C-16-CC-9-C-1-C-26-C-7-C-1	28	78	8.26	9.87
TaLTP2.172	chr5B	526597643	526598041	1	M-46-C-9-C-18-CC-13-C-1-C-25-C-9-C-2	23	110	11.45	5.55
TaLTP2.173	chr5B	526633884	526634274	1	M-32-C-9-C-18-CC-13-C-1-C-25-C-9-C-2	27	92	9.69	5.55
TaLTP2.174	chr5B	526654817	526655215	1	M-46-C-9-C-18-CC-13-C-1-C-25-C-3-C-8	23	110	11.51	6.92
TaLTP2.175	chr5B	689336940	689337224	-1	M-26-C-7-C-13-CC-8-C-1-C-23-C-6-C-1	27	68	7.06	8.49
TaLTP2.176	chr5B	689340369	689340656	-1	M-28-C-7-C-13-CC-8-C-1-C-23-C-6-C-0	29	67	7.03	9.4
TaLTP2.177	chr5D	84185677	84185994	1	M-27-C-9-C-16-CC-9-C-1-C-26-C-7-C-1	28	78	8.31	9.87
TaLTP2.178	chr5D	433058166	433058564	1	M-46-C-9-C-18-CC-13-C-1-C-25-C-9-C-2	23	110	11.48	5.55
TaLTP2.179	chr5D	433109024	433109422	1	M-46-C-9-C-18-CC-13-C-1-C-25-C-9-C-2	23	110	11.59	6.03
TaLTP2.180	chr6A	65073332	65073841	-1	M-68-C-9-C-15-CC-12-C-1-C-27-C-11-C-17	25	145	14.92	9.74
TaLTP2.181	chr6A	65133550	65134074	-1	M-73-C-9-C-15-CC-12-C-1-C-27-C-11-C-17	25	150	15.45	8.16
TaLTP2.182	chr6A	459677544	459677924	-1	M-42-C-9-C-18-CC-13-C-1-C-24-C-9-C-1	23	104	10.47	5.57
TaLTP2.183	chr6B	121529231	121529737	-1	M-69-C-9-C-15-CC-12-C-1-C-27-C-9-C-17	25	144	14.69	9.36
TaLTP2.184	chr6B	123724068	123724529	1	M-47-C-9-C-15-CC-12-C-1-C-27-C-11-C-22	27	127	13.13	4.49
TaLTP2.185	chr6B	501128173	501128553	1	M-42-C-9-C-18-CC-13-C-1-C-24-C-9-C-1	23	104	10.41	5.61
TaLTP2.186	chr6D	47810554	47811000	-1	M-48-C-9-C-15-CC-12-C-1-C-27-C-11-C-16	22	127	13.09	4.02
TaLTP2.187	chr6D	49848829	49849353	1	M-73-C-9-C-15-CC-12-C-1-C-27-C-11-C-17	25	150	15.35	9.8
TaLTP2.188	chr6D	49855206	49855724	1	M-73-C-9-C-15-CC-12-C-1-C-27-C-9-C-17	25	148	15.14	9.8
TaLTP2.189	chr7A	81607073	81607384	-1	M-26-C-9-C-16-CC-9-C-1-C-26-C-7-C-0	27	77	8.10	10.59
TaLTP2.190	chr7A	498593990	498594400	-1	M-45-C-17-C-15-CC-9-C-1-C-22-C-15-C-3	31	106	10.63	4.27
TaLTP2.191	chr7A	674505756	674506154	-1	M-47-C-9-C-18-CC-13-C-1-C-24-C-3-C-8	23	110	11.33	6.3
TaLTP2.192	chr7A	674520709	674521107	-1	M-47-C-9-C-18-CC-13-C-1-C-24-C-3-C-8	23	110	11.20	7.91
TaLTP2.193	chr7A	674737358	674737756	-1	M-47-C-9-C-18-CC-13-C-1-C-24-C-3-C-8	23	110	11.28	5.05
TaLTP2.194	chr7A	674752092	674752490	-1	M-47-C-9-C-18-CC-13-C-1-C-24-C-3-C-8	23	110	11.28	5.05
TaLTP2.195	chr7A	674796419	674796820	-1	M-48-C-9-C-18-CC-13-C-1-C-24-C-3-C-8	23	111	11.37	6.73
TaLTP2.196	chr7A	674802099	674802500	-1	M-48-C-9-C-18-CC-13-C-1-C-24-C-3-C-8	23	111	11.36	9.03
TaLTP2.197	chr7B	25403834	25404145	-1	M-26-C-9-C-16-CC-9-C-1-C-26-C-7-C-0	27	77	8.10	10.27

TaLTP2.198	chr7B	464548398	464548811	-1	M-44-C-17-C-15-CC-9-C-1-C-22-C-17-C-3	32	106	10.84	4.51
TaLTP2.199	chr7B	558759919	558760203	-1	M-27-C-7-C-13-CC-8-C-1-C-23-C-6-C-0	27	68	6.93	11.31
TaLTP2.200	chr7B	558992275	558992559	-1	M-27-C-7-C-13-CC-8-C-1-C-23-C-6-C-0	27	68	6.99	11.31
TaLTP2.201	chr7B	650294286	650294684	-1	M-47-C-9-C-18-CC-13-C-1-C-24-C-3-C-8	23	110	11.24	7.91
TaLTP2.202	chr7B	650300433	650300831	-1	M-47-C-9-C-18-CC-13-C-1-C-24-C-3-C-8	23	110	11.28	7.91
TaLTP2.203	chr7B	650317214	650317612	-1	M-47-C-9-C-18-CC-13-C-1-C-24-C-3-C-8	23	110	11.38	7.91
TaLTP2.204	chr7B	650361486	650361884	-1	M-47-C-9-C-18-CC-13-C-1-C-24-C-3-C-8	23	110	11.32	8.58
TaLTP2.205	chr7B	650408469	650408867	-1	M-47-C-9-C-18-CC-13-C-1-C-24-C-3-C-8	23	110	11.31	8.58
TaLTP2.206	chr7B	650455225	650455623	-1	M-47-C-9-C-18-CC-13-C-1-C-24-C-3-C-8	21	112	11.45	7.91
TaLTP2.207	chr7B	650539649	650540050	-1	M-48-C-9-C-18-CC-13-C-1-C-24-C-3-C-8	23	111	11.43	8.97
TaLTP2.208	chr7B	650550199	650550600	-1	M-48-C-9-C-18-CC-13-C-1-C-24-C-3-C-8	23	111	11.41	8.97
TaLTP2.209	chr7B	650561737	650562138	-1	M-48-C-9-C-18-CC-13-C-1-C-24-C-3-C-8	23	111	11.47	8.58
TaLTP2.210	chr7B	650573579	650573980	-1	M-48-C-9-C-18-CC-13-C-1-C-24-C-3-C-8	23	111	11.40	8.97
TaLTP2.211	chr7B	650584509	650584910	-1	M-48-C-9-C-18-CC-13-C-1-C-24-C-3-C-8	23	111	11.41	8.97
TaLTP2.212	chr7D	5277144	5277443	1	M-29-C-5-C-16-CC-9-C-1-C-18-C-3-C-9	26	74	7.92	10.57
TaLTP2.213	chr7D	15402480	15402749	-1	M-26-C-8-C-17-CC-9-C-1-C-15-C-4-C-0	26	64	6.92	5.48
TaLTP2.214	chr7D	447061732	447062142	-1	M-44-C-17-C-15-CC-9-C-1-C-22-C-16-C-3	31	106	10.87	4.47
TaLTP2.215	chr7D	523392670	523392951	1	M-26-C-7-C-13-CC-8-C-1-C-23-C-6-C-0	26	68	6.99	11.31
TaLTP2.216	chr7D	582544930	582545331	-1	M-48-C-9-C-18-CC-13-C-1-C-24-C-3-C-8	21	113	11.66	6.27
TaLTP2.217	chr7D	582556602	582557002	-1	M-31-C-9-C-18-CC-13-C-1-C-24-C-3-C-8	23	94	9.82	6.74
TaLTP2.218	chr7D	582561126	582561526	-1	M-25-C-5-C-18-CC-13-C-1-C-24-C-3-C-8	23	84	8.79	9.19
TaLTP2.219	chr7D	582650386	582650787	-1	M-48-C-9-C-18-CC-13-C-1-C-24-C-3-C-8	23	111	11.52	5.04
TaLTP2.220	chr7D	582661777	582662177	-1	M-28-C-9-C-18-CC-13-C-1-C-24-C-3-C-8	23	91	9.54	6.27
TaLTP2.221	chr7D	582747483	582747878	-1	M-47-C-9-C-18-CC-13-C-1-C-23-C-3-C-8	23	109	11.22	5.61
TaLTP2.222	chrUn	29789284	29789571	1	M-28-C-7-C-13-CC-8-C-1-C-23-C-6-C-0	29	67	7.14	7.11
TaLTP2.223	chrUn	29798592	29798882	1	M-29-C-7-C-13-CC-8-C-1-C-23-C-6-C-0	30	67	7.05	9.4
TaLTP2.224	chrUn	29990173	29990454	-1	M-26-C-7-C-13-CC-8-C-1-C-23-C-6-C-0	27	67	6.87	8.49
TaLTP2.225	chrUn	43664829	43665176	1	M-27-C-9-C-14-CC-19-C-1-C-19-C-13-C-4	26	90	8.68	10.75
TaLTP2.226	chrUn	43770507	43770854	1	M-27-C-9-C-14-CC-19-C-1-C-19-C-13-C-4	26	90	8.64	10.75
TaLTP2.227	chrUn	44271349	44271696	1	M-27-C-9-C-14-CC-19-C-1-C-19-C-13-C-4	26	90	8.62	10.75

TaLTP2.228	chrUn	44297183	44297530	1	M-27-C-9-C-14-CC-19-C-1-C-19-C-13-C-4	26	90	8.62	10.75
TaLTP2.229	chrUn	44500426	44500773	-1	M-27-C-9-C-14-CC-19-C-1-C-19-C-13-C-4	26	90	8.62	10.75
TaLTP2.230	chrUn	44529121	44529468	-1	M-27-C-9-C-14-CC-19-C-1-C-19-C-13-C-4	26	90	8.62	10.75
TaLTP2.231	chrUn	60683260	60683673	1	M-62-C-5-C-17-CC-13-C-1-C-19-C-3-C-8	26	112	11.68	4.23
TaLTP2.232	chrUn	60741011	60741424	1	M-62-C-5-C-17-CC-13-C-1-C-19-C-3-C-8	26	112	11.66	4.17
TaLTP2.233	chrUn	154527864	154528298	1	M-69-C-5-C-17-CC-13-C-1-C-19-C-3-C-8	24	121	12.48	4.83
TaLTP2.234	chrUn	154531224	154531760	1	M-69-C-5-C-17-CC-13-C-1-C-36-C-3-C-25	24	155	16.42	9.3
TaLTP2.235	chrUn	154535835	154536257	1	M-65-C-5-C-17-CC-13-C-1-C-19-C-3-C-8	24	117	12.19	5.53
TaLTP2.236	chrUn	154542607	154542996	1	M-43-C-9-C-18-CC-13-C-1-C-25-C-3-C-8	24	106	10.82	7.95
TaLTP2.237	chrUn	154586875	154587306	1	M-58-C-9-C-18-CC-13-C-1-C-24-C-3-C-8	23	121	12.10	6.21
TaLTP2.238	chrUn	154601510	154601974	1	M-58-C-9-C-18-CC-13-C-1-C-24-C-3-C-8	23	121	12.00	6.18
TaLTP2.239	chrUn	154617362	154617793	1	M-58-C-9-C-18-CC-13-C-1-C-24-C-3-C-8	23	121	12.16	4.86
TaLTP2.240	chrUn	154629667	154630185	1	M-87-C-9-C-18-CC-13-C-1-C-24-C-3-C-8	23	150	14.67	6.18
TaLTP2.241	chrUn	168641413	168641760	-1	M-27-C-9-C-14-CC-19-C-1-C-19-C-13-C-4	26	90	8.66	10.75
TaLTP2.242	chrUn	168644994	168645341	-1	M-27-C-9-C-14-CC-19-C-1-C-19-C-13-C-4	26	90	8.70	10.75
TaLTP2.243	chrUn	168709387	168709734	-1	M-27-C-9-C-14-CC-19-C-1-C-19-C-13-C-4	26	90	8.66	10.75
TaLTP2.244	chrUn	189561514	189561903	-1	M-43-C-9-C-18-CC-13-C-1-C-25-C-3-C-8	24	106	10.79	7.95
TaLTP2.245	chrUn	189575201	189575590	-1	M-43-C-9-C-18-CC-13-C-1-C-25-C-3-C-8	24	106	10.80	7.95
TaLTP2.246	chrUn	189578553	189578939	-1	M-53-C-5-C-17-CC-13-C-1-C-19-C-3-C-8	24	105	11.14	4.9
TaLTP2.247	chrUn	201362401	201362823	1	M-65-C-5-C-17-CC-13-C-1-C-19-C-3-C-8	24	117	12.27	4.9
TaLTP2.248	chrUn	201365776	201366210	1	M-69-C-5-C-17-CC-13-C-1-C-19-C-3-C-8	24	121	12.58	4.18
TaLTP2.249	chrUn	201369161	201369550	1	M-43-C-9-C-18-CC-13-C-1-C-25-C-3-C-8	24	106	10.77	8.04
TaLTP2.250	chrUn	201383979	201384413	1	M-69-C-5-C-17-CC-13-C-1-C-19-C-3-C-8	24	121	12.61	4.17
TaLTP2.251	chrUn	201387365	201387784	1	M-64-C-5-C-17-CC-13-C-1-C-19-C-3-C-8	24	116	12.15	4.5
TaLTP2.252	chrUn	201389948	201390382	1	M-69-C-5-C-17-CC-13-C-1-C-19-C-3-C-8	24	121	12.63	4.5
TaLTP2.253	chrUn	202839683	202840030	-1	M-27-C-9-C-14-CC-19-C-1-C-19-C-13-C-4	26	90	8.70	10.75
TaLTP2.254	chrUn	248122351	248122668	-1	M-27-C-9-C-16-CC-9-C-1-C-26-C-7-C-1	28	78	8.24	9.52
TaLTP2.255	chrUn	248126327	248126644	-1	M-27-C-9-C-16-CC-9-C-1-C-26-C-7-C-1	28	78	8.29	9.78
TaLTP2.256	chrUn	272422375	272422794	-1	M-64-C-5-C-17-CC-13-C-1-C-19-C-3-C-8	24	116	12.13	4.5
TaLTP2.257	chrUn	272425745	272426164	1	M-64-C-5-C-17-CC-13-C-1-C-19-C-3-C-8	24	116	12.15	4.5

TaLTP2.258	chrUn	279539920	279540339	1	M-64-C-5-C-17-CC-13-C-1-C-19-C-3-C-8	24	116	12.22	4.5
TaLTP2.259	chrUn	279543298	279543687	1	M-43-C-9-C-18-CC-13-C-1-C-25-C-3-C-8	24	106	10.81	7.96
TaLTP2.260	chrUn	281905703	281906020	1	M-27-C-9-C-16-CC-9-C-1-C-26-C-7-C-1	28	78	8.22	9.52
TaLTP2.261	chrUn	291090280	291090702	1	M-65-C-5-C-17-CC-13-C-1-C-19-C-3-C-8	24	117	12.14	4.83
TaLTP2.262	chrUn	316645401	316645748	1	M-27-C-9-C-14-CC-19-C-1-C-19-C-13-C-4	26	90	8.77	10.52
TaLTP2.263	chrUn	352901801	352902148	-1	M-27-C-9-C-14-CC-19-C-1-C-19-C-13-C-4	26	90	8.70	10.75
TaLTP2.264	chrUn	363336879	363337268	1	M-43-C-9-C-18-CC-13-C-1-C-25-C-3-C-8	24	106	10.82	7.95
TaLTP2.265	chrUn	382657136	382657570	1	M-69-C-5-C-17-CC-13-C-1-C-19-C-3-C-8	24	121	12.63	4.5
TaLTP2.266	chrUn	384745684	384746118	1	M-69-C-5-C-17-CC-13-C-1-C-19-C-3-C-8	24	121	12.65	4.5
TaLTP2.267	chrUn	386578210	386578557	-1	M-27-C-9-C-14-CC-19-C-1-C-19-C-13-C-4	26	90	8.66	10.75
TaLTP2.268	chrUn	390420707	390421054	-1	M-27-C-9-C-14-CC-19-C-1-C-19-C-13-C-4	26	90	8.70	10.75
TaLTP2.269	chrUn	391869713	391870147	1	M-69-C-5-C-17-CC-13-C-1-C-19-C-3-C-8	24	121	12.63	4.43
TaLTP2.270	chrUn	405745791	405746225	1	M-69-C-5-C-17-CC-13-C-1-C-19-C-3-C-8	24	121	12.61	4.17
TaLTP2.271	chrUn	410676270	410676689	1	M-64-C-5-C-17-CC-13-C-1-C-19-C-3-C-8	24	116	12.22	4.5
TaLTP2.272	chrUn	411262600	411263034	1	M-69-C-5-C-17-CC-13-C-1-C-19-C-3-C-8	24	121	12.67	4.5
TaLTP2.273	chrUn	413773129	413773542	1	M-62-C-5-C-17-CC-13-C-1-C-19-C-3-C-8	26	112	11.72	4.52
TaLTP2.274	chrUn	416009590	416010006	1	M-63-C-5-C-17-CC-13-C-1-C-19-C-3-C-8	26	113	11.79	5.47
TaLTPc.1	chr5A	524240635	524241108	1	M-37-C-9-C-19-CC-9-C-1-C-12-C-6-C-31	33	101	10.57	9.82
TaLTPc.2	chr5B	496521936	496522402	1	M-37-C-9-C-19-CC-9-C-1-C-12-C-6-C-30	33	100	10.47	9.62
TaLTPc.3	chr5D	412360925	412361358	1	M-36-C-9-C-19-CC-9-C-1-C-12-C-6-C-1	32	71	7.11	9.97
TaLTPc.4	chr7A	275916291	275916705	1	M-33-C-9-C-14-CC-9-C-1-C-12-C-6-C-2	30	66	6.80	10.61
TaLTPc.5	chr7B	234590456	234590876	1	M-33-C-9-C-14-CC-9-C-1-C-12-C-6-C-2	30	66	6.73	10.61
TaLTPd.1	chr1A	42670763	42671250	1	M-21-C-10-C-17-CC-9-C-1-C-11-C-10-C-19	21	87	9.88	9.38
TaLTPd.2	chr1A	42695478	42695965	1	M-21-C-10-C-17-CC-9-C-1-C-11-C-10-C-19	21	87	9.83	6.78
TaLTPd.3	chr1A	42721347	42721834	1	M-21-C-10-C-17-CC-9-C-1-C-11-C-10-C-19	21	87	9.87	9.38
TaLTPd.4	chr1A	43270923	43271411	-1	M-21-C-10-C-17-CC-9-C-1-C-11-C-10-C-19	20	88	9.86	8.69
TaLTPd.5	chr1A	292077106	292077622	1	M-27-C-10-C-16-CC-9-C-1-C-23-C-7-C-5	25	83	8.61	4.24
TaLTPd.6	chr1B	63202243	63202730	1	M-21-C-10-C-17-CC-9-C-1-C-11-C-10-C-19	21	87	9.78	8.71
TaLTPd.7	chr1B	63468788	63469269	1	M-21-C-10-C-17-CC-9-C-1-C-11-C-10-C-17	21	85	9.59	9
TaLTPd.8	chr1B	63480020	63480507	1	M-21-C-10-C-17-CC-9-C-1-C-11-C-10-C-19	21	87	9.79	9

TaLTPd.9	chr1B	63614431	63614918	1	M-21-C-10-C-17-CC-9-C-1-C-11-C-10-C-19	21	87	9.80	9.02
TaLTPd.10	chr1B	63809033	63809520	1	M-21-C-10-C-17-CC-9-C-1-C-11-C-10-C-19	21	87	9.79	8.68
TaLTPd.11	chr1B	63889321	63889808	1	M-21-C-10-C-17-CC-9-C-1-C-11-C-10-C-19	21	87	9.79	9
TaLTPd.12	chr1B	325818004	325818534	-1	M-28-C-10-C-16-CC-9-C-1-C-23-C-7-C-5	25	84	8.83	4.56
TaLTPd.13	chr1B	645244411	645244868	1	M-21-C-10-C-17-CC-9-C-1-C-11-C-10-C-19	21	87	10.04	9.48
TaLTPd.14	chr1D	43306213	43306700	1	M-21-C-10-C-17-CC-9-C-1-C-11-C-10-C-19	21	87	9.81	8.71
TaLTPd.15	chr1D	43823484	43823971	1	M-21-C-10-C-17-CC-9-C-1-C-11-C-10-C-19	21	87	9.79	8.68
TaLTPd.16	chr1D	44275397	44275864	1	M-21-C-10-C-17-CC-9-C-1-C-11-C-10-C-20	21	88	9.99	9
TaLTPd.17	chr1D	44310595	44311158	1	M-21-C-10-C-17-CC-9-C-1-C-11-C-10-C-30	21	98	10.75	7.93
TaLTPd.18	chr1D	44629280	44629767	1	M-21-C-10-C-17-CC-9-C-1-C-11-C-10-C-19	21	87	9.80	8.68
TaLTPd.19	chr1D	225710372	225710900	-1	M-27-C-10-C-16-CC-9-C-1-C-23-C-7-C-5	20	88	9.03	4.52
TaLTPd.20	chr2A	25449735	25450711	-1	M-96-C-9-C-17-CC-12-C-1-C-27-C-9-C-40	23	198	20.97	4.55
TaLTPd.21	chr2A	158073110	158073760	-1	M-84-C-9-C-17-CC-12-C-1-C-27-C-9-C-17	27	159	16.79	5.29
TaLTPd.22	chr2A	511095229	511095672	1	M-29-C-14-C-14-CC-12-C-1-C-24-C-10-C-6	28	92	9.30	9.85
TaLTPd.23	chr2A	511114764	511115263	1	M-29-C-14-C-14-CC-12-C-1-C-24-C-10-C-12	28	98	10.45	5.04
TaLTPd.24	chr2A	684199222	684199709	1	M-26-C-13-C-14-CC-12-C-1-C-23-C-4-C-6	25	84	8.58	11.45
TaLTPd.25	chr2B	36915011	36916039	-1	M-99-C-9-C-17-CC-12-C-1-C-27-C-9-C-43	26	201	21.72	4.59
TaLTPd.26	chr2B	36920840	36921868	-1	M-99-C-9-C-17-CC-12-C-1-C-27-C-9-C-43	26	201	21.73	4.59
TaLTPd.27	chr2B	36926736	36927764	-1	M-99-C-9-C-17-CC-12-C-1-C-27-C-9-C-43	26	201	21.73	4.59
TaLTPd.28	chr2B	36938142	36939170	-1	M-99-C-9-C-17-CC-12-C-1-C-27-C-9-C-43	26	201	21.73	4.59
TaLTPd.29	chr2B	36944050	36945078	-1	M-99-C-9-C-17-CC-12-C-1-C-27-C-9-C-43	26	201	21.72	4.59
TaLTPd.30	chr2B	200896772	200897422	1	M-82-C-9-C-17-CC-12-C-1-C-27-C-11-C-18	27	160	17.05	8.41
TaLTPd.31	chr2B	448778246	448778688	1	M-29-C-14-C-14-CC-12-C-1-C-24-C-10-C-6	28	92	9.38	9.85
TaLTPd.32	chr2B	448870450	448870945	1	M-29-C-14-C-14-CC-12-C-1-C-24-C-10-C-12	28	98	10.51	8.97
TaLTPd.33	chr2B	646066328	646066819	1	M-26-C-12-C-14-CC-12-C-1-C-24-C-4-C-6	25	84	8.69	10.4
TaLTPd.34	chr2D	143221549	143222200	1	M-84-C-9-C-17-CC-12-C-1-C-27-C-9-C-17	27	159	16.79	5.29
TaLTPd.35	chr2D	143236318	143236976	1	M-85-C-9-C-17-CC-12-C-1-C-27-C-9-C-17	27	160	17.09	8.76
TaLTPd.36	chr2D	377150417	377150853	1	M-27-C-14-C-14-CC-12-C-1-C-24-C-10-C-6	26	92	9.35	9.85
TaLTPd.37	chr2D	377156149	377156644	1	M-29-C-14-C-14-CC-12-C-1-C-24-C-10-C-12	28	98	10.48	8.19
TaLTPd.38	chr3A	574864267	574864817	1	M-48-C-10-C-17-CC-9-C-1-C-11-C-10-C-20	21	115	12.10	4.77

TaLTPd.39	chr3A	574866319	574866734	1	M-20-C-10-C-17-CC-9-C-1-C-11-C-10-C-20	20	88	9.55	9.08
TaLTPd.40	chr3A	613171334	613171851	-1	M-27-C-14-C-14-CC-11-C-1-C-24-C-10-C-14	26	99	10.28	11.84
TaLTPd.41	chr3B	571809668	571810254	1	M-60-C-10-C-17-CC-9-C-1-C-11-C-10-C-20	21	127	13.39	4.58
TaLTPd.42	chr3B	571811742	571812157	1	M-20-C-10-C-17-CC-9-C-1-C-11-C-20-C-10	20	88	9.79	9.4
TaLTPd.43	chr3B	625332980	625333477	-1	M-27-C-14-C-14-CC-11-C-1-C-24-C-10-C-30	26	115	12.38	10.27
TaLTPd.44	chr3D	436042498	436043075	1	M-57-C-10-C-17-CC-9-C-1-C-11-C-10-C-20	21	124	13.00	5.07
TaLTPd.45	chr3D	436044575	436044990	1	M-20-C-10-C-17-CC-9-C-1-C-11-C-10-C-20	20	88	9.61	9.08
TaLTPd.46	chr3D	436163790	436164205	1	M-20-C-10-C-17-CC-9-C-1-C-11-C-10-C-20	20	88	9.56	8.74
TaLTPd.47	chr3D	470888758	470889215	-1	M-27-C-14-C-14-CC-11-C-1-C-24-C-10-C-6	26	91	9.28	11.65
TaLTPd.48	chr5B	600728083	600728567	1	M-21-C-10-C-17-CC-9-C-1-C-11-C-10-C-19	21	87	9.84	8.71
TaLTPd.49	chr5D	488203890	488204374	1	M-21-C-10-C-17-CC-9-C-1-C-11-C-10-C-19	21	87	9.85	9.22
TaLTPd.50	chr6B	17036701	17037146	-1	M-27-C-14-C-14-CC-12-C-1-C-24-C-10-C-6	26	92	9.34	9.7
TaLTPd.51	chr7A	644845693	644846250	1	M-29-C-14-C-14-CC-11-C-1-C-24-C-10-C-6	26	93	9.55	8.4
TaLTPd.52	chr7A	656982419	656982982	1	M-32-C-10-C-18-CC-10-C-1-C-17-C-4-C-41	26	117	12.91	9.47
TaLTPd.53	chr7A	676065583	676066434	-1	M-87-C-9-C-17-CC-12-C-1-C-27-C-9-C-5	28	149	15.68	7.89
TaLTPd.54	chr7A	676277892	676278720	1	M-87-C-9-C-17-CC-12-C-1-C-27-C-9-C-5	29	148	15.57	7.83
TaLTPd.55	chr7B	608470317	608470782	1	M-29-C-14-C-14-CC-11-C-1-C-24-C-10-C-6	22	97	9.80	8.34
TaLTPd.56	chr7D	560706749	560708577	-1	M-29-C-14-C-14-CC-11-C-1-C-24-C-10-C-32	22	123	12.54	8.87
TaLTPd.57	chr7D	585603227	585604080	-1	M-87-C-9-C-17-CC-12-C-1-C-27-C-9-C-4	28	148	15.67	7.89
TaLTPg.1	chr1A	30196854	30197476	-1	M-29-C-9-C-14-CC-12-C-1-C-26-C-9-C-61	25	120	11.70	8.34
TaLTPg.2	chr1A	498174489	498175684	-1	M-59-C-9-C-16-CC-12-C-1-C-25-C-9-C-74	34	156	15.27	8.28
TaLTPg.3	chr1B	48346465	48347106	1	M-25-C-9-C-14-CC-12-C-1-C-26-C-9-C-79	22	143	13.77	9.23
TaLTPg.4	chr1B	541011245	541012356	1	M-59-C-9-C-16-CC-12-C-1-C-25-C-9-C-74	34	156	15.26	8.28
TaLTPg.5	chr1D	30107700	30108330	-1	M-31-C-9-C-14-CC-12-C-1-C-26-C-9-C-61	28	119	11.56	8.34
TaLTPg.6	chr1D	403059303	403060402	-1	M-60-C-9-C-16-CC-12-C-1-C-25-C-9-C-74	34	157	15.34	8.28
TaLTPg.7	chr2A	113928376	113929907	-1	M-37-C-9-C-16-CC-14-C-1-C-22-C-9-C-62	26	127	11.87	9.46
TaLTPg.8	chr2A	349721024	349721944	1	M-23-C-9-C-14-CC-12-C-1-C-25-C-9-C-62	21	115	11.84	4.45
TaLTPg.9	chr2A	571149720	571150423	-1	M-37-C-9-C-14-CC-12-C-1-C-26-C-10-C-78	26	146	14.66	4.75
TaLTPg.10	chr2A	674493788	674494564	-1	M-29-C-10-C-18-CC-12-C-1-C-24-C-8-C-57	25	123	12.47	8.59
TaLTPg.11	chr2B	162928065	162929589	-1	M-36-C-9-C-16-CC-14-C-1-C-22-C-9-C-62	25	127	11.98	9.46

TaLTPg.12	chr2B	318204810	318206745	-1	M-23-C-9-C-14-CC-12-C-1-C-25-C-9-C-62	21	115	11.89	4.45
TaLTPg.13	chr2B	491022663	491023363	1	M-34-C-9-C-14-CC-12-C-1-C-26-C-10-C-78	23	146	14.66	4.45
TaLTPg.14	chr2B	629693498	629694274	-1	M-29-C-10-C-18-CC-12-C-1-C-24-C-8-C-78	25	144	14.51	6.28
TaLTPg.15	chr2D	112719725	112721413	-1	M-36-C-9-C-16-CC-14-C-1-C-22-C-9-C-62	25	127	11.90	9.46
TaLTPg.16	chr2D	279055262	279056172	-1	M-23-C-9-C-14-CC-12-C-1-C-25-C-9-C-62	19	117	11.95	4.45
TaLTPg.17	chr2D	419024868	419025582	1	M-37-C-9-C-14-CC-12-C-1-C-26-C-10-C-78	26	146	14.54	4.65
TaLTPg.18	chr2D	529657261	529658067	-1	M-29-C-10-C-18-CC-12-C-1-C-24-C-8-C-80	25	144	14.52	6.28
TaLTPg.19	chr3A	395763172	395764865	-1	M-32-C-9-C-20-CC-19-C-1-C-24-C-20-C-79	24	165	16.53	5.26
TaLTPg.20	chr3A	584766039	584767648	1	M-41-C-9-C-16-CC-12-C-1-C-25-C-9-C-67	27	136	13.54	5.12
TaLTPg.21	chr3B	581663498	581664752	1	M-41-C-9-C-16-CC-12-C-1-C-25-C-9-C-67	27	136	13.53	5.68
TaLTPg.22	chr3D	298061090	298062754	-1	M-32-C-9-C-20-CC-19-C-1-C-24-C-20-C-79	24	159	15.88	5.25
TaLTPg.23	chr3D	443628687	443630068	1	M-41-C-9-C-16-CC-12-C-1-C-25-C-9-C-68	27	137	13.62	5.68
TaLTPg.24	chr4A	14835749	14837105	-1	M-33-C-9-C-14-CC-12-C-1-C-26-C-9-C-83	22	144	14.60	6.98
TaLTPg.25	chr4A	30399234	30400445	-1	M-32-C-9-C-16-CC-12-C-1-C-24-C-9-C-77	23	142	13.87	5.68
TaLTPg.26	chr4A	168545335	168546427	1	M-42-C-9-C-16-CC-12-C-1-C-24-C-9-C-63	26	131	12.68	4.65
TaLTPg.27	chr4A	596215050	596215755	1	M-47-C-9-C-14-CC-12-C-1-C-21-C-6-C-87	31	148	14.01	4.14
TaLTPg.28	chr4A	596238542	596239241	-1	M-46-C-9-C-14-CC-12-C-1-C-21-C-6-C-86	31	147	13.91	4.14
TaLTPg.29	chr4A	596298088	596299944	1	M-31-C-12-C-8-CC-14-C-1-C-24-C-13-C-110	24	174	17.05	6.03
TaLTPg.30	chr4B	13125606	13127461	-1	M-27-C-12-C-8-CC-14-C-1-C-24-C-13-C-112	24	172	16.76	6.19
TaLTPg.31	chr4B	13277411	13278102	-1	M-46-C-9-C-14-CC-12-C-1-C-21-C-6-C-83	28	146	13.77	3.85
TaLTPg.32	chr4B	565313012	565314547	1	M-33-C-9-C-14-CC-12-C-1-C-26-C-9-C-83	27	139	14.08	6.08
TaLTPg.33	chr4D	7215791	7217463	1	M-31-C-12-C-8-CC-14-C-1-C-24-C-13-C-111	24	175	17.16	6.19
TaLTPg.34	chr4D	7409439	7410145	1	M-47-C-9-C-14-CC-12-C-1-C-21-C-6-C-86	32	147	13.82	4.14
TaLTPg.35	chr4D	7411916	7412692	1	M-30-C-9-C-14-CC-12-C-1-C-24-C-6-C-122	24	179	17.75	4.08
TaLTPg.36	chr4D	308544569	308545738	-1	M-42-C-9-C-16-CC-12-C-1-C-24-C-9-C-63	26	133	12.82	4.61
TaLTPg.37	chr4D	437683745	437685153	1	M-40-C-9-C-16-CC-12-C-1-C-24-C-9-C-73	31	139	13.61	5.18
TaLTPg.38	chr4D	451801760	451803035	-1	M-23-C-9-C-14-CC-12-C-1-C-26-C-9-C-83	17	139	14.05	6.08
TaLTPg.39	chr5A	491948772	491949564	-1	M-35-C-9-C-14-CC-12-C-1-C-26-C-9-C-82	22	151	15.25	4.17
TaLTPg.40	chr5A	616909276	616909794	-1	M-30-C-9-C-14-CC-12-C-1-C-26-C-9-C-62	29	118	11.75	8.3
TaLTPg.41	chr5A	616914032	616914550	-1	M-30-C-9-C-14-CC-12-C-1-C-26-C-9-C-62	29	118	11.68	8.27

TaLTPg.42	chr5A	616919394	616919912	-1	M-30-C-9-C-14-CC-12-C-1-C-26-C-9-C-62	29	118	11.58	9.16
TaLTPg.43	chr5A	616981829	616982464	-1	M-26-C-9-C-14-CC-12-C-1-C-26-C-9-C-66	25	122	11.84	9.6
TaLTPg.44	chr5A	617173798	617174627	-1	M-26-C-10-C-17-CC-12-C-1-C-24-C-8-C-80	21	143	14.35	8.74
TaLTPg.45	chr5A	617175190	617175916	1	M-38-C-9-C-14-CC-12-C-1-C-25-C-9-C-64	20	132	12.59	4.93
TaLTPg.46	chr5A	617188181	617188922	1	M-44-C-9-C-14-CC-12-C-1-C-25-C-9-C-61	20	138	13.27	4.46
TaLTPg.47	chr5A	634077376	634079042	1	M-33-C-9-C-16-CC-12-C-1-C-26-C-8-C-77	27	137	14.50	8.97
TaLTPg.48	chr5A	634086223	634087886	1	M-30-C-9-C-16-CC-12-C-1-C-26-C-8-C-74	24	132	13.87	8.18
TaLTPg.49	chr5B	467227042	467227827	-1	M-33-C-9-C-14-CC-12-C-1-C-26-C-9-C-56	29	116	11.84	4.16
TaLTPg.50	chr5B	609927335	609927847	-1	M-30-C-9-C-14-CC-12-C-1-C-26-C-9-C-60	29	116	11.47	8.24
TaLTPg.51	chr5B	609935235	609935753	-1	M-30-C-9-C-14-CC-12-C-1-C-26-C-9-C-62	29	118	11.74	8.27
TaLTPg.52	chr5B	610007128	610007768	-1	M-36-C-9-C-14-CC-12-C-1-C-26-C-9-C-53	35	108	10.52	9.31
TaLTPg.53	chr5B	610022234	610022905	-1	M-36-C-9-C-14-CC-12-C-1-C-26-C-9-C-66	35	122	11.88	9.78
TaLTPg.54	chr5B	610081075	610081832	-1	M-36-C-9-C-14-CC-12-C-1-C-26-C-9-C-96	35	178	17.65	9.01
TaLTPg.55	chr5B	610085156	610085805	-1	M-36-C-9-C-14-CC-12-C-1-C-26-C-9-C-61	35	117	11.34	9.23
TaLTPg.56	chr5B	610293664	610294516	-1	M-30-C-10-C-17-CC-12-C-1-C-24-C-8-C-80	25	143	14.33	8.74
TaLTPg.57	chr5B	610295057	610295797	1	M-44-C-9-C-14-CC-12-C-1-C-25-C-9-C-63	31	132	12.78	5.55
TaLTPg.58	chr5B	637143796	637145288	1	M-35-C-9-C-16-CC-12-C-1-C-26-C-8-C-76	29	135	14.35	9.3
TaLTPg.59	chr5B	637158254	637159814	1	M-30-C-9-C-16-CC-12-C-1-C-26-C-8-C-72	24	131	13.78	8.18
TaLTPg.60	chr5D	388830376	388831166	-1	M-33-C-9-C-14-CC-12-C-1-C-26-C-9-C-81	29	141	14.35	4.37
TaLTPg.61	chr5D	493997499	493998137	1	M-26-C-9-C-14-CC-12-C-1-C-26-C-9-C-66	25	122	11.88	9.6
TaLTPg.62	chr5D	494033291	494033910	1	M-26-C-9-C-14-CC-12-C-1-C-26-C-9-C-66	25	122	11.80	9.6
TaLTPg.63	chr5D	494042019	494042570	1	M-26-C-9-C-14-CC-12-C-1-C-26-C-9-C-77	25	133	13.21	9.63
TaLTPg.64	chr5D	494052848	494053351	1	M-25-C-9-C-14-CC-12-C-1-C-26-C-9-C-62	24	118	11.68	8.27
TaLTPg.65	chr5D	494181604	494182457	-1	M-26-C-10-C-17-CC-12-C-1-C-24-C-8-C-80	21	143	14.34	8.74
TaLTPg.66	chr5D	494183026	494183762	1	M-44-C-9-C-14-CC-12-C-1-C-25-C-9-C-61	29	129	12.38	4.93
TaLTPg.67	chr5D	506269882	506271676	1	M-36-C-9-C-16-CC-12-C-1-C-26-C-8-C-77	30	137	14.44	8.97
TaLTPg.68	chr5D	506290475	506292167	1	M-33-C-9-C-16-CC-12-C-1-C-26-C-8-C-71	27	130	13.68	8.18
TaLTPg.69	chr5D	506946614	506947460	1	M-34-C-9-C-16-CC-12-C-1-C-26-C-8-C-66	28	125	13.05	6.06
TaLTPg.70	chr6A	484065309	484067524	-1	M-32-C-9-C-14-CC-12-C-1-C-26-C-8-C-77	22	138	14.10	5.28
TaLTPg.71	chr6B	518601609	518604075	-1	M-28-C-9-C-14-CC-12-C-1-C-26-C-8-C-77	22	134	13.77	6.41

TaLTPg.72	chr6B	612197715	612199721	1	M-37-C-9-C-16-CC-14-C-1-C-23-C-9-C-53	24	122	11.91	9.17
TaLTPg.73	chr6B	645490602	645491359	-1	M-33-C-10-C-15-CC-12-C-1-C-25-C-8-C-83	27	147	14.29	10.83
TaLTPg.74	chr6D	343636222	343638515	-1	M-28-C-9-C-14-CC-12-C-1-C-26-C-8-C-77	22	134	13.77	6.41
TaLTPg.75	chr6D	428902991	428903766	-1	M-33-C-10-C-15-CC-12-C-1-C-25-C-8-C-85	27	149	14.34	11.55
TaLTPg.76	chr7A	76182477	76183163	1	M-45-C-6-C-13-CC-12-C-1-C-25-C-5-C-32	22	127	13.02	8.46
TaLTPg.77	chr7A	257147699	257152247	1	M-29-C-9-C-14-CC-12-C-1-C-29-C-9-C-61	24	125	13.35	5.38
TaLTPg.78	chr7A	589453828	589455189	1	M-47-C-6-C-13-CC-12-C-1-C-25-C-8-C-41	34	99	9.91	8.3
TaLTPg.79	chr7A	641425898	641428573	-1	M-30-C-6-C-15-CC-12-C-1-C-25-C-8-C-44	25	101	10.27	9.4
TaLTPg.80	chr7B	12149409	12150113	1	M-41-C-6-C-13-CC-12-C-1-C-25-C-5-C-32	22	123	12.73	6.76
TaLTPg.81	chr7B	216815633	216818509	-1	M-29-C-9-C-14-CC-12-C-1-C-29-C-9-C-61	24	125	13.39	5.14
TaLTPg.82	chr7B	548162279	548163672	1	M-47-C-6-C-13-CC-12-C-1-C-25-C-8-C-64	34	122	11.73	5.09
TaLTPg.83	chr7D	69631912	69632637	1	M-43-C-6-C-13-CC-12-C-1-C-25-C-5-C-64	22	133	13.55	6.75
TaLTPg.84	chr7D	241176510	241179983	-1	M-29-C-9-C-14-CC-12-C-1-C-29-C-9-C-137	24	201	21.55	6.31
TaLTPg.85	chr7D	515821376	515822755	1	M-47-C-6-C-13-CC-12-C-1-C-25-C-8-C-64	34	123	11.80	5.09
TaLTPg.86	chr7D	554563423	554565727	1	M-30-C-6-C-15-CC-12-C-1-C-25-C-8-C-51	25	108	10.89	9.4

[1]8CM, eight cysteine motif

[2]AA, number of amino acid

[3]Mw, molecular weight in kilodalton

[4]pI, isoelectric point (cysteine residues engaged in disulphide bridges were removed prior pI calculation)

Supplementary Table S2. Ka/Ks analysis and estimated divergence time for the some *TaLTP* duplicated pairs.

Chr	Gene 1	Position gene 1	Gene 2	Position gene 2	Ka	Ks	Ka/Ks	Identity (%)	Age (MYA)
Chr2A	TaLTP2.48	747727094	TaLTP2.55	747780203	0.0000	0.0000	-	100	0
Chr2A	TaLTP2.55	747780203	TaLTP2.50	747733420	0.0000	0.0000	-	100	0
Chr2A	TaLTP2.50	747733420	TaLTP2.47	747723957	0.0069	0.0000	-	99.5	0
Chr2A	TaLTP2.47	747723957	TaLTP2.54	747777016	0.0103	0.0000	-	99.3	0
Chr2A	TaLTP2.54	747777016	TaLTP2.49	747730232	0.0069	0.0095	0.7267	99.3	0.72978
Chr2A	TaLTP2.49	747730232	TaLTP2.51	747739744	0.0000	0.0000	-	100	0
Chr2A	TaLTP2.51	747739744	TaLTP2.52	747742933	0.0103	0.0096	1.0805	99	0.7354
Chr2A	TaLTP2.52	747742933	TaLTP2.57	747819511	0.0205	0.0536	0.3822	97.3	4.12243
Chr2A	TaLTP2.57	747819511	TaLTP2.58	747874783	0.0446	0.0794	0.5614	94.8	6.10727
Chr2A	TaLTP2.58	747874783	TaLTP2.46	747697212	0.0780	0.1349	0.5785	91.3	10.3785
Chr2A	TaLTP2.46	747697212	TaLTP2.53	747773829	0.0935	0.1160	0.8059	90.8	8.92169
Chr2A	TaLTP2.53	747773829	TaLTP2.56	747809997	0.0034	0.0199	0.1696	99.3	1.5343
Chr3B	TaLTP2.91	35037705	TaLTP2.92	35119171	0.0000	0.0000	-	100	0
Chr3B	TaLTP2.95	35697822	TaLTP2.98	35769759	0.0000	0.0000	-	100	0
Chr3B	TaLTP2.93	35212403	TaLTP2.94	35288587	0.0000	0.0000	-	100	0
Chr3B	TaLTP2.94	35288587	TaLTP2.96	35701446	0.0079	0.0232	0.3394	98.9	1.78377
Chr3B	TaLTP2.96	35701446	TaLTP2.99	35773382	0.0039	0.0000	-	99.7	0
Chr3B	TaLTP2.99	35773382	TaLTP2.97	35754310	0.0380	0.4167	0.0912	90.5	32.0504
Chr3B	TaLTP2.97	35754310	TaLTP2.101	35823275	0.0075	0.0135	0.5555	99.1	1.03502
Chr3B	TaLTP2.101	35823275	TaLTP2.100	35813194	0.0150	0.0278	0.5399	98.3	2.13643
Chr3B	TaLTP2.100	35813194	TaLTP2.102	35837851	0.0150	0.0000	-	98.9	0
Chr3B	TaLTP2.102	35837851	TaLTP2.103	35839140	0.0075	0.0000	-	99.4	0
Chr7B	TaLTP2.204	650361486	TaLTP2.205	650408469	0.0059	0.0191	0.3086	99.2	1.4689
Chr7B	TaLTP2.205	650408469	TaLTP2.203	650317214	0.0179	0.1037	0.1723	97.2	7.974
Chr7B	TaLTP2.203	650317214	TaLTP2.202	650300433	0.0089	0.0816	0.1090	98.2	6.27413
Chr7B	TaLTP2.202	650300433	TaLTP2.206	650455225	0.0090	0.0188	0.4752	99	1.44915
Chr7B	TaLTP2.206	650455225	TaLTP2.201	650294286	0.0089	0.0000	-	99.2	0
Chr7B	TaLTP2.201	650294286	TaLTP2.207	650539649	0.0338	0.7009	0.0482	90.5	53.9148
Chr7B	TaLTP2.207	650539649	TaLTP2.209	650561737	0.0151	0.2535	0.0595	95.5	19.5028
Chr7B	TaLTP2.209	650561737	TaLTP2.208	650550199	0.0030	0.0508	0.0594	99	3.90868
Chr7B	TaLTP2.208	650550199	TaLTP2.210	650573579	0.0030	0.0509	0.0592	99	3.91853
Chr7B	TaLTP2.210	650573579	TaLTP2.211	650584509	0.0060	0.0165	0.3631	99.3	1.26751

Supplementary Table S3. Gene expression data from RNA-seq in log2 FPKM.

	Choulet et al. (2014)										Yang et al. (2015)														
	CSTP_stamen	CSTP_pistil	diplo_dia_rep1	diplo_dia_rep2	latent_lepto_rep1	latent_lepto_rep2	metaphaseI_rep1	metaphaseI_rep2	zygo_pachy_rep1	zygo_pachy_rep2	spike_Z32	spike_Z39	spike_Z65	leaf_Z10	leaf_Z23	leaf_Z71	root_Z10	root_Z13	root_Z39	stem_Z30	stem_Z32	stem_Z65	grain_Z71	grain_Z75	grain_Z85
TaLTP1.1	0	6.891	-2.98	0	0	0	0	-0.91	0	0	-4.68	0	0.478	-1.66	0	-1.39	-0.97	0	0	-2.16	-2.55	0	-1	-2.65	0.169
TaLTP1.10	11.35	0	11.11	11.31	11.51	10.67	10.33	0	11.17	10.83	2.764	0	0	0	0	0	0	0	0	0	0	0	2.554	0	0
TaLTP1.11	7.786	2.921	8.185	7.951	8.192	8.426	8.596	10.08	7.996	7.114	7.506	0	0	-0.48	8.727	8.116	4.35	-2.42	7.584	7.134	6.791	0	7.385	6.079	5.219
TaLTP1.12	11.31	7.651	11.1	11.28	11.51	10.76	10.1	-0.53	10.86	10.82	2.801	0	0	0	0	0	0	0	-3.31	0	0	0	3.199	-3.27	0
TaLTP1.13	6.485	2.542	6.728	6.749	6.872	7.216	7.078	8.599	6.676	5.928	6.114	-3.83	-3.4	-1.84	7.652	6.6	4.268	0	5.624	6.171	5.57	0	5.768	4.351	4.215
TaLTP1.14	11.08	2.689	10.83	11	11.28	10.63	9.796	0	10.64	10.62	2.922	0	0	0	0	0	0	0	0	0	0	0	2.918	0	0
TaLTP1.15	7.635	7.617	7.99	8.015	8.017	8.279	8.286	9.43	8.034	7.094	7.511	-0.62	0	-1.6	8.518	7.923	3.978	-1.13	6.931	7.14	6.67	-1.14	7.228	5.681	5.292
TaLTP1.16	6.701	8.265	6.655	7.219	7.032	7.01	7.262	6.733	7.052	6.289	5.062	-1.94	-1.51	0.003	6.122	4.909	5.606	-1.98	4.082	4.684	4.024	-3.25	5.773	2.971	3.696
TaLTP1.17	5	7.14	4.872	5.473	4.964	4.699	4.667	4.708	4.654	4.856	2.217	-3.52	0	-3.7	5.432	3.72	5.361	-1.98	2.19	4.068	2.622	0	3.222	1.155	3.389
TaLTP1.18	2.146	0	2.127	1.875	2.717	1.358	2.143	0	3.116	2.223	-3.72	0	0	0	0	0	0	0	0	0	0	0	0	0	0
TaLTP1.19	0	0	0	0	0	0	0	0	0	0	0	0.689	0	1.417	0	0	0	1.074	0	0	0	0	0	0	0
TaLTP1.2	0	5.832	0	0	0	-2.67	-3.01	-1.11	0	0	-2.04	-3.75	1.14	-2.92	-0.93	-2.65	0	0	0	-3.69	0	0	-0.53	-1.27	-1.58
TaLTP1.20	7.052	9.391	6.816	7.711	7.275	7.075	7.008	6.995	7.209	6.695	4.906	-3.52	-3.1	-1.11	6.85	5.148	6.124	0	4.626	5.457	4.361	-2.25	5.541	3.399	4.094
TaLTP1.21	2.099	-1.08	1.01	1.529	2.206	1.323	1.339	0	2.679	1.792	-3.1	0	0	0	0	0	0	0	0	0	0	0	0	0	0
TaLTP1.22	-5.56	0.778	-5.3	-5.5	-4.86	-5.38	-4.74	-1.82	-4.85	-5.36	-5.08	-10.4	0.411	-9.14	0.005	-3.02	-3.41	-9.25	0.33	-1.25	-0.85	-0.38	-1.49	-0.94	-2.07
TaLTP1.23	1.636	8.563	1.241	1.191	1.794	2.119	2.417	5.855	1.985	1.558	1.966	-4.52	7.78	-2.7	7.006	4.293	3.83	-4.57	7.215	5.851	6.525	6.907	5.613	6.229	5.235
TaLTP1.24	0	0	0	0	0	-1.28	0	-0.45	0	0	0	0	0	-1.19	0	-1.26	4.07	-1.78	-3.23	-1.71	0	-2.06	-0.12	-2.19	0.127
TaLTP1.25	4.226	0	4.277	3.396	4.649	3.325	3.695	0	4.286	3.815	-2.7	0	0	0	0	0	0	0	0	0	0	0	0	0	0
TaLTP1.26	-2.66	7.945	-1.13	-1.9	0.078	1.047	-0.79	4.074	1.015	-2.57	-0.28	0	7.744	0	7.833	4.882	1.432	-3.57	8.099	5.715	7.693	6.047	6.225	7.289	6.174
TaLTP1.27	0	0	0	0	0	0	0	0	0	0	0	0	-2.92	0.392	0	-0.94	13.84	-0.78	0	0	0	0	4.321	0	10.17
TaLTP1.28	0	0	0	0	0	0	0	0	0	0	0	0	0	-1.52	0	-2.26	13.29	-2.36	-3.23	0	0	0	3.565	0	8.47
TaLTP1.29	1.087	9.425	0.769	0.71	0.878	2.092	2.038	5.552	1.7	1.124	2.485	-0.92	6.856	-2.09	8.554	5.268	4.692	-3.54	8.026	6.243	7.601	5.673	6.3	7.026	5.841
TaLTP1.3	0	6.531	0	0	0	0	0	-1.05	0	0	-4.56	0	-0.35	-3.86	0	0	0	0	0	-3.63	0	0	-1.14	0	-1.14
TaLTP1.30	4.653	9.25	3.983	5.138	4.703	3.205	3.344	3.379	4.848	4.28	-0.93	0	0	0	4.468	0	0	0	1.04	0	0	0	2.651	0	-3.07
TaLTP1.31	0	0	0	0	0	0	0	-1.61	0	0	0	0	0	0	0	0	0	0	0	0	0	0	0	0	0
TaLTP1.32	0	0	0	0	0	0	0	0	0	0	0	0	0	0	0	-3.38	0	0	-3.35	0	0	0	0	0	0
TaLTP1.33	0	0	0	0	0	0	0	0	0	0	0	1.882	0	1.778	0	-2.39	0	2.073	0	-1.42	-2.21	0	0	0	0
TaLTP1.34	0	0	0	0	0	0	0	0	0	0	0	0.126	-3.53	1.5	0	-3.87	0.876	1.186	-3.86	-2.9	-1.38	-3.69	0	-2.22	-4.11
TaLTP1.35	0	0	0	0	0	0	0	-2.03	0	0	0	0	0	-4.52	0	0	0	-3.47	0	0	0	0	0	0	0
TaLTP1.36	0	0	-3.32	0	0	0	0	-3.85	0	0	0	-4.15	0	0	-2.33	0	0	-2.69	0	0	0	0.279	0	-4	0
TaLTP1.37	-3.93	0	0	0	0	0	0	-3.8	0	0	0	0.981	-1.68	2.231	0	-2.43	0	1.025	-1.2	0.412	-1.26	0.742	-2.31	0.135	0
TaLTP1.38	0	0	0	0	0	0	0	-0.5	0	0	0	0	0	0	0	0	-1.29	0	-3.7	0	0	0	0	0	-3.96
TaLTP1.39	0	0	0	-2.27	0	0	0	0	0	0	0	1.694	0	1.907	0	0	0	1.132	0	0	0	0	0	-3.32	0
TaLTP1.4	8.158	10.96	8.06	8.377	8.525	8.715	8.264	10.03	8.483	7.799	6.235	-1.43	-3	0	8.816	7.035	0.903	-1.28	7.599	7.248	9.09	0.315	9.43	7.647	4.976
TaLTP1.5	2.669	3.436	2.716	1.026	3.079	3.608	3.377	6.104	2.426	2.391	1.458	0	0	-1.6	5.932	3.43	0	0	4.223	3.241	3.828	0	4.832	1.052	0
TaLTP1.6	0.426	-2.94	-0.86	3.142	0.554	4.131	3.197	4.86	3.505	0.551	0.861	0	0	0	0.567	0.861	0	0	-1.19	1.664	0.998	0	6.617	0.68	0
TaLTP1.7	1.758	-1.52	-0.5	4.5	1.619	4.84	3.962	5.406	4.563	1.551	2.061	0	0	0	5.583	2.136	0	0	3.049	3.371	1.312	-3.2	6.753	0.85	0
TaLTP1.8	7.78	9.12	7.587	8.116	8.101	8.101	8.114	8.816	8.192	7.245	5.808	0	0	-3.65	7.869	5.884	-2.96	0	6.575	6.898	6.626	-0.87	8.549	6.219	2.717
TaLTP1.9	6.692	10.23	6.571	7.045	7.147	7.426	6.771	8.741	6.979	6.347	5.148	0	0	0	7.192	5.579	0	-4.45	5.892	5.563	6.452	-1.56	8.162	6.035	3.66
TaLTP2.1	9.823	5.023	9.411	9.611	10.06	9.248	8.295	-0.02	9.293	9.137	1.468	0	0	-1.82	0.732	0	0	0	-1.54	-2.64	0	0	1.95	0	-1.29
TaLTP2.10	0.495	-2.85	0.302	-0.66	0.984	0	-0.28	0	0.39	0.375	0.988	1.844	0	2.373	-0.56	0.977	0	1.69	-0.66	3.32	-0.07	2.554	-0.24	-0.83	-0.67
TaLTP2.100	0	0	0	0	0	0	0	0	0	0	0	0	0	0	0	0	0	0	0	0	0	0	0	0	0
TaLTP2.101	0	0	0	0	0	-3.87	0	0	0	0	0	0	0	0	0	0	0	0	0	0	0	0	0	0	0
TaLTP2.102	0	0	0	-2.74	0	0	0	0	-3	0	0	0	0	0	-4.02	0	0	0	-4.81	-4.07	-3.21	-4.65	0	-2.19	0
TaLTP2.103	0	0	0	0	0	0	0	0	0	0	0	0	0	0	-5.43	0	0	0	0	0	0	0	0	0	0
TaLTP2.104	0	0	0	0	0	0	0	0	-1.86	0	0	-1.14	0	-0.87	0	-2.65	0	-1.02	0	0	0	0	0	0	0
TaLTP2.105	1.548	0	1.507	1.345	2.027	-0.92	1.734	-1.66	0.718	1.97	-0.04	2.106	0	4.156	0	0.098	0	3.517	0	-0.35	0	0	0	0	-3.17
TaLTP2.106	4.396	3.762	4.415	4.325	4.635	4.201	4.095	4.85	4.227	4.079	4.099	5.095	0	5.858	1.773	4.852	0	5.516	-1.34	2.725	-0.78	0	3.503	1.916	2.324
TaLTP2.107	0	-3.06	0	0	0	0	0	0	0	0	-2.88	-1.43	1.656	4.903	0	2.274	0	3.12	-2.89	2.785	0.254	0	0	2.787	0
TaLTP2.108	0	0	0	0	0	0	0	0	0	0	0	0	0	0	-1.89	0	0	0	0	0	0	0	0	0	0
TaLTP2.109	-1.65	5.079	-0.22	0.656	-0.98	0	0	-0.03	0	-1.82	-0.89	-3.35	8.545	-3.52	5.242	5.607	2.263	0	6.141	6.524	7.944	7.692	0.048	8.559	2.613
TaLTP2.11	-3.72	-0.81	0	0	-0.88	0	-3.05	0	-1.9	-3.29	-2.03	-1.76	0	-2.91	-1.52	0	0	0	0	-2.13	-3.49	-2.45	0	-3.6	-1.95
TaLTP2.110	0	0	0	0	0	0	0	0	0	0	0	0.321	0	0.176	0	0	0	-0.97	0	0	0	0	0	0	-2.88
TaLTP2.111	1.411	0	-0.61	1.76	0.101	-1.92	0.056	-2.66	0.718	-0.17	-0.84	1.603	0	2.792	0	-0.9	0	2.747	0	-1.94	0	0	0	0	0
TaLTP2.112	6.021	4.347	5.896	6.195	6.256	6.256	5.951	7.057	6.185	5.663	5.178	5.77	0	7.464	2.828	6.098	0	6.746	0.367	3.773	4.407	0	4.59	3.206	2.411
TaLTP2.113	0	0	0	0	0	0	0	0	-1.2	0	0.481	0.587	3.91	4.249	-1.88	3.035	0	2.938	-1.99	2.888	-2.84	0	0	4.163	0
TaLTP2.114	0	0	0	0	0	0	0	0	-0.94	-2.94	-3.35	0	0	0	2.008	0	0	0	0	-3.42	0	0	-3.25	0	-1.32
TaLTP2.115	0	0	0	0	0	0	0	0	0	0	0	-1.68	0	-1.24	0	0	0	-0.97	0	0	0	0	0	0	0
TaLTP2.116	0	0	0	0	0	0	0	0	0	0	0	3.158	0	2.805	0	-3.82	0	2.251	0	0	-3.66	0	0	0	0
TaLTP2.117	0	-2.81	0	0	0	0	0	1.155	0	0	0	0	0	0	-1.52	0	0	0	-1.04	0	0	0	0	0	0
TaLTP2.118	0	0	0	0	0	0	0	0	0	0	0	0	0	0	0	0	0	0	0	0	0	0	0	0	0
TaLTP2.119	0	0	0	0	0	0	0	0	0	0	0	5.983	0	5.575	0	-1.82	0	5.68	0	-3.86	-3.66	0	0	0	0
TaLTP2.12	9.059	7.326	8.592	8.974	9.332	8.614	7.721	-1.35	8.556	8.261	1.79	0	0	0	-0.12	-2.6	0	0	-0.54	0	0	0	2.312	0	0

TaLTP2.120	0	0	0	0	0	0	0	0	0	0	0	7.945	0	7.152	0	1.848	0	7.427	0	1.045	2.148	0	0	-2.76	0
TaLTP2.121	4.84	5.412	4.455	4.862	4.785	4.462	4.224	7.118	4.345	4.311	3.898	5.183	-2.56	6.465	3.803	5.466	-0.87	5.135	1.876	2.404	3.556	-1.1	3.708	1.221	4.009
TaLTP2.122	4.835	7.662	4.607	3.734	5.033	5.074	4.784	6.264	4.465	4.679	4.314	3.835	-3.62	0.096	2.837	7.567	-3.53	0.936	2.063	5.73	8.814	2.633	5.725	5.991	0
TaLTP2.123	0	-0.22	0	0	0	-2.81	0	-1.25	0	0	0	0	-3.46	0	0	0.46	0	0	-1.78	-0.82	0.378	0	0	0.272	0
TaLTP2.124	0	0	0	0	0	0	0	0	0	0	0	0	-1.71	0	0	0	0	0	0	0	0	0	0	-2.4	0
TaLTP2.125	0	0	0	0	0	0	0	0	0	0	0	-0.25	0	0	0	0	0	-4.06	0	0	4.624	0	0	0.447	0
TaLTP2.126	0	0	0	0	0	0	0	0	0	0	0	-0.47	0	-1.19	0	0	0	-0.59	0	0	0	0	0	0	0
TaLTP2.127	0	0	0	0	0	0	0	0	0	0	0	0	0	0	0	0	0	0	0	0	0	0	0	0	0
TaLTP2.128	0	0	0	0	0	0	0	0	0	0	0	-2.37	-2.53	0	0	0	0	-3.06	0	0	2.15	0	0	-0.99	0
TaLTP2.129	0	0	0	0	0	0	0	0	0	0	0	-3.17	0	0	0	0	0	0	0	0	-2.9	0	0	0	0
TaLTP2.13	1.824	6.36	2.067	2.179	1.544	2.456	2.367	4.709	2.78	1.745	0.971	-0.18	5.781	-0.05	3.95	4.969	0.046	1.669	3.857	5.121	6.685	5.647	3.595	5.932	-0.38
TaLTP2.130	0	0	0	0	0	0	0	0	0	0	0	-2.68	0	4.264	0	0	0.005	3.096	0	0	0	0	0	0	0
TaLTP2.131	0	0	0	0	0	0	0	0	0	0	0	0.063	0	0.315	0	0	0	2.067	0	0	0	0	0	0	0
TaLTP2.132	0	0	0	0	0	0	0	0	0	0	0	4.466	0	2.601	0	-2.07	0	2.756	0	-3.11	2.583	-1.28	0	-0.19	0
TaLTP2.133	0	0	0	0	0	0	0	0	0	0	0	3.743	0	2.121	-4.76	-1.44	0	2.346	0	-3.93	1.849	-2.72	0	-1.25	0
TaLTP2.134	0	0	0	0	0	0	0	0	0	0	0	4.637	0	2.803	-4.76	-1.19	0	3.474	0	0	2.415	-1.72	0	-1.03	0
TaLTP2.135	0	0	0	0	0	0	0	-2.52	0	0	0	-1.27	0	0	0	0	0	0	0	0	3.593	0	0	-2.69	0
TaLTP2.136	0	0	0	0	0	0	0	0	0	0	0	0	0	3.733	0	0	2.668	2.536	0	0	0	0	0	0	0
TaLTP2.137	0	0	0	0	0	0	0	0	0	0	0	-0.6	0	-1.2	0	0	0	0.622	0	0	0	0	0	0	0
TaLTP2.138	0	0	0	0	0	0	0	0	0	0	0	-0.66	0	0.257	0	-4.69	0	-0.22	0	-1.92	0	0	0	0	0
TaLTP2.139	0	0	0	0	0	0	0	0	0	0	0	-2.98	0	-3.23	0	-4.69	0	-2.28	0	-2.41	0	0	0	0	0
TaLTP2.14	7.199	2.667	6.686	6.596	7.486	6.418	6.197	0.462	7.265	6.488	-0.21	-0.68	0	-1.24	0	-2.6	0	0.255	0	0	-0.4	-0.78	-0.86	0	0
TaLTP2.140	0	0	0	0	0	0	0	0	0	0	0	-3.46	0	-3.23	0	0	0	-3.87	0	-2.73	0	0	0	0	0
TaLTP2.141	0	0	0	0	0	0	0	0	0	0	0	-0.43	0	-0.64	0	-3.69	0	-0.47	0	-0.92	0	0	0	0	0
TaLTP2.142	0	0	0	0	0	0	0	0	0	0	0	-3.99	0	0	0	0	0	0	0	0	0	0	0	0	0
TaLTP2.143	0	0	0	0	0	0	0	0	0	0	0	0	0	1.632	0	0	-0.58	-0.58	0	0	0	0	0	0	0
TaLTP2.144	0	0	0	0	0	0	0	0	0	0	0	0.61	0	0.167	0	-4.63	0	0.967	0	0	-7.49	0	0	0	0
TaLTP2.145	0	0	0	0	0	0	0	0	0	0	0	0	0	0	4.828	0	0	0	0	0	0	0	0	0	-2.71
TaLTP2.146	0.638	9.16	0.64	1.99	1.607	1.143	2.157	7.313	2.384	-2.25	0.233	0	2.289	0	6.57	1.603	6.999	0	5.57	2.022	0.141	0	3.348	-0.97	6.278
TaLTP2.147	0	-2.68	0	0	0	0	0	0	0	0	0	0	0	0	-2.4	0	0.906	0	0	0	0	0	0	0	6.629
TaLTP2.148	0	0	0	0	0	0	0	0	0	0	0	0	0	-2.06	3.403	0	-2.96	0	0	0	0	0	0	0	-1.32
TaLTP2.149	0	0	0	0	0	0	0	0	0	0	0	5.46	0	5.408	0	0	0	5.29	0	-3.86	0	0	0	-2.76	0
TaLTP2.15	0	0	0	0	0	0	0	0	0	0	0	4.828	0	2.973	0	-1.56	0	3.173	0	-3.91	0	0	0	0	0
TaLTP2.150	0	0	0	0	0	0	0	0	0	0	0	4.064	0	4.256	0	0	0	3.103	0	0	0	0	0	0	0
TaLTP2.151	4.877	3.142	4.428	4.565	4.243	4.188	4.202	6.536	4.372	4.206	3.836	5.001	0	5.322	2.688	5.129	0	4.512	0.797	2.266	2.815	-2.49	2.293	1.57	2.276
TaLTP2.152	-3.76	-2.38	0	0	0	0	0	-1.29	0	0	0	0	-1.17	0	-1.1	0	-2.4	0	0	-2.28	0.662	0	0	-0.76	0
TaLTP2.153	6.263	8.641	5.9	5.198	6.388	6.187	6.032	7.391	5.731	6.01	5.191	4.293	0	2.519	4.681	8.217	0	3.087	4.173	7.139	9.429	2.498	6.94	6.652	-0.53
TaLTP2.154	0	0	0	0	0	0	0	0	0	0	-1.33	0	0	0	0	0	0	0	0	3.627	0	2.862	0	-2.89	0
TaLTP2.155	0	0	0	0	0	-1.99	0	0	0	0	-2.91	-0.24	2.419	0.962	0	-0.38	0	0.578	-0.92	4.862	0	4.302	0	-2.89	0
TaLTP2.156	0	0	0	0	0	0	0	0	0	0	-1.33	0	-2.63	1.792	0	0.491	0	-0.2	-1.34	3.766	0	1.639	0	-2.89	0
TaLTP2.157	0	0	0	0	0	0	0	0	0	0	0	0	0	0	2.899	0	0	0	0	0	0	0	0	0	0
TaLTP2.158	0	0	0	0	0	0	0	0	0	0	0	0	0	0	2.244	0	0	0	0	0	0	0	0	0	0
TaLTP2.159	0	0	0	0	0	0	0	0	0	0	0	0	-1.55	0	0	0	0	0	1.493	0	0	0	-0.76	-1.31	0
TaLTP2.16	0	0	0	0	0	0	0	0	0	0	0	3.989	0	2.682	0	-0.05	0	2.436	0	-2.9	0	0	0	0	-4.11
TaLTP2.160	0	0	0	0	0	0	0	0	0	0	0	0	-2.04	0	0	0	0	0	1.452	0	0	0	-0.76	-1.31	0
TaLTP2.161	5.261	2.936	5.051	4.419	5.41	3.841	4.493	7.436	4.639	4.873	4.321	3.945	0	5.117	3.375	5.076	0	4.419	2.041	1.924	2.464	0	2.85	1.092	2.844
TaLTP2.162	5.304	8.009	4.881	4.433	5.495	5.13	4.677	6.086	4.704	5	4.209	3.677	0	0.458	2.602	7.281	0	1.897	2.175	5.297	8.458	1.807	6.455	5.875	-2.4
TaLTP2.163	-1.19	0	-3.11	-2.69	-2.12	-2.86	-3.2	0	-1.95	0	0	-3.94	-3.51	-3.12	-1.38	-2.26	0	0	-2.25	-1.56	-1.09	-3.67	0	-2.2	0
TaLTP2.164	0	0	0	0	0	0	0	0	0	0	0	-2.76	0.543	-0.44	0	-4.24	0	-1.89	0	-3.28	0	0	0	-3.19	0
TaLTP2.165	0	0	0	0	-2.14	0	0	0	0	0	0	2.471	-2.53	-2.14	0	-2.28	0	-0.89	0	-2.9	6.415	0	0	0.784	0
TaLTP2.166	0	0	0	0	0	0	0	0	0	0	0	-3.62	0	0	0	0	0	0	0	0	0	0	0	0	0
TaLTP2.167	0	0	0	0	0	0	0	0	0	0	0	0	0	-3.97	0	0	0	0	0	-2.14	0	0	0	0	0
TaLTP2.168	2.071	-2.77	0.64	3.727	1.607	1.92	3.116	-0.07	3.18	1.067	-1.57	0	1.704	-2.87	7.333	-0.65	7.109	0	4.669	0.322	-2.44	-1.41	1.603	-0.56	8.484
TaLTP2.169	0	0	0	0	0	0	0	0	0	0	0	-0.46	-0.04	0.377	0	-1.97	0	-0.69	0	5.202	0	4.067	0	0	0
TaLTP2.17	0	0	0	0	0	-2.36	0	0	0	0	0	7.722	0	7.13	-3.19	1.362	0	7.099	0	-2.37	1.087	0	-1.2	-0.46	-1.59
TaLTP2.170	0	0	0	0	0	0	0	0	0	0	0	0.651	0.182	-2.21	0	0	0	-0.69	-0.6	6.197	0	5.836	0	-1.31	0
TaLTP2.171	0	0	0	0	0	0	0	0	0	0	0	0.651	1.462	3.825	0	1.787	-2.52	3.637	-0.92	6.818	-1.78	4.466	0	0.279	0
TaLTP2.172	0	0	0	0	0	0	0	0	0	0	0	0	0	0	0	0	0	0	0	0	0	0	0	0	0
TaLTP2.173	0	0	0	0	0	0	0	0	0	0	0	0	0	0	-2.88	0	0	0	0	0	0	0	0	0	0
TaLTP2.174	0	0	0	0	0	0	0	0	0	0	0	0	0	0	1.847	0	0	0	0	0	0	0	0	0	0
TaLTP2.175	1.558	5.138	1.593	2.565	2.654	2.118	0.653	-0.72	1.529	0.404	0	0	0	0	7.926	-2.56	0	0	3.208	0	0	0	0.408	0	-1.25
TaLTP2.176	1.052	6.663	2.001	1.901	1.867	1.546	0.834	3.562	0.979	-1.22	-2.53	0	-2.25	0	5.382	1.302	1.183	0	2.749	0.532	-2.4	-2.36	1.645	-1.52	6.139
TaLTP2.177	0	0	0	0	0	0	0	0	0	0	0	-1.46	-0.04	4.573	0	2.202	0	3.888	-1.92	5.127	0	3.604	0	-0.57	0
TaLTP2.178	0	0	0	0	0	0	0	0	0	0	0	0	0	0	2.074	0	0	0	0	0	0	0	0	0	0
TaLTP2.179	0	0	0	0	0	0	0	0	0	0	0	0	0	0	0.737	0	0	0	0	0	0	0	0	0	0
TaLTP2.18	0	0	0	0	0	0	0	0	0	0	0	0	0	0	0	0	0	0	0	0	0	0	-3.55	0	0
TaLTP2.180	-2.16	10.29	-3.52	-4.09	0	-3.28	-2.61	1.927	-3.34	0	0	0	0	0	-4.12	-4.26	-2.84	0	0	0	0	0	5.344	0	0
TaLTP2.181	0	9.68	0	-4.15	0	0	0	0.584	0	0	-2.31	0	0	0	0	0	0	0	0	0	0	0	6.454	0	0
TaLTP2.182	4.358	0.293	4.11	3.173	4.421	3.985	2.717	5.391	3.632	3.811	2.959	-0.03	0	-0.21	2.768	4.208	-3.1	-0.5	1.139	3.821	1.289	0.565	1.814	1.786	-3.78
TaLTP2.183	-3.15	6.623	-3.51	-2.07	-1.51	-1.68	-3.59	-0.87	-3.32	-2.76	-1.91	-4.34	-2.92	0	-3.11	0	-2.24	0	-2.66	0	0	0	3.583	0	0
TaLTP2.184	0	2.707	0	0	0	-3.05	0	0	0	0	-4.01	0	0	0	0	0	0	0	0	0	0	0	1.048	0	0
TaLTP2.185	6.718	4.219	6.662	5.941	6.73	6.237	5.223	6.767	6.047	6.245	4.805	0	0	-2.79	5.552	5.577	0	-3.67	3.625	4.741	6.36	-2.34	4.19	4.642	0

TaLTP2.186	0	6.153	0	0	0	0	0	-3.73	0	0	-2.92	0	0	0	0	0	0	0	0	0	0	0	5.946	0	0
TaLTP2.187	0	9.814	0	0	0	-3.34	0	0.742	-2.81	0	-3.31	0	0	0	0	0	-3.91	0	0	0	0	0	5.445	0	0
TaLTP2.188	0	10.48	0	-2.12	0	-1.73	0	1.998	-2.79	-3.81	0	0	0	0	0	0	-2.88	0	0	0	0	0	5.452	0	0
TaLTP2.189	0	-1.44	0	0	0	0	0	0	0	0	0	0	0	1.321	0	0	0	-0.6	0	-0.94	0	0	0	0	0
TaLTP2.19	2.223	1.934	2.225	1.213	2.385	1.335	0.795	-0.39	1.943	1.248	-2.57	1.865	0	4.534	0	2.109	0	3.525	-0.99	2.876	0	4.415	0.323	-2.56	0
TaLTP2.190																									
TaLTP2.191	0	0	0	0	0	0	0	0	0	0	0	4.989	0	5.361	0	-0.65	0	4.497	0	-0.88	0.516	-2.47	0	0	0
TaLTP2.192	0	0	0	0	0	0	0	0	0	0	-3.62	6.77	0	6.075	0	-0.19	0	5.449	0	0	-1.16	0	0	0	0
TaLTP2.193	0	0	0	0	0	0	0	0	0	0	-3.04	5.865	0	4.731	0	1.722	0	4.354	0	-2.1	-2.9	0	0	0	0
TaLTP2.194	0	0	0	0	0	0	0	0	0	0	-3.04	5.865	0	4.731	0	1.722	0	4.354	0	-2.1	-2.9	0	0	0	0
TaLTP2.195	0	0	0	0	0	0	0	0	0	0	-2.64	6.123	0	7.152	0	0.027	0	6.197	0	-3.71	2.624	0	-3.55	-1.61	-0.75
TaLTP2.196	0	0	0	0	0	0	0	0	0	0	0	6.406	0	7.472	0	-2.67	0	6.273	0	0	0.955	0	0	0	-3.92
TaLTP2.197	0	0	0	0	0	0	0	0	0	0	0	0	0	3.183	0	0	0	0.074	0	-0.94	0	-1.68	0	0	-3.17
TaLTP2.198	6.322	0	6.162	5.984	6.74	5.717	5.379	-3.53	6.115	5.859	-1.72	0	0	-2.44	0	0	0	0	0	0	0	0	-2.63	0	0
TaLTP2.199	0	-0.68	0	0	-1.9	0	0	0	0	0	0	0	0	0	0	-0.98	1.228	0	0	1.652	-2.36	0	0	0	0
TaLTP2.2	1.894	8.782	2.26	2.294	2.807	3.758	3.197	6.214	2.569	2.108	2.404	0	5.031	0	4.695	4.881	-1.95	0	5.21	5.644	5.56	5.621	4.213	5.455	-0.12
TaLTP2.20	4.636	4.408	4.055	4.367	4.864	4.642	3.391	2.635	4.424	4.1	2.01	6.378	-2.3	8.766	0	5.14	0	8.174	0.39	6.738	1.397	6.794	3.95	1.248	0
TaLTP2.200	0	0	0	0	0	0	0	0	0	0	0	0	0	0	0	0	0	0	0	0	0	0	0	0	0
TaLTP2.201	0	0	0	-3.51	0	0	0	0	0	0	0	6.703	0	6.167	0	0	-3.23	5.757	0	0	0	0	0	0	0
TaLTP2.202	0	0	0	0	0	0	0	0	0	0	0	5.146	0	3.562	0	-2.65	-2.23	3.839	0	0	0	0	0	0	-2.9
TaLTP2.203	0	0	0	0	0	0	0	0	0	0	0	6.61	0	4.619	0	-1.07	-2.23	5.017	0	0	0	0	0	0	0
TaLTP2.204	0	0	0	0	0	0	0	0	0	0	0	5.407	0	3.767	0	-3.65	-3.23	4.284	0	0	0	0	0	0	0
TaLTP2.205	0	0	0	0	0	0	0	0	0	0	0	5.57	0	3.78	0	0	0	4.342	0	0	0	0	0	0	0
TaLTP2.206	0	0	0	-3.51	0	0	0	0	0	0	0	6.307	0	4.494	0	0	0	4.305	0	0	0	0	0	0	0
TaLTP2.207	0	0	0	0	0	0	0	0	0	0	0	7.358	0	6.507	0	2.407	0	6.234	0	0.538	1.495	-1.91	-3.55	-2.03	-2.92
TaLTP2.208	0	0	0	0	0	0	0	0	0	0	-3.64	7.08	0	7.266	0	2.554	0	6.584	0	0.165	1.168	-3.49	0	-1.61	0
TaLTP2.209	0	0	0	0	0	0	0	0	0	0	0	6.334	0	5.721	0	0.334	0	5.567	0	-2.97	-1.18	0	0	-2.61	-3.92
TaLTP2.21	1.316	6.192	0.64	0.99	1.829	0.92	-0.43	1.072	1.136	-2.25	-2.57	4.367	-2.3	5.139	-1.48	3.564	0	4.11	-1.58	4.333	-2.44	3.842	0.975	-2.56	0
TaLTP2.210	0	0	0	0	0	0	0	0	0	0	-3.64	6.781	0	6.457	0	2.043	0	6.278	0	-0.32	0.402	-3.49	0	-2.61	-3.92
TaLTP2.211	0	0	0	0	0	0	0	0	0	0	0	5.061	0	5.244	0	0.63	0	4.841	0	-1.65	-0.6	0	0	0	0
TaLTP2.212	-2.79	0	0	0	-2.09	0	-1.13	-1.51	-1.97	0	-2.7	2.162	0	2.596	0	1.047	0	3.205	0	1.206	-1.56	0	0	-2.68	0
TaLTP2.213	0	0	0	0	0	0	0	0	0	0	0	2.178	0	0.63	0	-2.34	0	1.491	0	-1.37	0	0	0	0	0
TaLTP2.214	6.457	0	6.112	6.268	6.903	6.097	5.539	-3.52	6.243	6.079	-0.9	0	0	0	0	0	0	0	0	0	0	0	-1.02	0	0
TaLTP2.215	0	0	0	0	0	0	0	0	0	0	0	0	0	0	0	0	0	0	0	0	0	0	0	0	0
TaLTP2.216	0	0	0	0	0	0	0	0	0	0	0	6.856	0	6.949	0	-2.67	0	6.361	0	0	0	0	0	0	0
TaLTP2.217	0	0	0	0	0	0	0	0	0	0	0	1.12	0	1.701	0	0	0	2.124	0	0	0	0	0	0	0
TaLTP2.218	0	0	0	0	0	0	0	0	0	0	0	3.64	0	2.217	0	0	0	1.534	0	0	0	0	0	0	0
TaLTP2.219	0	0	0	0	0	0	0	0	0	0	0	7.337	0	7.628	0	-0.67	0	6.975	0	0	-1.92	0	0	0	0
TaLTP2.22	0	0	0	0	0	0	0	0	0	0	-2.15	0	0	-0.85	0	0	0	0	0	0	0	0	0	0	0
TaLTP2.220	0	0	0	0	0	0	0	0	0	0	0	0.407	0	3.126	0	0	0	0.891	0	0	0	0	0	0	0
TaLTP2.221	0	0	0	0	0	0	0	0	0	0	0	-0.4	0	0	0	0	0	-2.8	0	0	0	0	0	0	0
TaLTP2.222	0	0	0	0	0	0	0	0	0	0	0	0	0	0	0	0	-0.14	0	0	0	0	0	0	0	7.709
TaLTP2.223	2.486	8.852	2.755	3.405	3.231	2.036	1.987	7.223	3.384	1.833	0	0	0.704	0	7.875	1.525	5.27	0	6.902	3.077	2.256	1.295	3.07	0	7.716
TaLTP2.224	3.447	5.881	2.698	3.422	2.565	2.63	2.11	-0.26	2.999	2.25	-2.44	0	0	0	7.538	-1.52	0	0	2.46	0	0.008	0	1.732	-0.11	0.666
TaLTP2.225	2.438	4.092	3.502	5.012	3.813	4.392	4.747	4.98	4.911	2.637	3.828	1.045	5.895	1.24	8.568	6.898	3.106	0.961	7.484	7.857	8.743	6.312	7.012	8.184	3.495
TaLTP2.226	1.466	3.911	2.667	4.409	3.369	3.447	4.414	2.291	3.752	1.265	2.714	0	3.453	0	6.631	6.006	4.325	0	5.305	6.108	7.437	3.628	5.478	6.802	2.069
TaLTP2.227	0	-2.58	0	0	0	0	0	0	-3.41	0	-1.89	0	3.822	0	0.608	0.936	0	-3.36	1.187	3.27	5.213	4.529	-1.9	6.166	-0.82
TaLTP2.228	0	-2.58	0	0	0	0	0	0	-3.41	0	-1.89	0	3.822	0	0.608	0.936	0	-3.36	1.187	3.27	5.213	4.529	-1.9	6.166	-0.82
TaLTP2.229	0	-5.39	0	0	0	0	0	0	0	0	-3.22	0	3.601	0	-0.24	0.263	0	-3.36	0.357	2.369	5.051	4.083	-1.8	5.871	-2.52
TaLTP2.23	-2.72	-1.81	0	0	-4.34	0	-3.05	0	0	0	0	-1.18	0	-0.1	0	-0.68	0	-0.33	-2.62	4.521	0	3.761	-1.53	0	0
TaLTP2.230	0	-5.39	0	0	0	0	0	0	0	0	-1.22	0	3.969	0	0.111	0.724	0	-3.36	0.969	2.581	5.065	4.619	-2.25	6.071	-2.52
TaLTP2.231	0	0	0	0	0	0	0	0	0	0	0	2.466	0	1.612	0	-3.17	0	2.835	0	0	0	0	0	0	0
TaLTP2.232	0	0	0	0	0	0	0	0	0	0	0	-0.89	0	-0.86	0	0	0	-0.93	0	0	0	0	0	0	0
TaLTP2.233	0	0	0	0	0	0	0	0	0	0	0	-3.97	0	-3.16	0	0	0	-1.75	0	0	0	0	0	0	0
TaLTP2.234	0	0	0	0	0	0	0	0	0	0	0	-4.47	0	0	0	0	0	-4.62	0	0	0	0	0	0	0
TaLTP2.235	0	0	0	0	0	0	0	0	0	0	0	0	0	0	0	0	0	0	0	0	0	0	0	0	0
TaLTP2.236	0	0	0	0	0	0	0	0	0	0	0	-5.27	0	0.935	0	0	-4.17	0.362	0	0	0	0	0	0	0
TaLTP2.237	0	0	0	0	0	0	0	0	0	0	0	4.089	-3.53	0.816	0	-3.86	0	1.559	0	-3.9	7.169	0	-3.74	2.913	0
TaLTP2.238	0	0	0	0	0	0	0	0	0	0	0	-1.72	0.511	0	0	0	0	-1.02	0	-2.68	0	0	0	-2.56	0
TaLTP2.239	0	0	0	0	0	0	0	0	0	0	0	0	0	0	0	0	0	0	0	0	0	0	0	0	0
TaLTP2.24	-0.18	7.249	0.26	0.916	0	0.549	0.782	2.839	1.78	0.745	-0.77	0	5	0	3.522	4.707	0	0	3.263	5.418	6.282	5.608	2.691	5.192	-2.71
TaLTP2.240	0	0	0	0	0	0	0	0	0	0	0	0	0.615	-4.59	0	-4.3	0	-3.54	0	-0.43	-4.14	0	0	-1.07	0
TaLTP2.241	-2.39	-1.99	-2.86	0.082	-0.6	-0.25	0.11	0.956	0.78	-1.56	-0.58	-2.58	3.86	-3.38	4.659	3.752	-0.58	-5.68	3.869	4.109	5.966	2.415	2.985	6.119	-0.38
TaLTP2.242	-5.23	0	0	-2.15	-2.98	-2.7	-1.81	-2.03	-1.83	-3.24	-2.63	-3.35	2.678	0.129	3.781	2.088	-0.12	-0.84	2.636	2.976	3.777	2.529	1.402	4.68	-0.87
TaLTP2.243	0	0	0	-2.74	0	-3.87	0	0	-3	0	0	0	0	0	-4.02	0	0	0	-4.81	-4.07	-3.21	-4.65	0	-2.19	0
TaLTP2.244	0	0	0	0	0	0	0	0	0	0	0	-3.27	0	0.388	0	0	0	0.587	0	0	0	0	0	0	0
TaLTP2.245	0	0	0	0	0	0	0	0	0	0	0	0	0	1.416	0	0	0	-0.85	0	0	0	0	0	0	0
TaLTP2.246	0	0	0	0	0	0	0	0	0	0	0	0	0	0	0	0	0	0	0	0	0	0	0	0	0
TaLTP2.247	0	0	0	0	0	0	0	0	0	0	0	0	0	0	0	0	0	0	0	0	0	0	0	0	0
TaLTP2.248	0	0	0	0	0	0	0	0	0	0	0	0	0	0	0	0	0	-4.08	0	0	0	0	0	0	0
TaLTP2.249	0	0	0	0	0	0	0	0	0	0	0	0	0	-1.1	0	0	0	-2.91	0	0	0	0	0	0	0
TaLTP2.25	9.061	1.919	8.717	9.323	9.368	8.252	7.996	0.462	9.119	8.2	1.375	0	0	0	-2.44	0	0	0	0	0	0	0	1.365	0	0

TaLTP2.250	0	0	0	0	0	0	0	0	0	0	0	0	0	0	0	0	0	0	0	0	0	0	0	0	0
TaLTP2.251	0	0	0	0	0	0	0	0	0	0	0	0	0	0	0	0	0	0	0	0	0	0	0	0	0
TaLTP2.252	0	0	0	0	0	0	0	0	0	0	0	0	0	0	0	0	0	0	0	0	0	0	0	0	0
TaLTP2.253	-5.23	0	0	-2.15	-2.98	-2.7	-1.81	-2.03	-1.83	-3.24	-2.63	-3.35	2.678	0.129	3.781	2.088	-0.12	-0.84	2.636	2.976	3.777	2.529	1.402	4.68	-0.87
TaLTP2.254	0	0	0	0	0	0	0	0	0	0	0	-3.05	0.939	3.643	0	0.769	0	3.215	1.017	5.763	0	4.869	0	-1.72	0
TaLTP2.255	0	0	0	0	0	0	0	0	0	0	-2.5	0	-2.63	1.436	0	0.147	0	0.527	0.816	3.602	0	2.105	0	0	0
TaLTP2.256	0	0	0	0	0	0	0	0	0	0	0	0	0	0	0	0	0	-2.39	0	0	0	0	0	0	0
TaLTP2.257	0	0	0	0	0	0	0	0	0	0	0	0	0	0	0	0	0	0	0	0	0	0	0	0	0
TaLTP2.258	0	0	0	0	0	0	0	0	0	0	0	0	0	0	0	0	0	0	0	0	0	0	0	0	0
TaLTP2.259	0	0	0	0	0	0	0	0	0	0	0	-5.27	0	-5.44	0	0	0	-1.16	0	0	0	0	0	0	0
TaLTP2.26	-3.96	0	0	0	0	-0.74	0	0	-1.13	0	0	0	0	0	0	0	0	0	0	0	-1.56	0	-3.92	-2.4	0
TaLTP2.260	0	0	0	0	0	0	0	0	0	0	0	0.199	-0.51	3.495	0	0.513	0	2.778	-0.05	5.782	0	5.118	0	-2.57	0
TaLTP2.261	0	0	0	0	0	0	0	0	0	0	0	0	0	0	0	0	0	0	0	0	0	0	0	0	0
TaLTP2.262	0	-3.39	0	0	-3.15	-2.87	0	-3.03	-3	0	-1.63	0	7.453	-4.1	4.755	4.707	-0.09	0	5.565	5.664	6.514	6.302	-1.54	7.781	2.91
TaLTP2.263	-5.23	0	0	-2.15	-2.98	-2.7	-1.81	-2.03	-1.83	-3.24	-2.63	-3.35	2.678	0.129	3.781	2.088	-0.12	-0.84	2.636	2.976	3.777	2.529	1.402	4.68	-0.87
TaLTP2.264	0	0	0	0	0	0	0	0	0	0	0	0	0	1.159	0	0	-4.17	0.362	0	0	0	0	0	0	0
TaLTP2.265	0	0	0	0	0	0	0	0	0	0	0	0	0	0	0	0	0	0	0	0	0	0	0	0	0
TaLTP2.266	0	0	0	0	0	0	0	0	0	0	0	-3.97	0	0	0	0	0	-4.08	0	0	0	0	0	0	0
TaLTP2.267	-2.39	-1.99	-2.86	0.082	-0.6	-0.25	0.11	0.956	0.78	-1.56	-0.58	-2.58	3.86	-3.38	4.659	3.752	-0.58	-5.68	3.869	4.109	5.966	2.415	2.985	6.119	-0.38
TaLTP2.268	-5.23	0	0	-2.15	-2.98	-2.7	-1.81	-2.03	-1.83	-3.24	-2.63	-3.35	2.678	0.129	3.781	2.088	-0.12	-0.84	2.636	2.976	3.777	2.529	1.402	4.68	-0.87
TaLTP2.269	0	0	0	0	0	0	0	0	0	0	0	0	0	0	0	0	0	0	0	0	0	0	0	0	0
TaLTP2.27	0	0	0	0	0	0	0	0	0	0	0	6.903	0	5.144	0	1.958	0	5.618	0	0.051	0	0	0	0	0
TaLTP2.270	0	0	0	0	0	0	0	0	0	0	0	0	0	0	0	0	0	0	0	0	0	0	0	0	0
TaLTP2.271	0	0	0	0	0	0	0	0	0	0	0	0	0	0	0	0	0	0	0	0	0	0	0	0	0
TaLTP2.272	0	0	0	0	0	0	0	0	0	0	0	0	0	0	0	0	0	0	0	0	0	0	0	0	0
TaLTP2.273	0	0	0	0	0	0	0	0	0	0	0	1.624	0	-0.08	0	0	0	1.058	0	0	0	0	0	0	0
TaLTP2.274	0	0	0	0	0	0	0	0	0	0	0	1.572	0	0.611	0	-4.77	0	1.662	0	0	0	0	0	0	0
TaLTP2.28	0	0	0	0	0	0	0	0	0	0	0	6.242	0	4.732	0	-0.22	-1.96	5.067	0	-2.42	0	0	0	0	0
TaLTP2.29	0	0	0	0	0	0	0	-2.95	0	0	0	6.896	0	5.618	0	0.406	0	6.09	0	-2.21	0	0	0	0	0
TaLTP2.3	-0.01	0	-2.26	0.486	0.053	-0.01	-2.35	-3.78	-0.28	-1.2	0	0	0	-0.47	0	0	0	0	0	0	-0.51	0	-2.29	-1.94	0
TaLTP2.30	-3.21	0	0	0	0	0	0	0	0	0	0	6.392	0	5.68	0	0.574	0	5.985	0	-3.27	-3.05	0	0	0	0
TaLTP2.31	0	0	0	0	0	0	0	0	0	0	0	6.456	0	5.346	0	1.014	0	5.952	0	-2.27	0	0	0	0	0
TaLTP2.32	0	0	0	0	0	0	0	0	0	0	0	0	0	0	0	0	0	0	0	0	-1.18	0	-3.55	0	0
TaLTP2.33	1.424	3.234	1.377	2.751	0.88	1.816	0.532	0.612	2.01	1.586	-0.25	6.103	0.289	8.131	0	3.688	0	7.862	1.494	6.431	1.971	6.671	2.744	0.441	-2.91
TaLTP2.34	2.573	6.136	2.913	2.405	3.151	3.09	1.688	2.136	2.891	2.833	0.595	6.1	0	7.147	-0.48	4.285	0	6.443	1.006	4.944	2.643	4.238	2.515	0.611	-0.33
TaLTP2.35	0.247	2.613	0.565	1.658	1.671	0.579	-0.77	-0.15	-1.93	0.259	0.151	3.41	0	3.201	-1.56	0.736	0	1.757	0	2.472	-2.53	0.317	0.894	0	-2.99
TaLTP2.36	0	-0.52	0	0	0	0	0	-2.47	0	0	-1.66	-2.8	0	-1.95	0	-2.72	0	0	0	-0.76	0	0	0	0	0
TaLTP2.37	0	0	0	0	0	0	0	0	0	0	0	0	0	0	0	0	0	0	0	0	0	0	0	0	0
TaLTP2.38	0	0	0	0	-3.92	0	0	0	0	0	0	0	-0.29	2.425	0	0	0	-0.49	-0.56	3.329	0	-1.96	0	-2.14	0
TaLTP2.39	0	0	0	0	0	0	0	0	-2.65	0	0	4.723	0	3.88	0	0	0	3.574	0	0	0	0	0	0	0
TaLTP2.4	0	0	0	0	0	0	0	0	0	0	0	6.917	0	5.548	0	1.497	0	5.98	0	0.609	0	0	-3.75	-3.82	-4.12
TaLTP2.40	0	0	0	0	0	0	0	0	0	0	0	7.808	0	6.442	0	-1.63	0	7.062	0	0	0	0.248	0	-1.99	0
TaLTP2.41	0	0	0	0	0	0	-4.54	-3.98	0	0	-4.17	0	1.783	0	0	2.834	0	-3.42	-4.19	0.657	4.246	0	-2.26	5.405	0
TaLTP2.42	0	0	0	0	0	0	0	0	0	0	0	-0.24	0	-1.62	0	-0.16	0	-0.42	0	4.449	0	4.041	0	-2.89	0
TaLTP2.43	0	0	0	0	0	0	0	0	0	0	-1.91	1.858	-2.63	2.114	0	-1.38	0	1.748	-1.92	5.521	0	5.471	0	-2.89	0
TaLTP2.44	0	0	0	0	0	0	0	0	0	0	-2.91	-0.46	-0.3	1.251	0	1.119	0	-0.01	0.079	6.55	0	5.978	-2.82	-1.31	0
TaLTP2.45	0	0	0	0	-1.67	0.981	0	-1.04	-1.58	-1.96	0	0	0	0	-2.15	-2.32	0	0	0	-2.35	3.648	0	-2.15	-1.23	0
TaLTP2.46	0	0	0	0	0	0	0	0	0	0	0	0	0	0	0	0	0	0	0	0	0	0	0	-0.03	0
TaLTP2.47	0	0	0	0	0	0	0	0	0	0	0	0	-5.34	0	0	0	0	0	0	0	0	0	0	0.455	0
TaLTP2.48	0	0	0	0	0	0	0	0	0	0	0	0	-5.34	0	0	0	0	0	0	0	0	0	0	-0.24	0
TaLTP2.49	0	0	0	0	0	0	0	0	0	0	0	0	0	0	0	0	0	0	0	0	0	0	0	0.117	0
TaLTP2.5	0	0	0	0	0	0	0	-3.16	0	0	-3.35	6.474	0	6.071	-3.24	0.072	-2.96	6.075	0	-1.84	1.832	0	0	0.265	-1.06
TaLTP2.50	0	0	0	0	0	0	0	0	0	0	0	0	-5.34	0	0	0	0	0	0	0	0	0	0	-0.24	0
TaLTP2.51	0	0	0	0	0	0	0	0	0	0	0	0	0	0	0	0	0	0	0	0	0	0	0	0.117	0
TaLTP2.52	0	0	0	0	0	0	0	0	0	0	0	0	0	0	0	0	0	0	0	0	0	0	0	1.444	0
TaLTP2.53	0	0	0	0	0	0	0	0	0	0	0	0	0	0	0	0	0	0	0	0	0	0	0	0	0
TaLTP2.54	0	0	0	0	0	0	0	0	0	0	0	0	0	0	0	0	0	0	0	0	0	0	0	-1.61	0
TaLTP2.55	0	0	0	0	0	0	0	0	0	0	0	0	-5.34	0	0	0	0	0	0	0	0	0	0	-0.24	0
TaLTP2.56	0	0	0	0	0	0	0	0	0	0	-3.64	0	0	0	0	0	0	0	0	0	0	0	0	0	0
TaLTP2.57	0	0	0	0	0	0	0	0	0	0	-3.64	0	0	0	0	0	0	0	0	0	0	0	0	-0.09	0
TaLTP2.58	0	0	0	0	0	0	0	0	0	0	0	0	0	0	0	0	0	0	0	0	0	0	0	-0.61	0
TaLTP2.59	0	0	0	0	0	0	0	0	0	0	0	0	0	0	0.52	0	0	0	0	0	0	0	0	0	0
TaLTP2.6	0	0	0	-2.13	0	0	-2.59	0	0	0	0	5.299	0	4.909	0	-1.65	0	5.093	0	-1.27	0	0	0	-2.16	0
TaLTP2.60	0	0	0	0	0	0	0	0	0	0	0	0	0	0	0	0	0	0	0	0	0	0	0	0	1.535
TaLTP2.61	0	0	0	0	0	0	0	0	0	0	0	0	0	0	0	0	0	0	0	0	0	0	0	0	0
TaLTP2.62	0	0	0	0	0	0	0	0	0	0	0	5.915	0	4.682	0	3.068	0	5.119	-3.61	0.579	-1.88	1.869	-3.5	-1.99	0
TaLTP2.63	0	0	0	0	0	0	0	0	0	0	0	7.686	0	6.441	0	-0.17	0	6.696	0	0	-3.46	0	0	0	0
TaLTP2.64	0	0	0	0	0	0	-4.54	0	0	0	-4.17	0	4.615	0	-4.05	2.593	0	-4.42	-4.19	-1.32	4.907	-3.03	-1.9	6.43	0
TaLTP2.65	0	0	0	0	0	0	0	0	0	0	-1.91	1.343	1.462	3.118	0	1.732	0	3.721	1.779	6.313	0	5.919	0	-1.31	0
TaLTP2.66	0	0	0	0	0	0	0	0	0	0	0	0	0	0	0	0	0	0	0	0	0	0	0	0	0
TaLTP2.67	0	0	0	0	0	0	0	0	0	0	0	-3.05	0.939	3.643	0	0.769	0	3.215	1.017	5.763	0	4.869	0	-1.72	0
TaLTP2.68	0	0	0	0	0	0	0	0	0	0	-3.18	0	0	1.65	0	0.147	0	-0.81	-0.81	2.446	0	1.662	0	0	0

TaLTP2.69	0	0	0	0	0	0	0	0	0	0	0	0	0	1.436	0	0.354	0	-0.92	0.249	2.761	0	1.593	0	0	0
TaLTP2.7	0	0	0	0	0	0	0	0	0	0	0	0	0	0	0	0	0	0	0	0	0	0	0	0	0
TaLTP2.70	0	0	0	0	0	0	0	0	0	0	-3.18	0	0	1.65	0	0.147	0	-0.81	-0.81	2.446	0	1.662	0	0	0
TaLTP2.71	0	0	0	0	0	0	0	0	0	0	-5.56	-6.64	0	0	0	0	0	0	-6.56	0	0	-4.82	0	0	0
TaLTP2.72	0	0	0	0	0	0	0	0	0	0	0	0	0	0	-2.6	0	0	0	0	0	0	0	0	0	0
TaLTP2.73	-3.54	0	0	0	-2.88	-1.61	-1.95	-1.37	0	-2.13	-3.56	0	0	0	-2.45	-1.59	0	0	0	-2.63	0.579	0	-3.46	-1.94	0
TaLTP2.74	0	0	0	0	0	0	0	0	0	0	0	0	0	1.328	0	0	0	0	0	0	0	0	0	0	0
TaLTP2.75	0	0	0	0	0	0	0	2.655	0	0	0	0	0	0	-2.44	0	0	0	0.271	0	0	0	0	0	0
TaLTP2.76	0	0	0	0	0	0	0	0	0	0	0	0	0	0	0	0	0	0	0	0	0	0	0	0	3.941
TaLTP2.77	0	0	0	0	0	0	0	0	0	0	0	8.475	0	6.609	0	4.562	0	7.064	0	-3.67	0.536	2.478	0	-1.25	0
TaLTP2.78	0	-3.33	0	0	0	0	0	-3.98	0	0	0	0	5.619	-2.48	-1.47	0.335	-3.77	0	-3.19	1.582	5.279	-0.33	-2.07	6.459	0
TaLTP2.79	0	0	0	0	0	0	0	0	0	0	-1.33	1.121	0.182	0.792	0	1.555	0	0.315	-0.34	6.776	0	5.926	0	-0.89	0
TaLTP2.8	5.022	5.883	4.578	4.803	5.32	5.112	3.655	3.851	4.424	4.559	1.818	6.479	-0.71	9.044	-0.89	5.408	0	8.134	2.755	6.679	1.671	6.941	3.515	2.727	-1.91
TaLTP2.80	0	0	0	0	0	0	0	0	0	0	0	0.474	-1.63	1.478	0	0.955	0	1.721	0.079	5.512	0	4.925	0	-1.72	0
TaLTP2.81	0	0	0	0	0	0	0	0	0	0	0	0.858	0	-0.62	0	-1.38	0	-1.01	-1.34	5.137	0	5.297	0	0	0
TaLTP2.82	0	0	-1.63	-1.31	-1.67	0	-1.7	0.542	-1.58	-1.96	0	0	0	0	-2.15	0.263	0	0	0	0	3.023	0	0	-1.23	0
TaLTP2.83	0	0	0	0	0	0	0	0	0	0	0	0	0	0	0	0	0	0	0	0	0	0	0	0	3.434
TaLTP2.84	0	0	0	0	0	0	0	0	0	0	0	0	0	-3.52	1.474	-3.26	0	0	-1.64	0	4.006	0	-3.12	4.193	-2.52
TaLTP2.85	0	0	0	0	0	0	0	0	0	0	0	0	0	0	-3.11	0	0	0	-2.23	0	3.41	0	0	1.333	0
TaLTP2.86	-1.06	-1.14	-1.91	0	-1.94	-1.62	0	-1.35	0	0	0	-1.09	-2.25	0.176	0	0	-2.14	-0.55	0	0	0	0	0	0	0
TaLTP2.87	4.899	3.304	4.694	5.526	5.119	4.985	5.187	5.73	5.363	4.74	4.745	5.635	0	6.467	2.44	6.055	0	6.249	-0.08	3.814	2.562	0	4.268	3.142	1.71
TaLTP2.88	-1.97	1.489	-2.26	0	-2.28	0	0	-2.73	0	0	0	1.651	4.441	5.809	0	1.28	0	4.501	0.401	3.127	-2.78	-1.75	-0.82	3.218	-0.91
TaLTP2.89	0	0	0	0	0	0	0	0	0	0	0	-2.23	0	-2.4	0	0	0	-0.64	0	0	0	0	0	0	0
TaLTP2.9	1.839	0	1.377	1.99	2.414	1.505	1.157	0	2.384	2.389	-2.57	5.546	-2.3	6.258	-2.48	3.083	0	5.727	-0.99	5.07	0.141	3.88	0.515	-0.97	0
TaLTP2.90	0	0	0	0	0	0	0	0	0	0	0	0	0	0	0	0	0	0	0	0	0	0	0	0	0
TaLTP2.91	0	-3.39	0	0	-3.15	-2.87	0	-3.03	-3	0	-1.63	0	7.453	-4.1	4.755	4.707	-0.09	0	5.565	5.664	6.514	6.302	-1.54	7.781	2.91
TaLTP2.92	0	-3.39	0	0	-3.15	-2.87	0	-3.03	-3	0	-1.63	0	7.453	-4.1	4.755	4.707	-0.09	0	5.565	5.664	6.514	6.302	-1.54	7.781	2.91
TaLTP2.93	-1.75	0.687	-1.34	1.127	-0.13	1.374	0.941	2.501	1.525	-0.53	1.189	-3.08	5.723	-3.38	6.055	4.464	-0.69	-3.1	5.547	5.343	6.712	4.346	4.966	6.866	0.754
TaLTP2.94	-1.75	0.687	-1.34	1.127	-0.13	1.374	0.941	2.501	1.525	-0.53	1.189	-3.08	5.723	-3.38	6.055	4.464	-0.69	-3.1	5.547	5.343	6.712	4.346	4.966	6.866	0.754
TaLTP2.95	0	-4.39	0	-3.15	0	0	-2.03	-2.03	-1.83	0	-2.63	-2.02	1.363	-4.52	3.709	1.889	-1.82	-2.04	1.86	2.498	4.45	0.795	1.271	4.923	-0.82
TaLTP2.96	0	-5.39	0	-3.15	-1.98	-3.28	-3.62	0.373	-1.83	-3.82	-4.22	-4.35	1.663	-4.52	3.883	3.061	-1.82	-1.36	3.134	3.247	5.61	-2.26	1.107	5.971	-3.52
TaLTP2.97	0	0	0	0	0	-3.87	0	0	0	0	0	0	0	0	0	0	0	0	0	0	0	0	0	0	0
TaLTP2.98	0	-4.39	0	-3.15	0	0	-2.03	-2.03	-1.83	0	-2.63	-2.02	1.363	-4.52	3.709	1.889	-1.82	-2.04	1.86	2.498	4.45	0.795	1.271	4.923	-0.82
TaLTP2.99	-2.39	-1.99	-2.86	0.082	-0.6	-0.25	0.11	0.986	0.857	-1.29	-0.47	-2.58	3.912	-3.38	4.692	3.815	-0.58	-5.68	3.961	4.171	6.027	2.399	3.016	6.159	-0.38
TaLTPc.1	10.09	4.447	9.279	9.656	9.939	8.591	8.036	0	9.474	9.233	0.705	0	0	0	0	0	0	0	0	0	0	0	2.314	0	0
TaLTPc.2	9.356	4.737	8.323	9.082	9.049	7.571	7.428	-1.89	8.86	8.474	-1.07	0	0	0	0	0	0	0	0	0	0	0	1.88	0	0
TaLTPc.3	0	0	0	0	0	0	0	0	0	0	0	0	0	0	0	0	0	0	0	0	0	0	0	0	0
TaLTPc.4	12.43	5.773	12.38	12.15	12.75	11.23	10.72	-1.36	11.64	11.81	2.663	0	0	0	0	0	0	0	0	0	0	0	5.271	0	0
TaLTPc.5	10.66	4.359	9.958	10.79	10.58	9.399	9.266	0	10.57	9.846	1.701	0	0	0	0	0	0	0	0	0	0	0	4.143	0	0
TaLTPd.1	0	0	0	0	0	0	0	0	0	0	-2.7	0	0	-2.99	6.143	0	0	0	0	-2.79	0	0	0	0	4.321
TaLTPd.10	0	0	0	0	0	0	0	0	0	0	0	0	0	0	3.63	0	0	0	0	0	0	0	0	0	0.432
TaLTPd.11	0	0	0	0	0	0	0	0	0	0	0	0	0	0	5.22	0	0	0	0	0	0	0	0	0	1.965
TaLTPd.12	-3.3	10.66	0	-2.22	-1.05	-2.36	-1.7	2.247	-0.48	-2.89	-0.98	0	0	-3.6	0	0	0	-3.45	0	0	0	0	7.599	0	0
TaLTPd.13	0	0	0	0	0	0	0	0	0	0	0	0	0	0	-2.88	0	0	0	0	0	0	0	0	0	0
TaLTPd.14	0	0	0	0	0	0	0	0	0	0	0	0	0	0	5.095	0	0	0	0	0	0	0	0	0	1.143
TaLTPd.15	0	0	0	0	0	0	0	0	0	0	0	0	0	0	6.393	0	-1.3	0	0	0	0	0	0	0	6.485
TaLTPd.16	0	0	0	0	0	0	0	0	-1.97	0	-2.11	0	0	0	6.101	0	0	0	0	0	0	0	0	0	5.235
TaLTPd.17	0	0	0	0	0	0	0	0	0	0	0	0	0	0	2.135	0	0	0	0	0	0	0	0	0	0
TaLTPd.18	0	0	0	0	0	0	0	0	-1.97	0	-3.7	0	0	0	6.285	0	0	0	-2.7	0	0	0	0	0	4.473
TaLTPd.19	0	10.77	0	0	0	0	0	2.013	0	0	-1.1	0	-3.12	0	0	-3.46	0	0	-3.43	0	-3.28	-3.27	6.669	-3.39	0
TaLTPd.2	0	0	0	0	0	0	0	0	0	0	0	0	0	0	-1.6	0	0	0	0	0	0	0	0	0	1.728
TaLTPd.20	0	4.795	0	0	0	0	0	-3.62	0	0	0	0	0	0	0	0	0	0	-4.83	0	0	0	-4.71	0	0
TaLTPd.21	0	5.306	0	0	0	0	0	0	0	0	-4.35	-4.46	0	0	0	0	0	0	0	0	0	0	-4.25	0	0
TaLTPd.22	2.694	4.152	2.034	0.054	3.545	1.84	1.714	0.298	2.518	2.307	0.9	5.191	0	5.426	2.026	3.367	-1.37	4.935	-1.36	4.096	-1.63	5.396	-0.08	0.38	1.404
TaLTPd.23	-3.46	8.027	0	-1.8	-1.8	-0.95	0	0.296	0	0	-2.48	0	0	0	0	-3.51	0	0	0	0	0	0	0.978	0	0
TaLTPd.24	0	0	0	0	-2.43	0	0	0	0	0	0	-0.88	0	0	-0.16	0	0	-0.61	0	0	-2.94	0	0	0	-2.38
TaLTPd.25	0	4.995	0	0	0	0	0	-4.09	0	0	0	-5.98	0	-3.17	0	0	0	0	-5.88	0	0	0	0	0	0
TaLTPd.26	0	2.592	0	0	0	0	0	-6.26	0	0	0	0	0	0	0	0	0	0	0	0	0	0	0	0	0
TaLTPd.27	0	2.547	0	0	0	0	0	-6.26	0	0	0	0	0	0	0	0	0	0	0	0	0	0	0	0	0
TaLTPd.28	0	2.592	0	0	0	0	0	-6.26	0	0	0	0	0	0	0	0	0	0	0	0	0	0	0	0	0
TaLTPd.29	0	4.995	0	0	0	0	0	-4.09	0	0	0	-5.98	0	-3.17	0	0	0	0	-5.88	0	0	0	0	0	0
TaLTPd.3	0	0	0	0	0	0	0	0	0	0	0	0	0	0	0	0	0	0	0	0	0	0	0	0	-1.03
TaLTPd.30	0	5.851	-2.74	0	-2.74	0	0	-1.95	0	-3.98	0	0	0	0	-2.76	0	0	0	-1.67	0	0	0	-2.36	0	0
TaLTPd.31	-1.76	3.955	-0.66	-2.26	0.025	-2.4	-1.74	-3.15	-1.2	-0.76	-1.17	6.21	0	6.1	0	2.665	0	5.921	-0.35	3.943	0	4.54	-1.24	-3.31	-3.63
TaLTPd.32	-3.46	6.049	0	0	-1.8	0	-2.87	0	-2.64	0	0	0	-3.18	0	0	0	-2.08	0	0	0	0	0	-2.38	0	-3.76
TaLTPd.33	0.726	0	-0.06	1.003	1.058	-0.11	-0.45	0	0.911	0.142	0	3.217	0	0.121	0	-1.51	0	1.542	0	0.68	-0.91	0	0	0	0
TaLTPd.34	0	6.339	0	0	0	0	0	-3.15	0	0	0	0	0	0	0	0	0	0	0	-4.4	0	0	-0.66	0	0
TaLTPd.35	0	-0.81	0	0	0	0	0	0	0	0	0	0	0	0	0	0	0	0	0	0	0	0	0	0	0
TaLTPd.36	0.395	2.777	1.082	0.585	1.222	-0.36	-0.37	0	-0.67	-0.19	0.226	6.668	0	5.913	-2.61	2.593	-1.9	4.915	-0.99	4.784	-1.58	5.315	1.799	-1.68	-0.01
TaLTPd.37	-3.46	8.407	-1.2	-0.21	0	-1.53	0	-0.29	-0.64	-2.06	0	0	0	0	-1.78	-2.51	0	-3.66	0	-3.55	0	0	1.642	0	-3.76
TaLTPd.38	-2.47	-2.07	0	0	-2.81	-1.54	0	0	0	-3.06	0	0	0	0	0	0	0	0	0	-3.56	0	0	-3.39	0	0
TaLTPd.39	0	0	0	0	0	0	0	0	0	0	0	0	0	0	3.916	0	0	0	0	0	0	0	0	0	0

TaLTPd.4	0	0	0	0	0	0	0	0	0	0	-2.7	0	0	0	4.891	0	0	0	0	0	0	0	0	0	4.675
TaLTPd.40	-2.44	-0.16	-1.76	-0.03	0.548	0.498	-1.84	-0.94	-2.61	-2.03	0.947	3.804	0.176	3.112	0	4.081	0	2.821	-0.14	4.591	2.084	5.538	0.973	1.793	-2.73
TaLTPd.41	-2.7	-3.9	-3.03	-2.62	-3.05	-2.78	0	0	0	-3.3	0	0	0	0	-2.04	0	0	0	-2.16	0	0	0	0	0	0
TaLTPd.42	0	0	0	0	0	0	0	0	0	0	0	0	0	0	5.01	0	0	0	0	0	0	0	0	0	-2.99
TaLTPd.43	-2.15	-1.62	-0.76	-1.07	0.613	-0.5	-3.16	-2.01	-2.91	-1.34	0.919	2.638	-3.47	2.005	-2.08	4.451	-1.39	1.912	-2.79	4.058	-0.06	3.444	1.225	0.16	-3.05
TaLTPd.44	-2.64	-2.84	0	0	-1.99	-1.73	-3.06	0	0	0	0	0	0	0	0	0	0	0	0	0	0	0	0	0	-3.95
TaLTPd.45	0	0	0	0	0	0	0	0	0	0	0	0	0	0	4.59	0	0	0	0	0	0	0	0	0	0
TaLTPd.46	0	0	0	0	0	0	0	0	0	0	-2.66	0	0	0	3.979	0	0	-2.7	0	0	0	0	0	0	0
TaLTPd.47	0.904	0.965	0.008	1.139	1.492	1.006	-0.33	0.518	-0.86	0.145	2.173	2.726	-1.96	3.743	-3.15	5.71	0.139	2.597	-0.26	5.762	1.638	5.432	2.397	1.474	-0.55
TaLTPd.48	0	0	0	0	0	0	0	0	0	0	-0.7	0	0	0	4.39	0	-1.3	0	0	0	0	0	0	0	5.821
TaLTPd.49	0	0	0	0	0	0	0	0	0	0	-1.11	0	0	0	4.877	0	-0.72	0	0	0	0	0	0	0	6.89
TaLTPd.5	0	9.849	0	0	-2.48	-2.2	-2.54	0.861	0	0	-1.13	-1.68	0	-0.43	0	0	0	-3.26	-3.14	0	0	0	6.974	0	0
TaLTPd.50	0	0	0	0	0	-2.41	-2.75	0	-1.53	-2.94	-2.35	4.552	0	3.21	0	-1.07	0	2.576	0	-2.42	-3.21	0	0	0	-1.32
TaLTPd.51	2.773	5.075	2.075	2.39	2.778	2.74	1.776	1.712	1.878	1.924	2.601	6.647	-1.17	9.515	-1.36	6.071	0	8.405	4.367	7.02	0.51	8.297	4.17	2.782	0
TaLTPd.52	0	0	0	0	0	0	0	0	0	0	0	0	0	0	0	0	0	0	0	0	0	0	0	0	0
TaLTPd.53	0	2.547	0	0	0	0	0	0	0	0	0	0	0	0	0	0	0	0	0	0	0	0	0	0	0
TaLTPd.54	0	6.087	0	0	0	0	0	-1.83	0	0	0	0	0	0	0	0	0	0	0	0	0	0	-4.24	0	0
TaLTPd.55	2.793	3.961	2.731	3.059	2.995	3.132	2.324	1.317	2.818	2.394	3.356	6.953	0	9.006	-3.4	6.15	0	8.272	2.813	6.942	2.208	8.071	3.255	2.745	0.009
TaLTPd.56	1.9	3.804	1.58	2.274	2.33	1.34	0.939	0.913	2.081	1.626	1.703	6.78	-0.3	9.84	0	6.432	0	9.151	2.236	7.328	2.043	8.771	2.125	4.001	-1.9
TaLTPd.57	0	0	0	0	0	0	0	0	0	0	0	0	0	0	0	0	0	0	0	0	0	0	0	0	0
TaLTPd.6	0	0	0	0	0	0	0	0	0	0	0	0	0	0	5.203	0	0	0	0	0	0	0	0	-1.09	-2.03
TaLTPd.7	0	0	0	0	0	0	0	0	0	0	0	0	0	0	5.154	0	0	0	0	0	0	0	0	0	4.106
TaLTPd.8	0	0	0	0	0	0	0	0	0	0	-2.7	0	0	0	5.014	0	-1.3	0	0	0	0	0	0	0	7.276
TaLTPd.9	0	0	0	0	0	0	0	0	0	0	0	0	0	0	3.828	0	-2.3	0	0	0	0	0	0	0	4.793
TaLTPg.1	0.803	3.192	1.303	0.051	1.763	0.389	-0.06	-3.09	0.718	0.897	-0.47	5.792	-0.27	5.827	-1.84	1.838	0	5.45	0.029	2.737	2.187	0.26	0.578	0.892	2.903
TaLTPg.10	0	-4.39	0	-3.08	0	0	0	0	0	0	0	0.58	0	1.763	0	-2.25	0	1.533	0	-4.29	-0.51	-4.09	0	-1.61	-4.49
TaLTPg.11	3.553	3.384	3.054	3.593	3.463	3.907	3.159	3.961	3.521	2.803	1.61	2.062	0	2.613	2.56	2.836	-2.78	3.029	1.674	1.884	1.95	-1.04	1.713	0.74	0.918
TaLTPg.12	0	0	0	0	0	0	0	0	0	0	0	0	0	0	0	0	0	0	0.401	0	0	0	0	0	0
TaLTPg.13	-2.86	-0.79	-1.23	-1.79	0.352	3.755	-2.91	0	-1.3	3.15	0.512	0	-4.23	0	-4.42	0	0	0	0	0	-4.4	0	-0.86	0	-4.79
TaLTPg.14	0	0.561	0	0	0	0	0	0	0	-4.01	0	1.248	0	2.972	-3.38	-1.34	0	2.014	-4.51	-1.09	-0.65	-4.35	-4.4	-2.13	-2.75
TaLTPg.15	3.777	4.98	3.131	3.712	3.832	3.816	3.165	4.547	3.196	3.359	1.386	1.524	-2.87	2.846	2.862	1.858	-1.78	2.647	2.107	0.623	1.353	-0.95	1.534	-0.19	0.605
TaLTPg.16	0	0	-3.48	0	0	0	-3.57	0	0	0	0	0	0	0	-4.08	0	-3.8	0	0.177	0	0	0	-4.1	0	0
TaLTPg.17	-3.48	-1.56	-2.26	-2.4	0.398	2.835	-3.94	-4.39	0	2.717	0.642	-4.69	0	-2.08	0	0	0	0	0	0	0	0	-2.48	0	0
TaLTPg.18	0	-3.09	0	0	0	0	0	0	0	0	0	0.251	0	2.451	0	-1.53	0	1.823	-4.54	-3.57	-0.68	-3.38	-4.42	-1.67	-1.96
TaLTPg.19	2	-0.49	1.464	1.182	1.995	1.743	1.336	1.234	1.869	1.557	-0.71	-4.62	0	-2.82	-1.22	-2.52	-1.53	-4.78	-2.21	-4.56	-4.37	0	-4.41	-4.47	-2.44
TaLTPg.2	0.139	4.817	0.089	-0.2	0.317	0.198	0.392	0.919	0.433	-0.76	-1.7	0	-2.14	-5.08	0.476	-4.2	2.157	0	-0.68	-4.82	-1.54	-3.31	-2.22	-2.41	1.603
TaLTPg.20	2.755	4.312	1.864	2.749	2.854	2.461	2.593	3.789	2.744	1.731	0.693	0	-0.87	0	2.572	1.578	-4.11	0	1.133	0.487	3.727	-2.37	2.731	1.958	-2.76
TaLTPg.21	2.139	4.018	1.686	2.743	2.279	2.211	2.428	3.509	2.703	0.89	1.175	0	-2.04	0	2.464	0.95	-3.76	0	0.551	0.512	3.368	-2.69	2.205	1.368	0
TaLTPg.22	0.617	0.71	0.3	0.01	0.621	0.304	0.057	-0.86	0.763	-0.17	-1.6	-2.87	-2.86	-1.89	-2.63	-2.44	-1.55	-3.04	-3.19	0	0	-1.81	0	-2.71	-5
TaLTPg.23	2.182	3.385	1.717	2.266	2.173	2.308	2.707	3.211	2.49	1.425	1.2	0	-3.62	0	2.117	1.388	-4.12	-4.79	1.018	-0.08	3.306	-2.21	2.394	1.671	-2.18
TaLTPg.24	3.144	4.317	2.514	3.69	3.123	3.45	4.068	4.736	3.785	2.122	2.854	-2.24	0	-1.37	0.435	1.561	-1.73	-2.54	0.152	0.837	1.012	0	2.742	0.314	-0.51
TaLTPg.25	0.127	0.785	-0.06	-0.15	0.558	0.18	-0.68	-3.3	0.238	0.164	-0.59	2.496	-4.18	3.744	0	1.521	-4.09	2.855	-2.92	1.528	-0.35	0.698	1.003	-0.36	-4.74
TaLTPg.26	-1.56	0.711	-2.15	0.409	-1.15	-2.49	-0.82	-2.27	0.161	-2.98	-4.47	0	0	0	-3.34	-4.47	0	-3.14	0	-4.51	-1.51	0	-2.36	-3.42	0
TaLTPg.27	0	0	0	0	0	0	0	0	0	0	0	-4.5	0	0	0	0	0	-4.21	0	0	0	-5.45	0	-5.57	0
TaLTPg.28	0	0	0	0	0	0	0	0	0	0	0	-0.21	0	-1.36	0	-3.63	0	-1.45	0	0	-2.46	-4.23	-5.09	-4.35	0
TaLTPg.29	-3.4	-4.98	-1.64	-4.65	-3.22	-3.27	-4.19	0	-3.31	-2.46	0	0	0	0	0	0	0	0	0	0	0	0	0	0	0
TaLTPg.3	-2.04	2.152	-1.4	-2.28	-0.73	-1.9	-1.49	-1.67	-1.53	-1.97	-2.14	4.601	-0.23	5.559	0.254	1.803	-3.05	5.007	-0.3	1.629	1.335	-0.61	0.457	-0.09	-1.24
TaLTPg.30	3.692	0	3.238	2.753	3.85	2.507	2.76	-4.62	3.826	3.365	-0.74	0	0	0	0	0	0	0	0	0	0	0	-4.71	0	-5.06
TaLTPg.31	0	0	0	0	0	0	0	-3.43	-3.7	0	0	0.072	0	-0.18	-4.5	-4.63	0	-0.09	0	0	-1.48	-4.48	-4.52	-2.58	-4.87
TaLTPg.32	4.122	6.772	3.712	4.537	3.959	4.173	4.696	5.411	4.32	3.52	3.485	0.968	0	2.116	3.277	3.192	2.892	1.686	1.726	2.383	2.421	-4.44	3.827	1.701	1.736
TaLTPg.33	0	-4.13	0	0	0	0	0	0	0	0	0	0	0	0	0	0	0	0	0	0	0	0	0	0	0
TaLTPg.34	#####	0	0	0	0	0	0	0	0	0	0	0	0	0	0	0	0	0	0	0	0	0	0	0	0
TaLTPg.35	0	0	0	0	0	0	0	-3.47	0	0	0	1.938	0	1.899	0	-2.67	0	1.514	0	0	-0.06	-4.52	-4.56	-1.62	-2.59
TaLTPg.36	-0.72	2.332	-2.15	0.516	-0.65	-1.91	-1.5	-0.06	0.215	-2.65	-2.14	-3.57	-3.15	-3.76	-4.34	-3.47	0	-4.73	-2.48	-1.7	-0.27	-3.32	-1.27	-1.25	0
TaLTPg.37	-1.29	-0.49	-1.95	0	-0.15	-2.69	-0.71	-3.46	-0.2	-2.21	-0.84	1.141	-3.34	3.386	-3.53	1.082	0	3.557	-2.07	2.48	-0.92	1.149	1.312	-0.29	0
TaLTPg.38	2.981	6.542	2.779	3.238	3.048	3.425	3.774	5.457	3.243	2.264	2.605	0.228	0	0.603	2.97	1.584	1.546	0.394	1.008	0.175	1.33	0	3.033	0.914	1.103
TaLTPg.39	0	2.552	-3.62	0	0	0	0	0	0	-3.89	0	0	0	0	0	0	0	0	0	0	0	0	-1.62	0	0
TaLTPg.4	1.454	6.106	0.821	1.6	1.562	1.108	1.456	0.954	1.366	0.767	-0.2	-4.88	-4.46	0	0.742	-1.78	2.7	0	0.156	-3.23	-2.31	-2.31	0.186	-2.73	2.929
TaLTPg.40	0	0	0	0	0	0	0	0	0	0	0	0.512	0	2.604	0	-3.3	0	1.462	0	0	-3.14	0	0	-4.24	0
TaLTPg.41	0	0	0	0	0	0	0	0	0	0	0	-0.23	0	1.564	0	-3.3	0	0.345	0	-4.33	-2.55	-4.14	0	-4.24	0
TaLTPg.42	0	0	0	-3.12	0	0	0	-3.37	0	0	0	1.19	0	2.309	0	-1.39	0	2.184	0	-4.33	-1.14	-4.14	0	-3.24	0
TaLTPg.43	0	0	0	0	0	0	0	0	0	0	0	1.512	0	1.662	0	0.227	0	1.922	0	-3.33	0.905	-4.14	-0.86	0.006	0
TaLTPg.44	0	-3.75	0	0	0	0	0	0	0	0	0	-1.71	0	-0.89	-1.89	0	0	-0.08	0	0	-3.45	0	-3.49	-2.56	0
TaLTPg.45	0	0	0	0	0	0	0	0	0	0	0	0	0	0	0	0	0	-4.69	0	0	0	0	0	0	0
TaLTPg.46	0	-3.61	0	0	0	0	0	0	0	0	0	0	0	0	0	0	0	-4.72	0	0	0	0	0	0	0
TaLTPg.47	-2.12	8.041	0	0	-3.81	0	-3.89	-2.34	-2.61	-4.05	0	0	2.636	0	-0.09	0.409	0	0	2.078	-2.58	4.462	0.653	-2.85	2.076	-3.78
TaLTPg.48	-0.01	6.974	-2.15	-1.71	0.435	-0.35	-0.82	3.108	-0.08	-2.98	-3.47	0	2.988	-2.18	3.722	-0.08	0.747	0	3.566	1.077	3.429	0.144	3.051	1.93	1.782
TaLTPg.49	0	2.622	-4.52	0	0	0	0	0	0	-4.77	0	0	0	0	0	0	0	0	0	0	0	0	-3.56	0	0
TaLTPg.5	0.171	3.663	0.227	0.315	1.323	-0.34	-0.67	-2.11	0.694	-0.02	-4.31	4.751	1.178	5.652	-0.1	0.852	-3.91	4.974	0.325	1.289	0.793	-0.7	-0.3	-0.09	0.3
TaLTPg.50	0	0	0	-3.1	0	0	0	0	0	0	0	1.057	0	1.591	0	-1.46	0	1.795	0	0	-2.11	-4.11	-4.15	-1.89	0
TaLTPg.51	-4.2	-4.43	0	0	0	-3.32	-2.65	0	-3.37	0	0	2.128	0	2.285	0	-0.05	0	2.571	-4.3	-2.33	0.183	0	-2.6	-1.07	0
TaLTPg.52	0	0	0	0	0	0	-3.61	0	0	0	0	-1.77	-2.93	0	0	0	0	-1.33	0	0	0	0	-4.15	0	0

TaLTPg.53	0	0	0	0	0	0	0	0	0	0	0	0.646	0	0.641	0	-1.42	0	0.967	0	0	-0.46	0	0	-4.37	0
TaLTPg.54	0	0	0	0	0	0	0	0	0	0	0	-13.6	0	0	0	-13.7	0	0	0	0	0	0	0	0	0
TaLTPg.55	0	-1.92	0	0	0	0	0	0	0	0	0	2.431	0	1.153	0	-0.9	0	1.848	0	0	0.547	-2.62	-4.25	-1.14	0
TaLTPg.56	0	-3.55	0	0	0	-3.44	0	0	0	0	0	-0.12	0	0.363	-0.81	0	0	0.974	0	0	-2.66	-4.23	-4.29	0	-4.67
TaLTPg.57	0	-3.63	0	0	0	0	-3.84	0	0	0	0	-3	0	-4.78	-2.77	0	0	-2.74	0	0	-4.33	0	-4.37	0	0
TaLTPg.58	-1.41	5.597	-1.35	-1.92	-0.77	-1.1	-1.85	-1.87	-1.59	-3.6	0	0	-0.95	0	-1.62	0.441	-3.67	0	1.01	-0.53	2.538	1.213	-1.16	-0.03	0
TaLTPg.59	-0.22	7.201	-0.54	-0.68	-0.01	0.098	-1.21	2.166	-1.2	-0.95	-1.27	-3.55	3.256	-4.74	3.3	0.762	0.355	-4.7	3.036	-0.03	3.078	1.294	2.978	2.023	-0.69
TaLTPg.6	0.63	5.733	0.884	1.259	1.239	0.947	1.22	1.12	1.101	-0.08	-0.66	0	-2.47	-5.09	1.385	-1.4	3.329	0	-0	-4.83	-2.83	-4.05	-0.82	-1.74	2.827
TaLTPg.60	0	2.809	0	0	0	0	0	0	0	0	0	0	0	-2.54	0	0	0	-4.83	0	0	0	0	-1.76	0	0
TaLTPg.61	0	0	0	0	0	0	0	0	0	0	0	0.555	0	1.083	0	-0.84	0	0.667	0	-2.34	-0.34	-3.14	-3.19	-1.25	0
TaLTPg.62	0	0	0	0	0	0	0	0	0	0	0	1.531	0	1.165	0	-1.72	0	0.815	0	0	-0.14	-4.14	0	0	0
TaLTPg.63	0	0	0	0	0	0	0	0	0	0	0	-0.44	0	-0.72	0	-2.11	0	0.359	0	0	-0.19	-2.69	-2.73	-1.57	0
TaLTPg.64	0	0	0	0	0	0	0	0	0	0	0	2.121	0	2.494	0	-0.38	0	2.303	0	-3.37	-0.72	-3.18	0	-2.7	0
TaLTPg.65	0	0	0	0	0	-4.24	0	0	0	0	0	-1.86	0	-0.15	-2.76	0	0	-0.36	0	0	-3.47	-5.05	-5.1	-4.16	-5.46
TaLTPg.66	0	0	0	0	0	0	0	0	0	0	0	-2.72	0	-1.91	0	0	0	-2.86	0	0	0	0	0	-4.15	0
TaLTPg.67	-2.47	6.026	0	-2.39	-1.03	-2.01	-3.93	-1.79	-2.64	-4.08	0	0	0.068	0	-2.12	0.914	-2.17	0	2E-04	-1.45	4.069	0.099	-1.88	2.242	0
TaLTPg.68	-1.2	6.148	-2.73	-0.12	-2.73	-0.27	-0.65	2.225	-0.54	-1.65	-0.77	0	2.608	-4.76	2.938	1.021	-1.25	-3.14	2.154	1.015	2.978	0.385	2.165	2.121	-1.01
TaLTPg.69	-4.32	-4.56	0	0	0	0	0	0	0	0	0	0	-4.09	0	-0.96	0	0	-3.67	-0.96	0	0	-4.26	0	-4.36	1.93
TaLTPg.7	3.77	4.253	2.846	3.706	3.704	3.658	3.338	4.007	3.533	3.145	1.802	2.221	-2.88	2.359	3.2	2.725	-0.1	2.755	2.497	1.951	1.936	0.308	2.527	0.701	1.275
TaLTPg.70	-2.29	-0.32	-2.54	-0.74	-1.96	-1.52	-1.19	-1.3	-0.77	-4.01	-2.5	-0.44	-0.18	1.084	0.328	-1.6	0.992	0.696	-0.38	0.706	-3.35	-2.54	-2.39	-0.75	-1.16
TaLTPg.71	-5.94	-2.14	-3.98	0	-4.72	-2.48	-3.39	-3.26	-2.53	0	-4.45	1.908	-3.55	1.588	-2.33	0.213	-2.05	0.855	-1.46	2.042	-0.72	-0.6	-3.02	-0.05	-0.89
TaLTPg.72	3.31	3.296	3.11	2.449	3.485	3.144	2.741	4.47	2.876	2.734	2.106	2.969	3.67	3.063	3.935	3.794	-2.8	2.509	2.448	2.694	4.476	2.745	2.262	3.006	1.355
TaLTPg.73	0	0	0	0	0	0	0	0	0	0	0	1.11	0	0.598	0	-1.7	0	-0.13	0	-3.32	-0.22	-4.12	0	-1.91	0
TaLTPg.74	-1.13	2.615	-1.45	-0.96	-0.55	-1.89	-1.69	-0.67	-1.53	-2.38	-3.45	1.987	-2.81	2.927	-1.52	1.348	0.651	2.313	-0.51	1.946	-0.98	0.627	-2.18	-1.01	-1
TaLTPg.75	-4.5	0	0	0	0	0	0	0	0	0	0	-0.32	0	0.268	0	-0.91	0	-0.78	0	-4.64	-0.45	-2.13	-3.5	-1.39	0
TaLTPg.76	0	-3.65	0	0	0	0	0	0	0	0	0	0	0	0	0	0	0	0	0	0	0	0	0	0	0
TaLTPg.77	4.698	7.7	4.631	4.786	4.857	4.764	4.814	5.223	4.402	4.026	3.29	0	3.357	-4.6	4.198	3.363	5.656	-4.55	3.233	2.472	4.698	2.664	4.48	3.832	2.405
TaLTPg.78	-2.75	-4.3	0	0	-3.44	-1.6	-3.52	-3.96	-3.25	0	0	2.409	0	-0.2	-4.03	0.535	-0.43	1.56	-2.58	0.442	-3.01	0	-3.05	-1.79	-1.41
TaLTPg.79	1.603	0.686	1.703	0.929	1.891	1.282	1.249	2.728	1.009	1.108	0.622	3.034	2.822	2.407	2.981	0.744	2.215	2.314	0.608	2.363	0.086	-0.57	-0.28	1.303	2.691
TaLTPg.8	0	0	0	0	0	0	0	-2.73	-0.56	0	0	0	0	0	0	0	-0.94	0	1.664	0	0	0	0	0	0
TaLTPg.80	0	-3.56	0	0	0	0	0	0	0	0	0	0	0	0	0	0	0	0	0	0	0	0	0	0	0
TaLTPg.81	3.743	7.236	3.547	3.606	3.896	3.517	3.771	4.252	3.336	2.968	2.59	0	2.452	0	3.741	3.306	5.33	-2.97	2.275	2.236	4.749	2.285	3.76	3.884	1.59
TaLTPg.82	0	0	0	0	0	0	-2.34	0	-3.08	0	-2.97	2.997	0	2.284	-3.85	1.055	0.599	2.608	-3.98	0.618	0.343	-2.82	-3.87	-2.93	-3.23
TaLTPg.83	0	-0.8	0	0	0	0	0	0	0	0	0	0	0	0	0	0	0	-3.61	0	0	0	0	0	0	0
TaLTPg.84	3.927	7.201	3.383	3.997	3.708	3.711	4.261	4.897	3.609	3.539	2.622	-2.55	1.369	-2.72	3.796	2.77	5.161	0	1.941	1.38	4.362	0.314	3.669	3.757	1.071
TaLTPg.85	0.124	0	0.175	-0.71	-1.73	-0.68	0.086	-2.27	-0.95	-0.98	-0.88	1.863	0	1.094	-1.34	1.229	-2.48	1.648	-2.15	0.814	-4.32	0	-0.04	-2.42	-4.71
TaLTPg.86	2.198	-0.13	1.867	1.035	2.153	1.71	1.503	3.239	2.034	1.818	1.128	2.935	0.656	2.013	3.96	0.729	2.044	1.762	1.667	1.331	1.334	-3.1	0.291	0.953	3.593
TaLTPg.9	-2.9	-1.73	-1.85	-3.41	0.395	2.764	-2.94	0	-0.49	2.634	0.802	0	0	0	0	0	-1.86	0	-4.6	-4.63	-4.44	0	-1.9	0	0

Supplementary Table S4. Motif enrichment analysis of *cis*-regulatory elements in anther-specific promoter regions.

Box	Consensus sequence	Enrichment *P*-value [a]	Reference
5256BOXLELAT5256	TGTGGTTATATA	-	Zhou, 1999
5659BOXLELAT5659	GAAWTTGTGA	-	Zhou, 1999
GBOX10NT	GCCACGTGCC	-	Ishige *et al.*, 1999
GTGANTG10	GTGA	-	Rogers *et al.*, 2001
POLLEN1LELAT52	AGAAA	6.72×10^{-3}	Bate and Twell, 1998
POLLEN2LELAT52	TCCACCATA	-	Bate and Twell, 1998
PSREGIONZMZM13	TCGGCCACTATTTCTACGGGCAGCCAGACAAA	-	Halmilton *et al*., 1998
QELEMENTZMZM13	AGGTCA	-	Halmilton *et al*., 1998
VOZATVPP	GCGTNNNNNNNACGC	-	Mitsuda *et al.,* 2004

[a] The enrichment *p*-value of the motif that are enriched in 17 the anther-specific gene promoter regions compared with promoter sequences of the remaining 444 identified *nsLTPs*. The *P*-value was calculated for the Wilcoxon rank-sum test implemented in AME tool (Analysis of Motif Enrichment).

Supplementary Table S5. Identification of wheat anther-specific *nsLTPs* orthologues in rice, maize and sorghum.

	**Wheat**		**Rice**[2]		**Maize**[3]		**Sorghum**[4]	
Gene	**Chr.**	**Position**	**Chr.**	**Position**	**Chr.**	**Position**	**Chr.**	**Position**
TaLTP2.25	chr1D	2.35E+08	Chr10	19288018	Chr5	23858169	Chr1	18191249
TaLTP2.14	chr1B	3.4E+08	Chr10	19288018	Chr5	23858169	Chr1	18191249
TaLTP2.1	chr1A	3.13E+08	Chr10	19288018	Chr5	23858169	Chr1	18191249
TaLTP2.214	chr7D	4.47E+08	-	-	-	-	-	-
TaLTP2.93	chr7A	4.99E+08	-	-	-	-	-	-
TaLTP2.198	chr7B	4.65E+08	-	-	-	-	-	-
TaLTPg.30	chr4B	13125606	Chr3	26073641	Chr2	48052056	Chr6	45983685
TaLTP1.25	chr5A	6.8E+08	Chr7	16294810	-	-	Chr2	17997167
TaLTP1.18	chr4B	6.42E+08	Chr7	16294810	-	-	Chr2	17997167
TaLTP1.21	chr4D	4.99E+08	Chr7	16294810	-	-	Chr2	17997167
TaLTPc.3	chr5D	4.12E+08	Chr9	20534165	Chr7	1.42E+08	Chr2	65407147
TaLTPc.1	chr5A	5.24E+08	Chr9	20534165	Chr7	1.42E+08	Chr2	65407147
TaLTPc.2	chr5B	4.97E+08	Chr9	20534165	Chr7	1.42E+08	Chr2	65407147
TaLTPc.4	chr7A	2.76E+08	Chr8	27364161	Chr4	1.95E+08	Chr7	61403378
TaLTPc.5	chr7B	2.35E+08	Chr8	27364161	Chr4	1.95E+08	Chr7	61403378
TaLTP1.14	chr3D	1.19E+08	Chr1	6541005	Chr8	25951054	Chr3	1451110
TaLTP1.12	chr3B	1.72E+08	Chr1	6541005	Chr8	25951054	Chr3	1451110
TaLTP1.10	chr3A	1.26E+08	Chr1	6541005	Chr8	25951054	Chr3	1451110
TaLTPg.19	chr3A	3.96E+08	Chr1	23919358	Chr10	15009187	Chr6	49389699

[2] Ouyang *et al.,* 2007

[3] Schnable *et al*., 2009

[4] McCormick *et al*., 2018

Supplementary Table S6. Primers used for qRT-PCR.

Gene	Forward primer (5' to 3')	Reverse primer (5' to 3')
TaGAPDH	TTCAACATCATTCCAAGCAGCA	CGGACAGCAAAACGACCAAG
TaActin	GACAATGGAACCGGAATGGTC	GTGTGATGCCAGATTTTCTCCAT
Ta13-3-3	ACGCAGCTACCTGTATCATTC	CGACGATGTCCACATGACC
TaLTP1.15	ATGAACCCTGAGATCGGACC	GGCCCTTGATTCAATCTTATAG
TaLTP2.102	TGCAACGTAGCATGTAGCAGC	TGGTGCTTGGGTTAGATCAAC
TaLTP2.93	GACAGCGACCTGCGTCAG	TGTCACAAACTGAAGGAGAAGG
TaLTP2.94	GCACTGCAACGTAGCATGTAG	GGTGCCTGGGTTGGATTAG
TaLTPc.1	CGAAGAAACGATGCGAGTC	GGCGAAGAGGCAGACATG
TaLTPc.2	ATGGGCATCATCAACAGCA	CAACAACGGCAGAATGACA
TaLTPc.3	AAGAAGACGATGATGAATAAGAGC	GACATGAGAGCCTCTAATGGC
TaLTPc.4	ATGAATAAGAAGCTCGATGGC	ATGAATAAGAAGCTCGATGGC
TaLTPc.5	AGGAGGAACTGAAGAAGACGAC	TGCAAATTGGTTTCATTACTATTG
TaLTP2.74	GACGGTTCTCAGCCTCGG	CCGGTAGCAAAGCAAGATCAC
TaLTP2.87	CGCGGTCACCTACTGCAAC	TCCACAGTTCCATGAAACTAACAG
TaLTP1.14	CGCAACGTACACAGAGGAAG	CATCGGGATAACAATTGAATTAC
TaLTP2.89	AGGCAACCGACGATTTCAG	AGCTACGTGATCCAGTTTTCG
TaLTP1.13	TACGGATGAACCCTGAGATG	AGGCCCTTGATTCAATCTTATAC

Supplementary Table S7. List of the 142 wheat nsLTPs with a high proline content at the N-terminal of the 8CM.

**Hybrid proline-rich proteins**[a]						
TaLTP2.3	TaLTP2.48	TaLTP2.122	TaLTP2.144	TaLTP2.193	TaLTP2.232	TaLTP2.261
TaLTP2.4	TaLTP2.49	TaLTP2.123	TaLTP2.149	TaLTP2.194	TaLTP2.233	TaLTP2.264
TaLTP2.5	TaLTP2.50	TaLTP2.124	TaLTP2.150	TaLTP2.195	TaLTP2.234	TaLTP2.265
TaLTP2.6	TaLTP2.51	TaLTP2.125	TaLTP2.152	TaLTP2.196	TaLTP2.235	TaLTP2.266
TaLTP2.7	TaLTP2.54	TaLTP2.126	TaLTP2.153	TaLTP2.201	TaLTP2.236	TaLTP2.269
TaLTP2.15	TaLTP2.55	TaLTP2.127	TaLTP2.157	TaLTP2.202	TaLTP2.237	TaLTP2.270
TaLTP2.16	TaLTP2.57	TaLTP2.128	TaLTP2.158	TaLTP2.203	TaLTP2.238	TaLTP2.271
TaLTP2.17	TaLTP2.58	TaLTP2.129	TaLTP2.162	TaLTP2.204	TaLTP2.239	TaLTP2.272
TaLTP2.18	TaLTP2.61	TaLTP2.130	TaLTP2.163	TaLTP2.205	TaLTP2.240	TaLTP2.273
TaLTP2.26	TaLTP2.62	TaLTP2.131	TaLTP2.164	TaLTP2.206	TaLTP2.244	TaLTP2.274
TaLTP2.27	TaLTP2.63	TaLTP2.132	TaLTP2.165	TaLTP2.207	TaLTP2.245	
TaLTP2.28	TaLTP2.64	TaLTP2.133	TaLTP2.166	TaLTP2.208	TaLTP2.246	
TaLTP2.29	TaLTP2.73	TaLTP2.134	TaLTP2.167	TaLTP2.209	TaLTP2.247	
TaLTP2.30	TaLTP2.77	TaLTP2.135	TaLTP2.172	TaLTP2.210	TaLTP2.248	
TaLTP2.31	TaLTP2.78	TaLTP2.136	TaLTP2.173	TaLTP2.211	TaLTP2.249	
TaLTP2.32	TaLTP2.82	TaLTP2.137	TaLTP2.174	TaLTP2.216	TaLTP2.250	
TaLTP2.39	TaLTP2.89	TaLTP2.138	TaLTP2.178	TaLTP2.217	TaLTP2.251	
TaLTP2.40	TaLTP2.115	TaLTP2.139	TaLTP2.179	TaLTP2.218	TaLTP2.252	
TaLTP2.41	TaLTP2.116	TaLTP2.140	TaLTP2.182	TaLTP2.219	TaLTP2.256	
TaLTP2.45	TaLTP2.118	TaLTP2.141	TaLTP2.185	TaLTP2.220	TaLTP2.257	
TaLTP2.46	TaLTP2.119	TaLTP2.142	TaLTP2.191	TaLTP2.221	TaLTP2.258	
TaLTP2.47	TaLTP2.120	TaLTP2.143	TaLTP2.192	TaLTP2.231	TaLTP2.259	

[a] Pfam domain PF14547 belonging to hydrophobic seed protein.
